# Supplementary material for: Macrocycle-based PROTACs selectively degrade cyclophilin A and inhibit HIV-1 and HCV
Source: Nat Commun. 2025 Feb 10;16:1484. doi: 10.1038/s41467-025-56317-8 (PMC11811207; doi:10.1038/s41467-025-56317-8)
Supplement: Supplementary file 1 — Supplementary Information [file 41467_2025_56317_MOESM1_ESM.pdf]

Supplementary Information

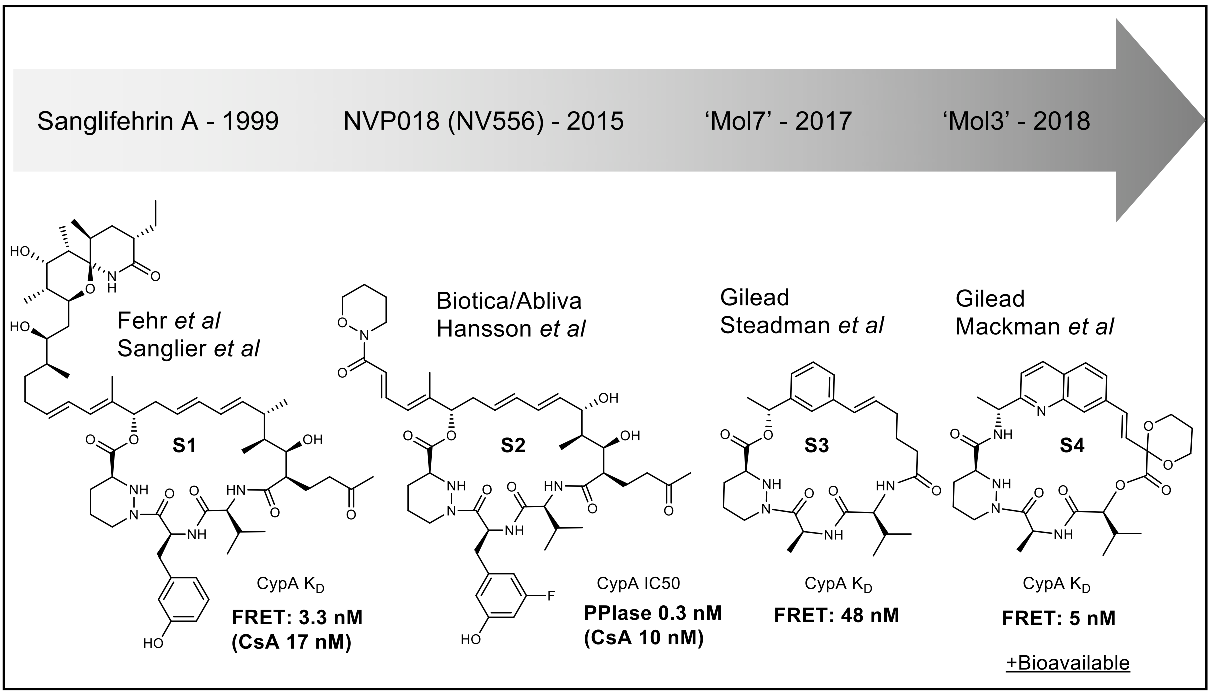

Supplementary Figure 1: Historical overview of sanglifehrin A (SfA) derived macrocycles as potent and bioavailable CypA inhibitors

SfA (**S1**) was isolated by Fehr and Sanglier in 1999[1, 2]. Degradation studies and semi-synthesis efforts to remove its spirocyclic extension led 15 years later to NVP018 (**S2**), a non-immunosuppressive and potent CypA inhibitor[3]. Recently, the simplification of the sanglifehrin scaffold to fully synthetic macrocycles (**S3** and **S4**) was reported, with retained low nanomolar affinity to CypA and improved bioavailability[4, 5].

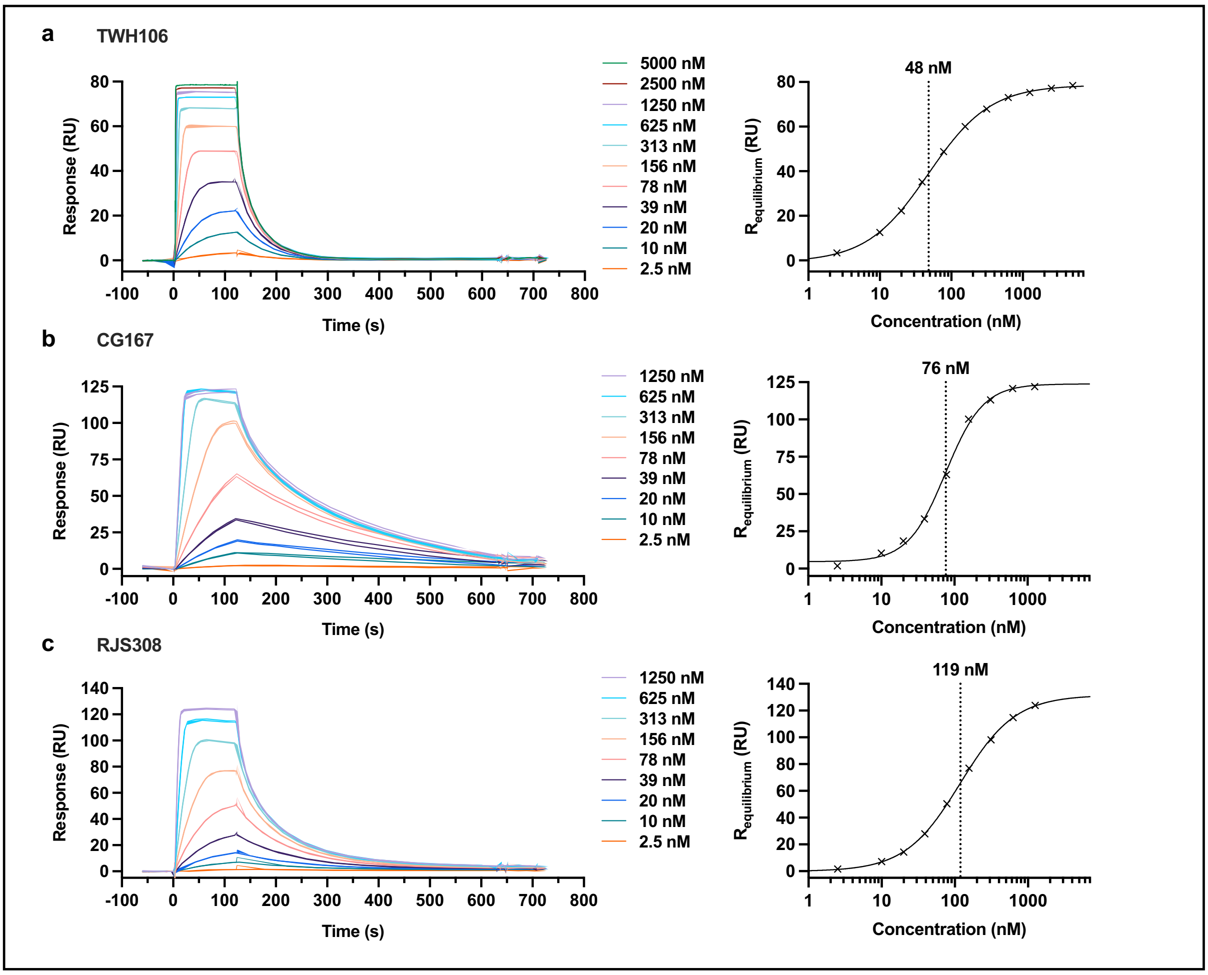

Supplementary Figure 2: Surface plasmon resonance data against CypA
a-c Dose-dependent binding (SPR) between (**a**) TWH106, (**b**) CG167, or (**c**) RJS308 (flow) and CypA (immobilised on chip), experiment run in duplicate. The steady state response of each curve from the sensorgrams (left) is plotted against concentration to give affinity curves (right), annotated with the dissociation constant (K_D) calculated from the sigmoid fit. Representative data from one of two independent experiments used to calculated K_D values in Table 1.

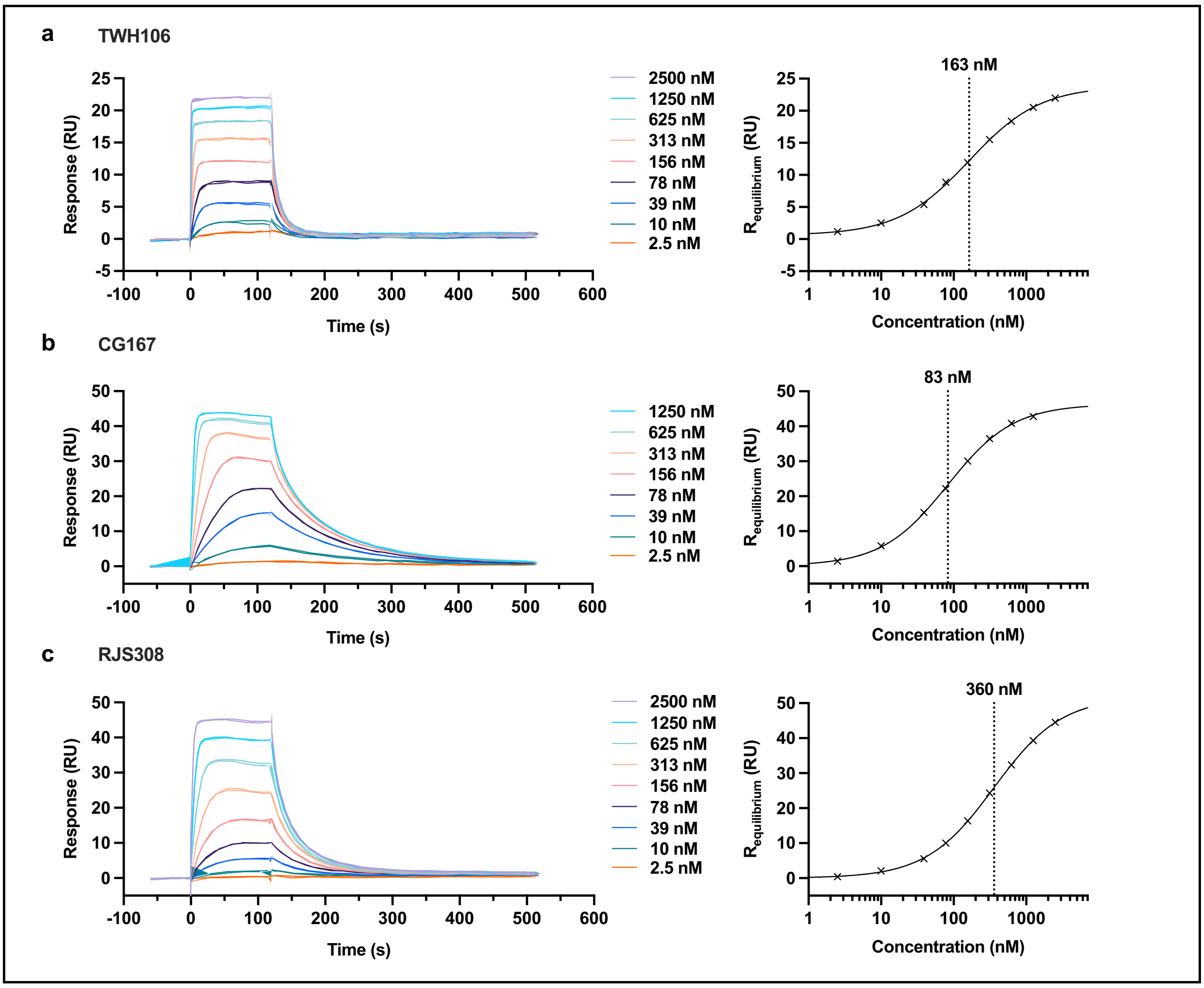

Supplementary Figure 3: Surface plasmon resonance data against CypB

a-c Dose-dependent binding (SPR) between (**a**) TWH106, (**b**) CG167, or (**c**) RJS308 (flow) and CypB (immobilised on chip), experiment run in duplicate. The steady state response of each curve from the sensorgrams (left) is plotted against concentration to give affinity curves (right), annotated with the dissociation constant (K_D) calculated from the sigmoid fit. Representative data from one of two independent experiments used to calculated K_D values in Table 1.

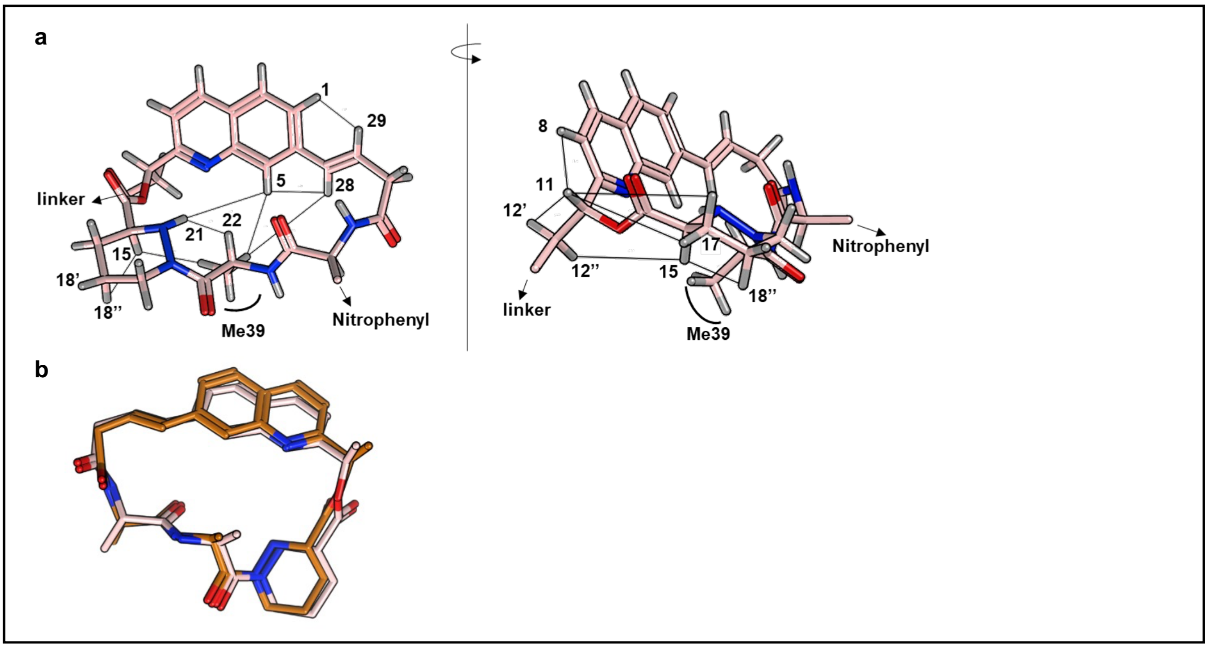

Supplementary Figure 4: NMR conformation of the CG167 macrocyclic core supports stereocenter configuration.

a Conformer of the truncated macrocyclic part of CG167 generated from NOE spectrum of CG167 (DMSO-d6, 600 MHz spectrometer) using MOE software, with strongest and relevant NOE correlations shown (black lines). The conformer best fitting the experimental data is shown. **b** The NMR structure of the CG167 macrocyclic core (light pink) overlayed with the docked TWH106 (orange) in CypA shows close alignment between solution structure and binding mode.

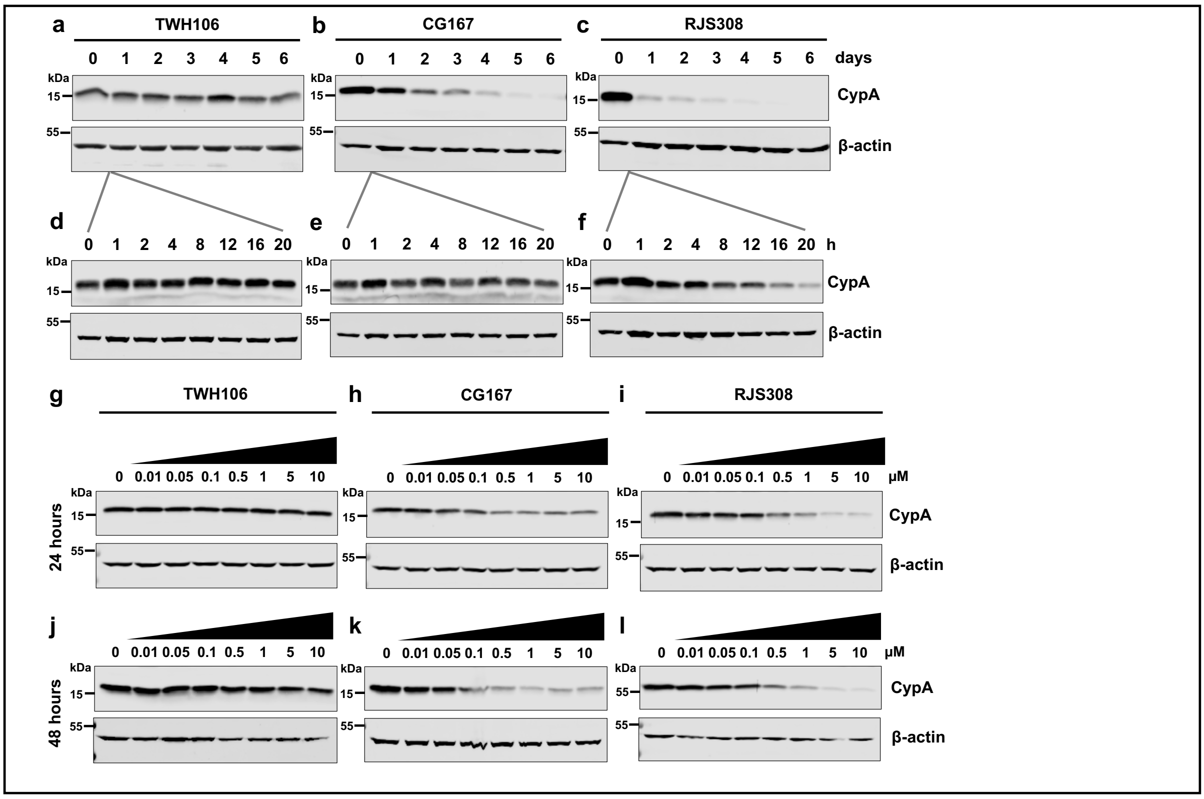

Supplementary Figure 5: PROTACs CG167 and RJS308 degrade CypA in a dose-dependent manner

a-f Immunoblots detecting CypA, or β-actin loading control, in Jurkat cells treated with 5 μM TWH106, CG167 or RJS308 for 6 days (**a-c**) or 20 h (**d-f**). **g-l** Immunoblots detecting CypA, or β-actin loading control, in Jurkat cells treated with 0.01 – 10 μM TWH106, CG167 or RJS308 for 24 h (**g-i**) or 48 h (**j-l**). Experiments were carried out independently to those in Fig. 3 and used to calculate adjusted CypA densities (Fig. 3g and n).

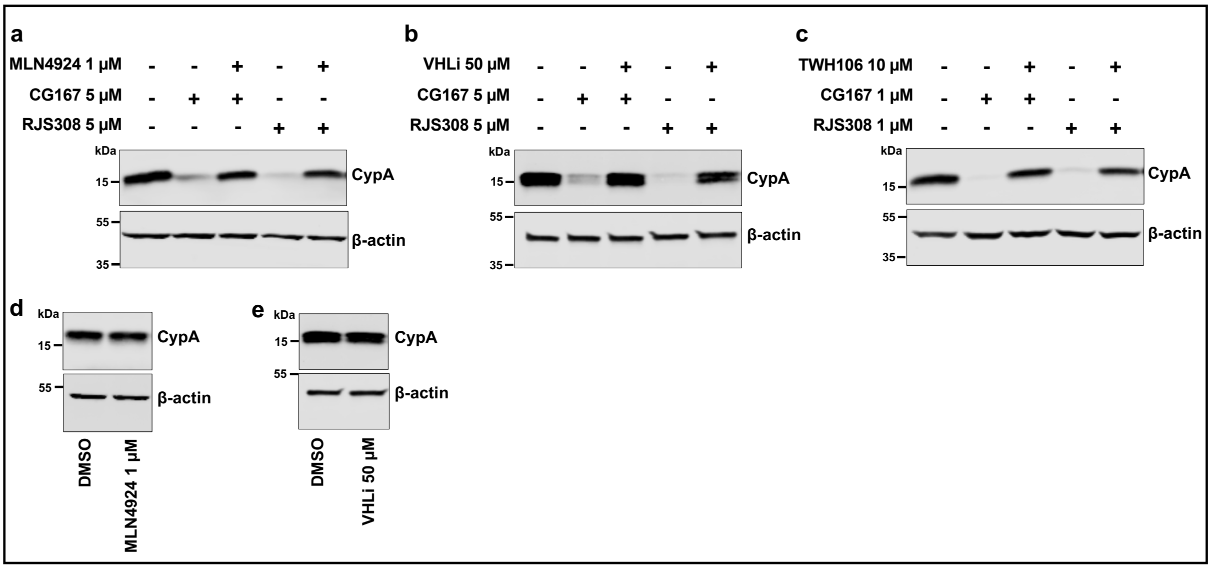

Supplementary Figure 6: Degradation of cyclophilin A by CG167 and RJS308 is via a PROTAC mechanism

a-e Immunoblots detecting CypA, or β-actin as loading control, in Jurkat cells pretreated with (**a**) 1 μM NEDD8-activating enzyme inhibitor MLN4924 for 6 h or (**b**) 50 μM VHL inhibitor VH298 (VHLi) for 2 h, followed by 5 μM PROTAC CG167 or RJS308 for 48 h, or treated with (**c**) 10 μM TWH106 and 1 μM PROTAC CG167 or RJS308 for 48 h, or treated with (**d**) MLN4924 as in (**a**) or with (**e**) VHLi as in (**b**). Experiments were carried out independently to those in Fig. 4 and used to calculate CypA densities (Fig. 4a-e)**.**

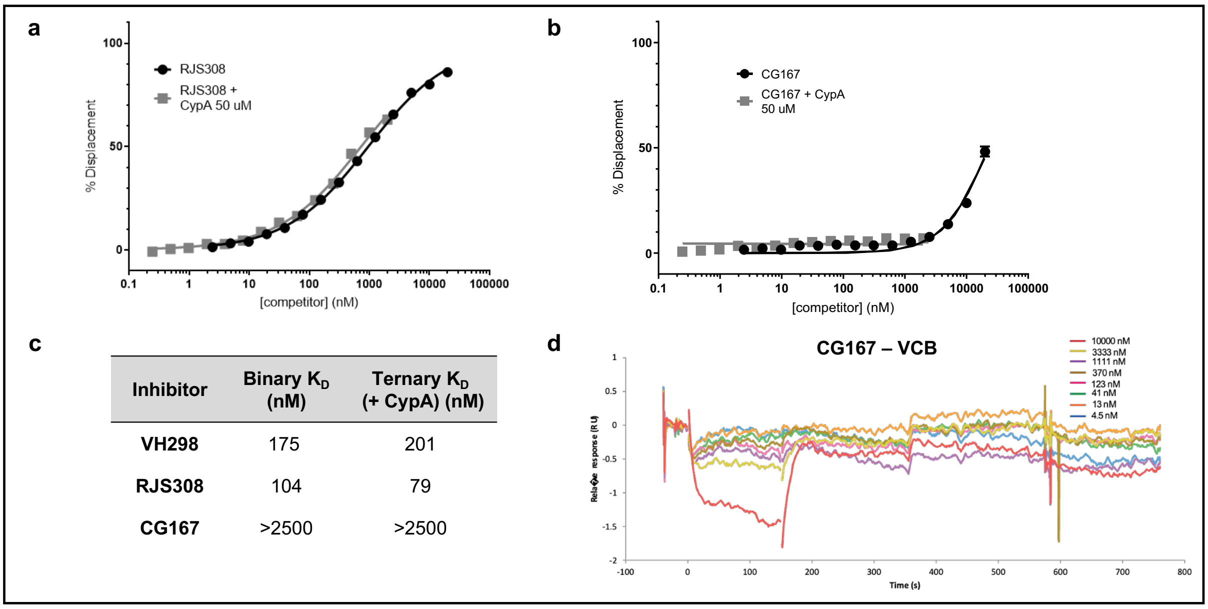

Inhibitor	Binary K_D (nM)	Ternary K_D (+ CypA) (nM)
VH298	175	201
RJS308	104	79
CG167	>2500	>2500

Supplementary Figure 7: Biochemical characterisation of CG167 and RJS308

a-b Displacement assay with fluorescent HIF-1α peptide bound to VCB displaced by (**a**) RJS308 or (**b**) CG167, alone or in complex with CypA, measured by fluorescence polarisation (n = 1 independent experiment performed in triplicate). **c** Binary and ternary dissociation constants for CG167, RJS308 or positive control VH298 calculated from FP experiments. **d** Surface plasmon resonance of CG167 (flow) against VCB (immobilised on chip).

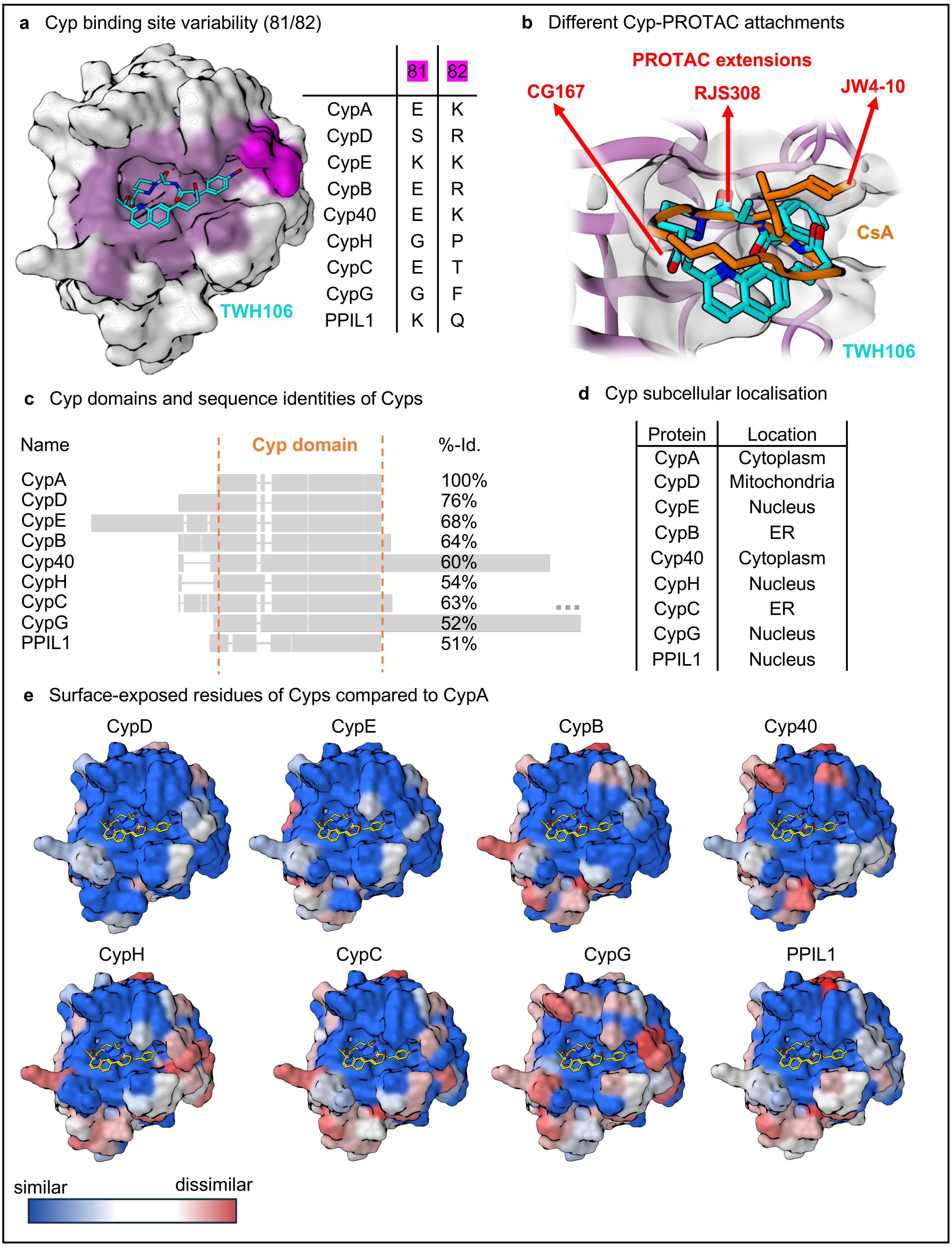

Supplementary Figure 8: Bioinformatic analysis of Cyps and PROTAC designs to understand PROTAC selectivity

(continued next page)

f Differences in lysine substitutions between Cyps

```
CypA     -VNPTVFFDIAVDGEPLGRVSFELFADKVPKTAENFRALSTGEKG--------FGYKGSC    51
CypD     SGNPLVYLDVDANGKPLGRVVLELKADVVPKTAENFRALCTGEKG--------FGYKGST    52
CypE     --NPQVYMDIKIGNKPAGRIQMLLRSDVVPMTAENFRCLCTHEKG--------FGFKGSS    50
CypB     KVTVKVYFDLRIGDEDVGRVIFGLFGKTVPKTVDNFVALATGEKG--------FGYKNSK    52
Cyp40    PSNPRVFFDVDIGGERVGRIVLELFADIVPKTAENFRALCTGEKGIGHTTGKPLHFKGCP    60
CypH     PVNPVVFFDVSIGGQEVGRMKIELFADVVPKTAENFRQFCTGEFRKD---GVPIGYKGST    57
CypC     SVTAKVFFDVRIGDKDVGRIVIGLFGKVVPKTVENFVALATGEKG--------YGYKGSK    52
CypG     VQRPRCFFDIAINNQPAGRVVFELFSDVCPKTCENFRCLCTGEKGTGKSTQKPLHYKSCL    60
PPIL1    WQPPNVYLETS-----MGIIVLELYWKHAPKTCKNFAELARRG-----------YYNGTK    44
               :::        * : : *  .  * * .**  :.               ::.

CypA     FHRIIPGFMCQGGDFTRHNGTGGKSIYGEKFEDENF-ILKHTGPGILSMANAGPNTNGSQ    110
CypD     FHRVIPSFMCQAGDFTNHNGTGGKSIYGSRFPDENF-TLKHVGPGVLSMANAGPNTNGSQ    111
CypE     FHRIIPQFMCQGGDFTNHNGTGGKSIYGKKFDDENF-ILKHTGPGLLSMANSGPNTNGSQ    109
CypB     FHRVIKDFMIQGGDFTRGDGTGGKSIYGERFPDENF-KLKHYGPGWVSMANAGKDTNGSQ    111
Cyp40    FHRIIKKFMIQGGDFSNQNGTGGESIYGEKFEDENF-HYKHDREGLLSMANAGRNTNGSQ    119
CypH     FHRVIKDFMIQGGDFVNGDGTGVASIYRGPFADENF-KLRHSAPGLLSMANSGPSTNGCQ    116
CypC     FHRVIKDFMIQGGDITTGDGTGGVSIYGETFPDENF-KLKHYGIGWVSMANAGPDTNGSQ    111
CypG     FHRVVKDFMVQGGDFSEGNGRGGESIYGGFFEDESF-AVKHNAAFLLSMANRGKDTNGSQ    119
PPIL1    FHRIIKDFMIQGGD-PTGTGRGGASIYGKQFEDELHPDLKFTGAGILAMANAGPDTNGSQ    103
         ***::  ** *.**     * *  ***   * **  .   :.     ::*** * .***.*

CypA     FFICTAKTEWLDGKHVVFGKVKEGMNIVEAMERFGSRN-GKTSKKITIADCGQLE  164
CypD     FFICTIKTDWLDGKHVVFGHVIEGMDVVKKIESFGSKS-GRTSKKIVITDCGQLS  165
CypE     FFLTCDKTDWLDGKHVVFGEVTEGLDVLRQIEAQGS-KDGKPKQKVIIADCGEYv  163
CypB     FFITTVKTAWLDGKHVVFGKVLEGMEVVRKVESTKTDSRDKPLKDVIIADCGKIE  166
Cyp40    FFITTVPTPHLDGKHVVFGQVIKGIGVARILENVEVKG-EKPAKLCVIAECGELK  173
CypH     FFITCSKCDWLDGKHVVFGKIIDGLLVMRKIENVPTGPNNKPKLPVVISQCGEM-  170
CypC     FFITLTKPTWLDGKHVVFGKVIDGMTVVHSIELQATDGHDRPLTNCSIINSGKID  166
CypG     FFITTKPTPHLDGHHVVFGQVISGQEVVREIENQKTDAASKPFAEVRILSCGELI  174
PPIL1    FFVTLAPTQWLDGKHTIFGRVCQGIGMVNRVGMVETNSQDRPVDDVKIIKAYPSG  158
         **:       ***:*.:**.: .*  : . :         :      * ..
```

Exposed

Buried

In binding site

Supplementary Figure 8 (continued): Bioinformatic analysis of Cyps and PROTAC designs to understand PROTAC selectivity

a TWH106 is shown docked in CypA (PDB 1CWA) as a representative example for Cyps. Residues E81 and K82 (highlighted in magenta) vary across Cyps as indicated in the table. **b** The two PROTAC designs based on TWH106 extend from methyl groups positioned in the left side of the binding site. In contrast, JW4-10, a CsA-based PROTAC, extends towards the right of the binding site. These directions may influence different biological profiles of the PROTACs. **c** Domain structure of different Cyps and sequence identity to CypA. Differential C- and N-termini may impact Cyp accessibility for degradation **d** Main reported localisation for different Cyps, which may affect their accessibility for compound binding and proteasomal degradation[6, 7]. **e** Differences in surface-exposed residues of Cyps are highlighted on CypA as a model structure. Residues are coloured according to sequence similarity for each Cyp, using the default colour scheme of the MOE software derived from the BLOSUM62 scoring matrix. These surface differences may influence ternary complex formation **f** Lysine positions in Cyps are highlighted from a sequence alignment of the main domain. The solvent exposure is indicated for CypA lysines by colour. Different lysine substitution patterns may affect ubiquitination efficiency.

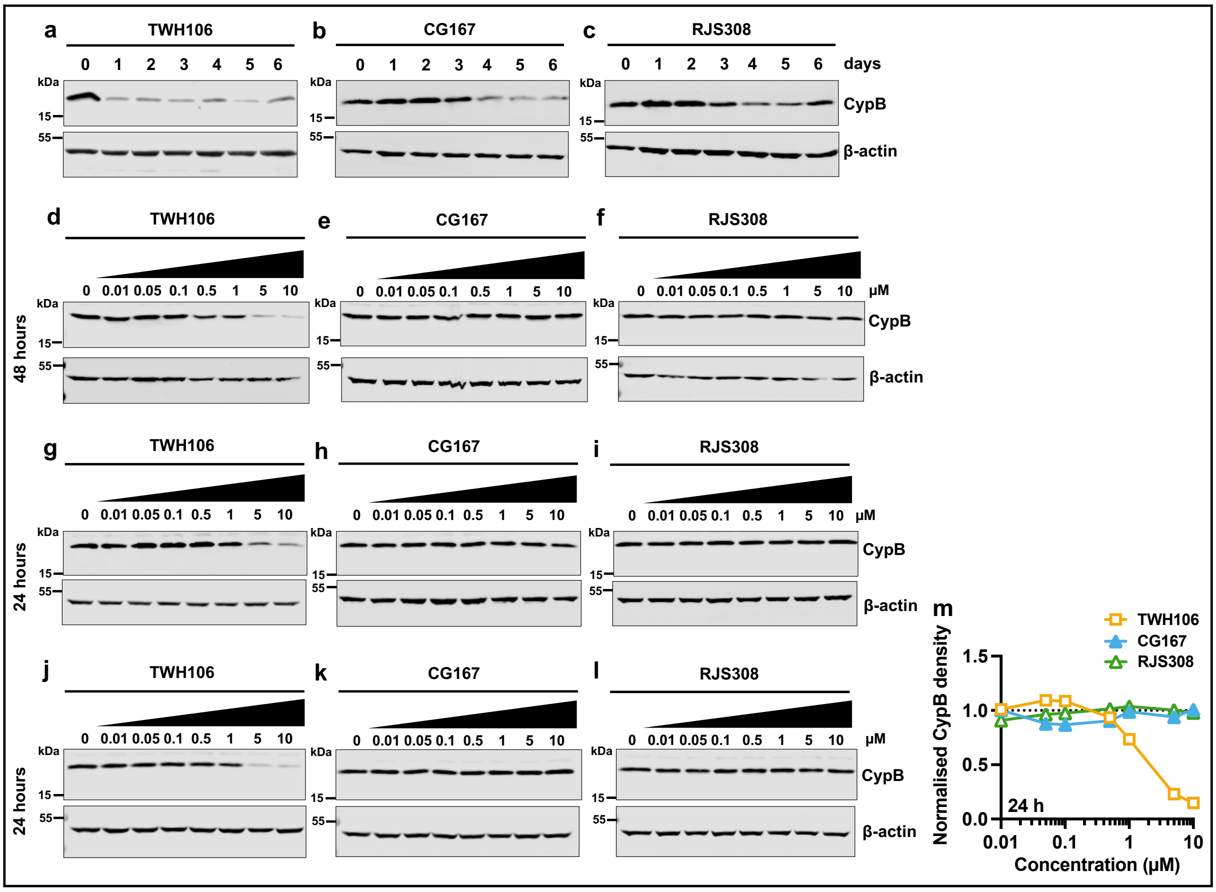

Supplementary Figure 9: TWH106 depletes CypB in a dose-dependent manner
a-l Immunoblots detecting CypB, or β-actin as loading control, in Jurkat cells treated with TWH106, CG167 or RJS308 at (**a-c**) 5 μM for 6 days or (**d-f**) 0.01 – 10 μM for 48 h or (**g-l**) 0.01 – 10 μM for 24 h**. m** CypB densities from (**g-l**) adjusted for loading by reference to β-actin densities, mean (*n* = 2 independent experiments). Experiments (**a-f**) were carried out independently to those in Fig. 5 and were used to calculate CypB densities (Fig. 5h, l).

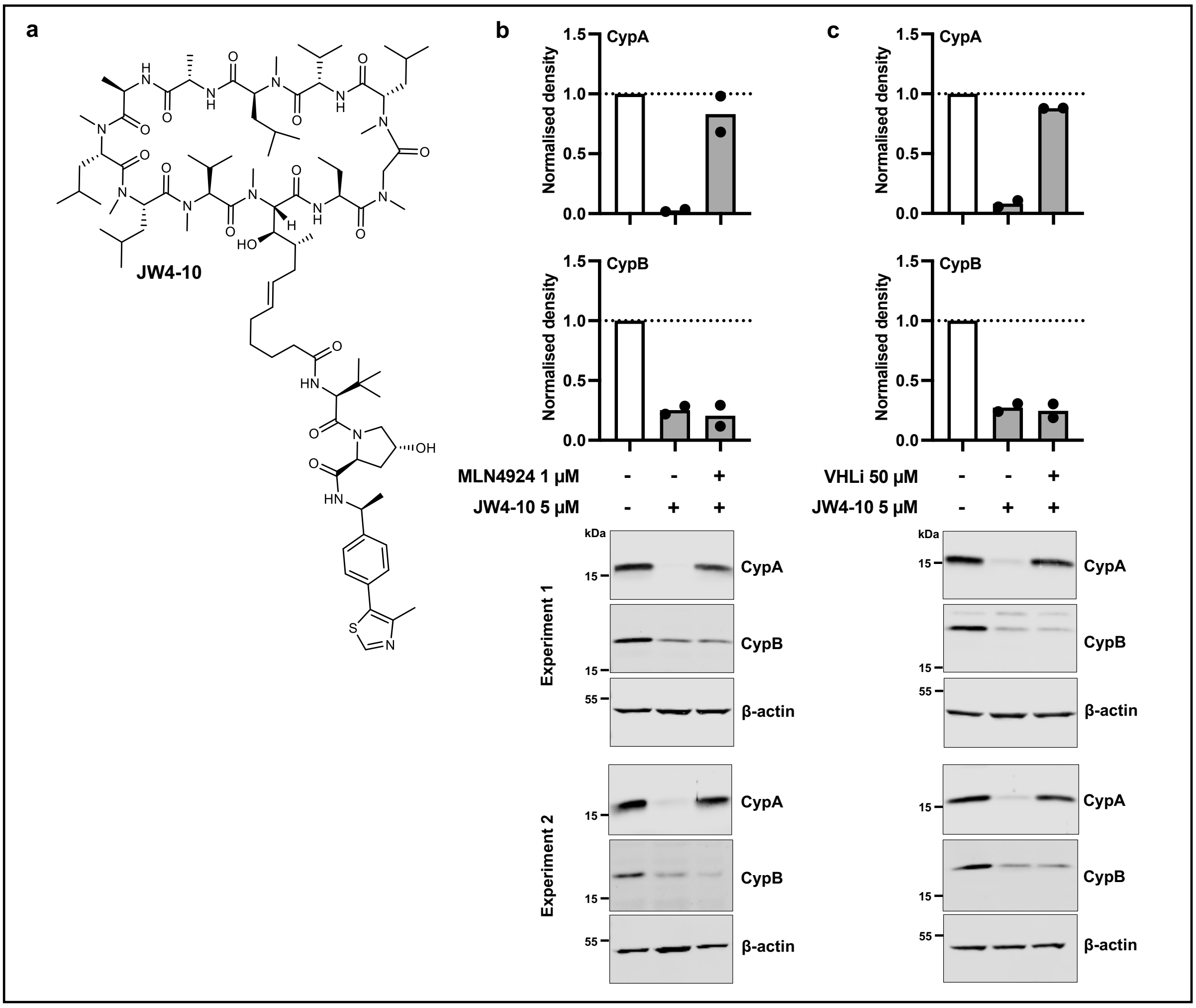

Supplementary Figure 10: Characterisation of CsA-based PROTAC JW4-10

a Chemical structure of JW4-10. **b-c** Immunoblots detecting CypA, CypB, or β-actin as loading control, in Jurkat cells pretreated with (**b**) 1 µM NEDD8-activating enzyme inhibitor MLN4924 for 6 h or (**c**) 50 µM VHL inhibitor VH298 (VHLi) for 2 h, followed by PROTAC JW4-10 for 48 h at 5 µM. CypA and CypB densities (top) adjusted for loading by reference to β-actin densities, mean (*n* = 2 independent experiments).

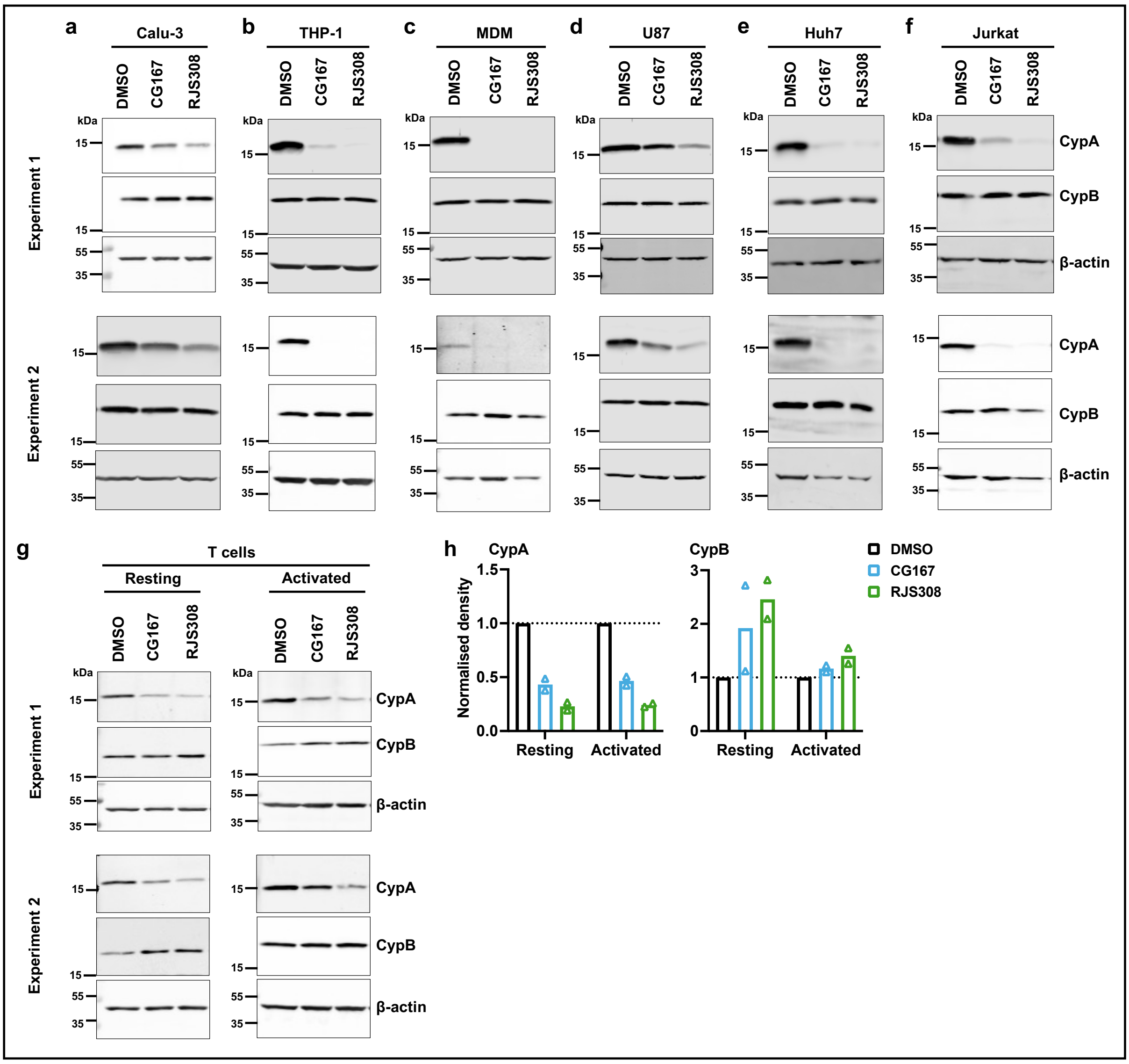

Supplementary Figure 11: Degradation of cyclophilins A and B in different cell types
a-g Immunoblots detecting CypA, CypB, or β-actin as loading control, in (**a**) Calu-3 cells, (**b**) THP-1 cells, (**c**) monocyte-derived macrophages (MDMs), (**d**) U87 cells, (**e**) Huh7 cells, (**f**) Jurkat cells, or (**g**) resting or activated CD4+ T cells treated with 5 μM CG167 or RJS308 for 48 h. Experiments (**a-f**) were used to calculate CypA and CypB densities in Fig. 5m-n. **h** CypA (left) and CypB (right) densities from (**g**) adjusted for loading by reference to β-actin densities, mean (*n* = 2 independent experiments). Different donors were used for independent experiments with T cells (**g-h**) and MDMs (**c**).

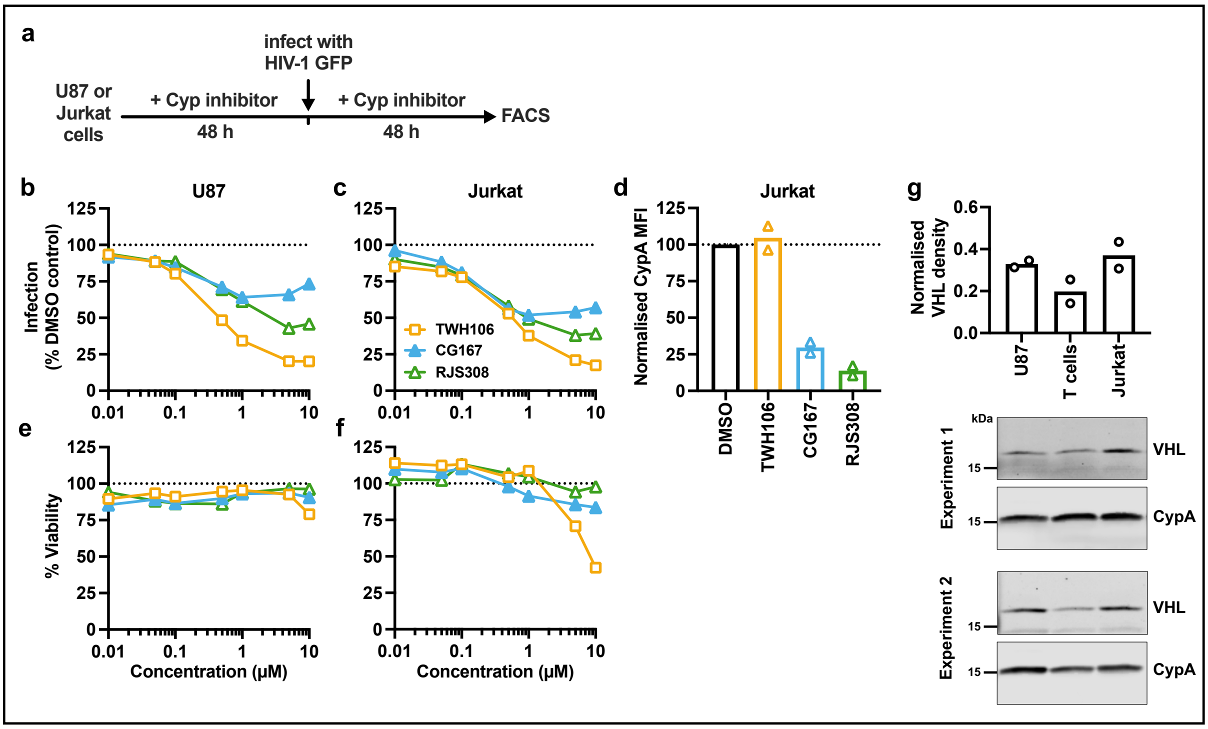

Supplementary Figure 12: PROTACs are antiviral against single-round HIV-1 infection
a Experimental design for (**b-f**): U87 or Jurkat cells pretreated for 48 h with 0.01 – 10 μM TWH106 (orange), CG167 (blue), or RJS308 (green) were infected with (**b**) HIV-1 GFP lentiviral vector or (**c**) HIV-1 LAI ΔEnv GFP and fresh Cyp inhibitor added. Infection levels (GFP+) of (**b**) U87 cells or (**c**) Jurkat cells 48 h after infection, normalised to DMSO, mean (*n* = 2 independent experiments performed in triplicate). **d** CypA mean fluorescence intensity (MFI) of Jurkat cells from (**b**) treated with 5 μM Cyp inhibitor for 96 h, normalised to DMSO, mean (*n* = 2 independent experiments performed in triplicate). **e-f** Viability of (**e**) U87 cells or (**f**) Jurkat cells treated with Cyp inhibitors as in (**b-c**) measured by MTT assay, normalised to DMSO, mean (*n* = 2 independent experiments performed in triplicate). Gating strategies are shown in Supplementary Fig. 15d. **g** Immunoblots detecting VHL, or CypA as loading control, in U87 cells, primary T cells and Jurkat cells. VHL densities (top) adjusted for loading by reference to CypA densities, mean (*n* = 2 independent experiments). Different donors were used for independent experiments with T cells.

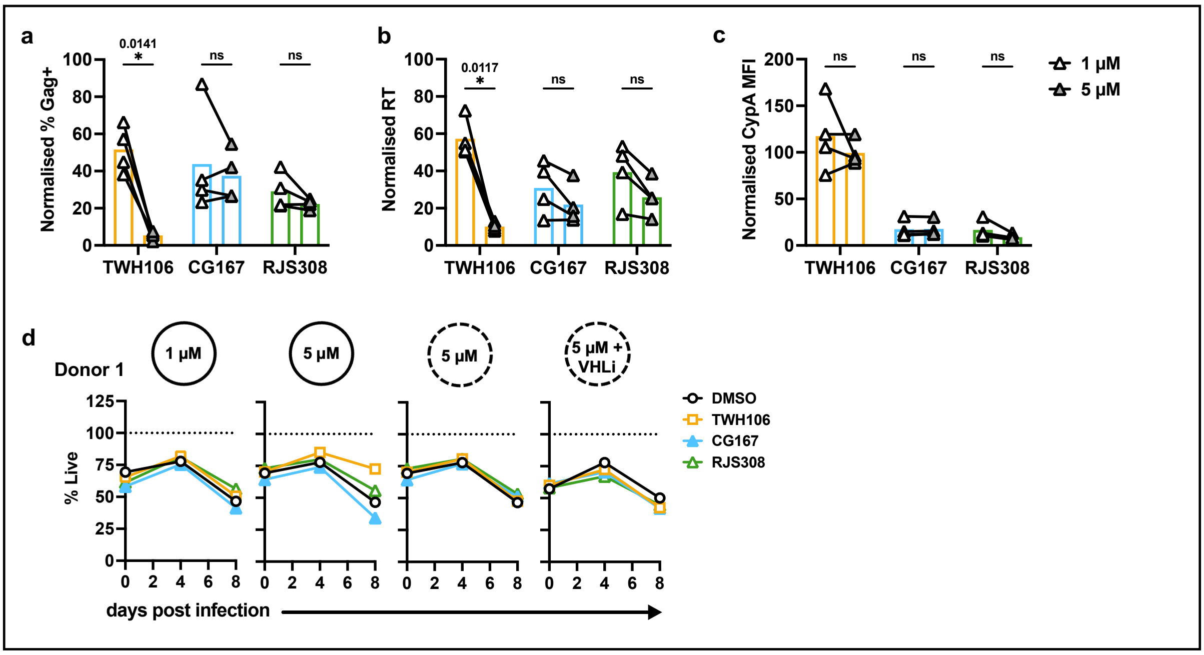

Supplementary Figure 13: Further characterisation of TWH106 and Cyp-PROTAC anti-HIV-1 activity

a-c HIV-1 spreading infection data (Fig. 6n, o, r, s, v, w), plotted to show comparison between 1 μM and 5 μM conditions, all data normalised to DMSO, mean ± SD (*n* = 4 donors), (**a**) % Gag+ cells at 4 days post infection (dpi), (**b**) virus levels in supernatant at 4 dpi measured by SG-PERT, (**c**) CypA mean fluorescence intensity (MFI) at time of infection (0 dpi). Statistical comparison using paired t-tests with Holm-Šídák's multiple comparisons test, * (P ≤ 0.05), ** (P ≤ 0.01), *** (P ≤ 0.001), **** (P ≤ 0.0001), P values shown. **d** Donor 1 % Live cells for indicated treatment conditions at indicated dpi (% of all cells)**,** mean (*n* = 1 independent experiment performed in duplicate). Gating strategies are shown in Supplementary Fig. 15a-c.

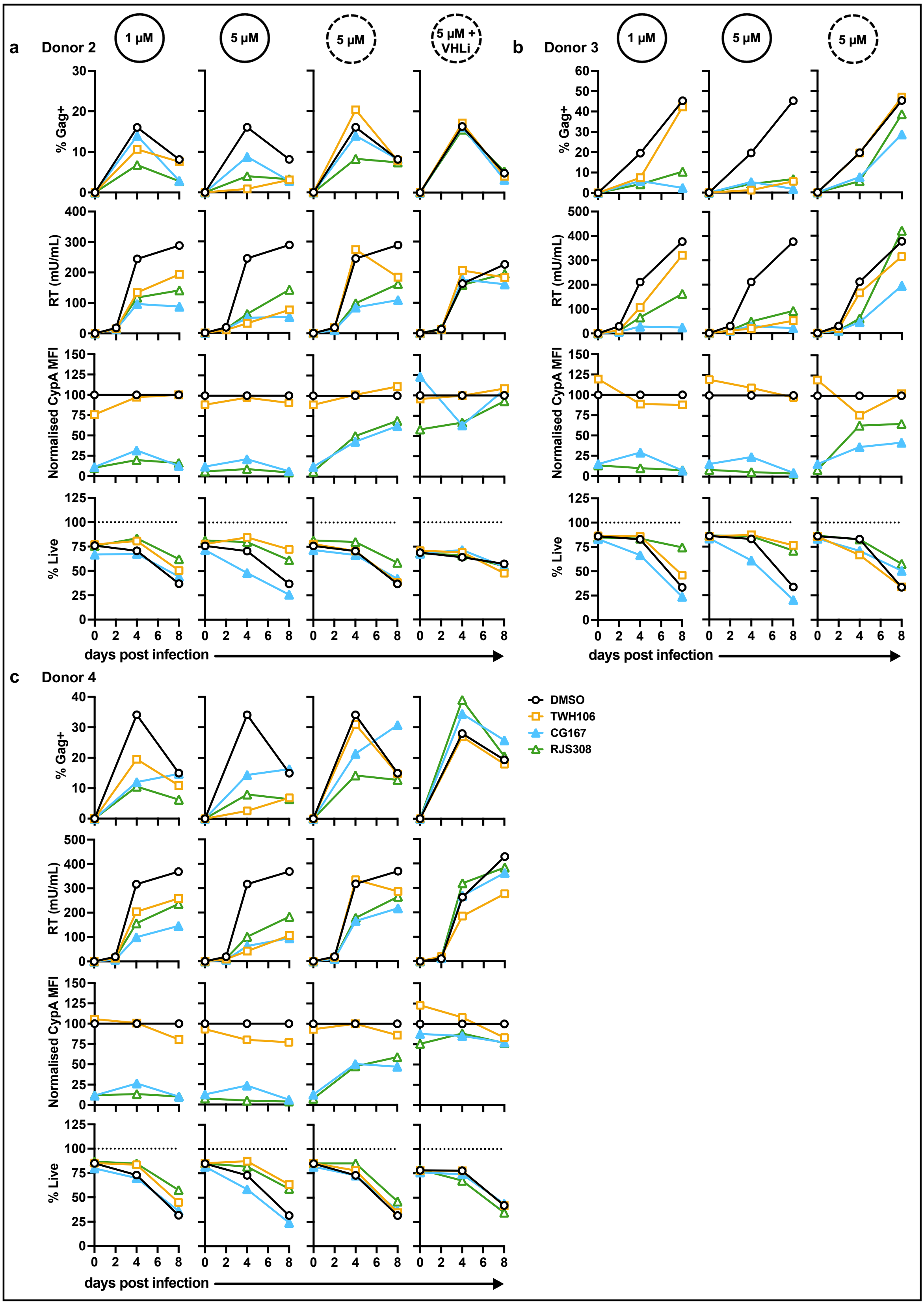

Supplementary Figure 14: Anti-HIV-1 activity: data from additional donors

a-c HIV-1 spreading infection timecourse data from 3 donors, additional to the representative donor presented in Fig. 6b-m. Measurements of % Gag+ cells, virus levels in supernatant measured by SG-PERT, CypA mean fluorescence intensity (MFI) normalised to DMSO, and % Live cells at indicated days post infection, Donor 2 (**a**), Donor 3 (**b**), Donor 4 (**c**), mean (*n* = 1 independent experiment performed in duplicate). Gating strategies are shown in Supplementary Fig. 15a-c.

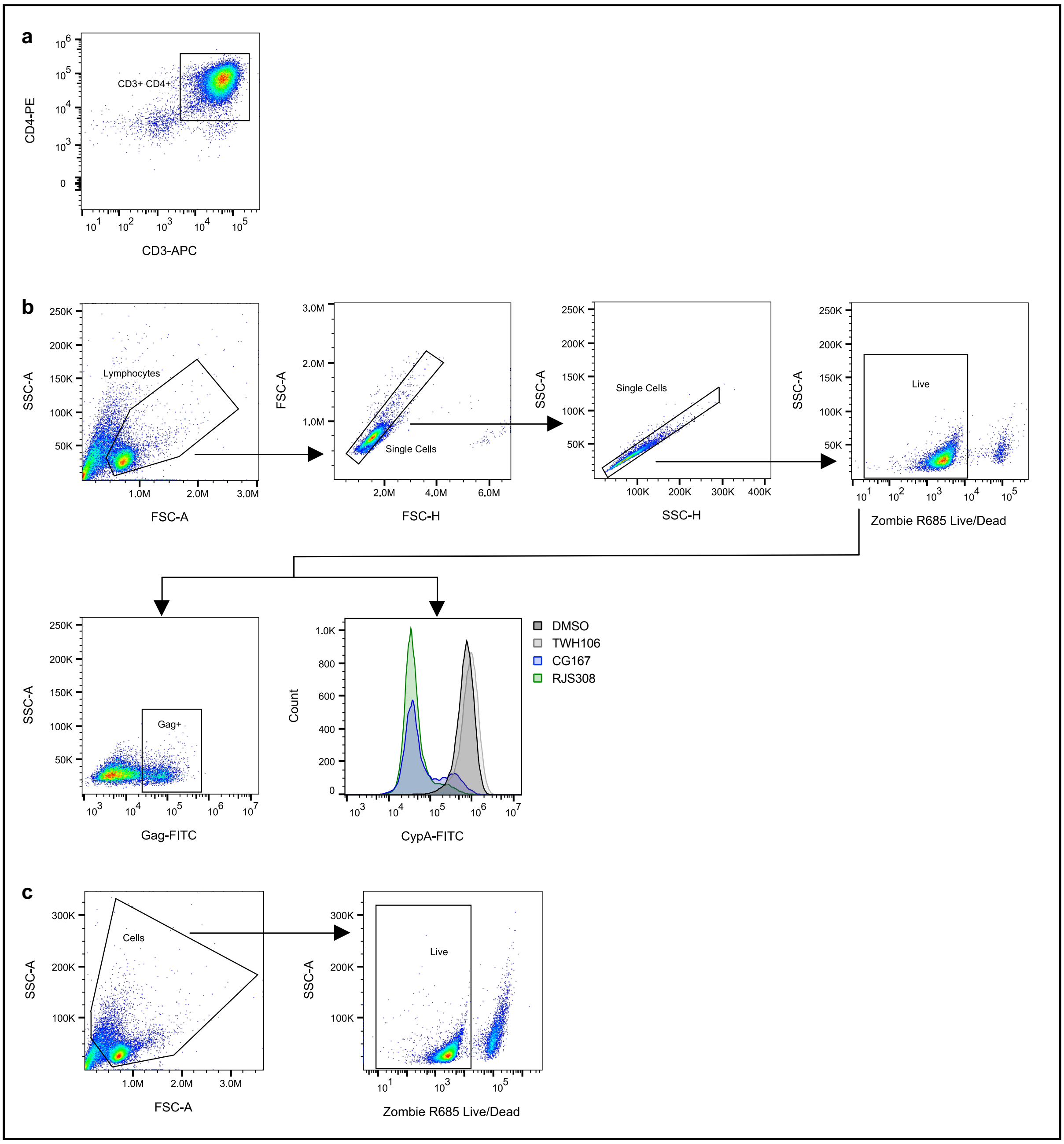

Supplementary Figure 15: Flow cytometry gating strategies

(continued next page)

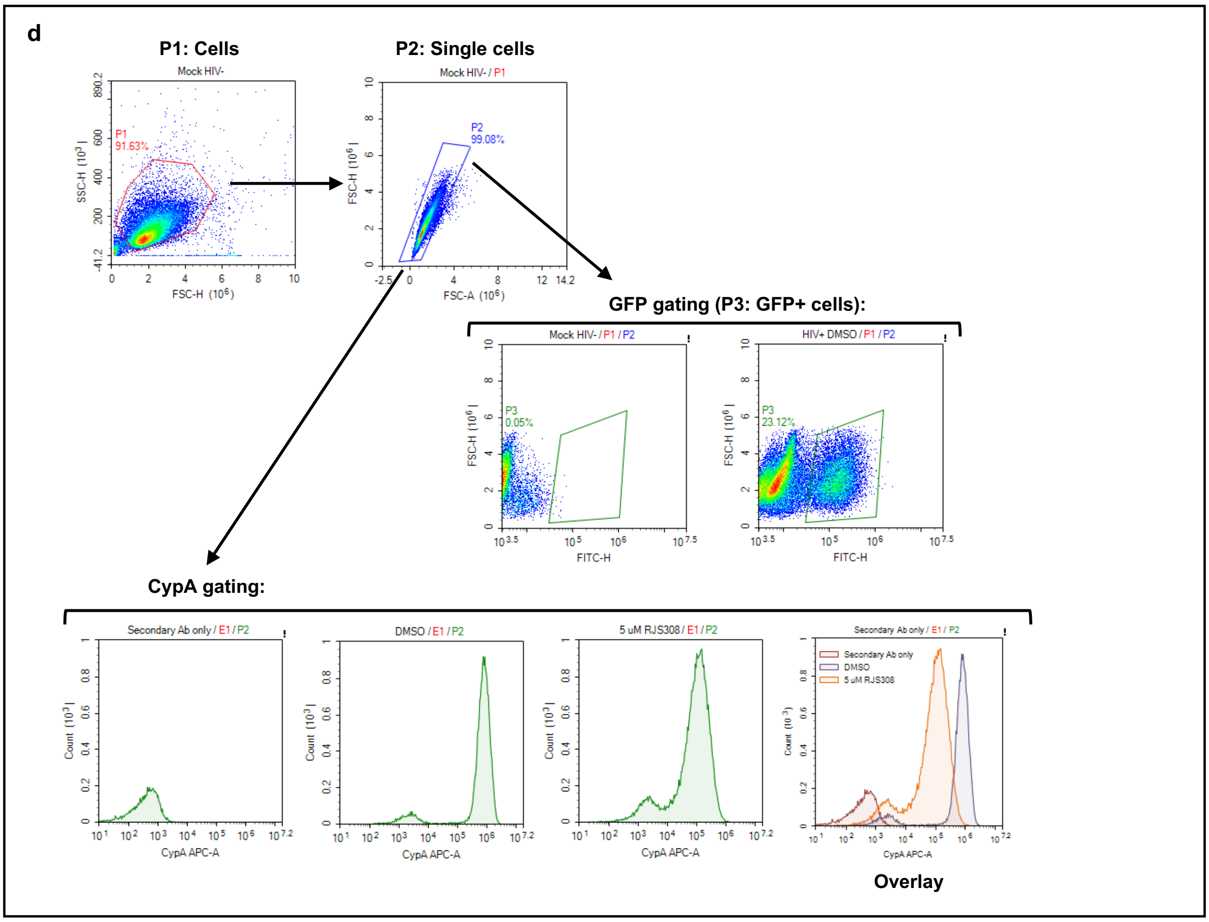

Supplementary Figure 15 (continued): Flow cytometry gating strategies
a CD3 and CD4 staining of CD4+ T cells to check for purity after isolation. **b-c** Gating of primary T cells (Fig. 6, Supplementary Fig. 13, Supplementary Fig. 14) for analysis of (**b**) Gag positivity or CypA mean fluorescence intensity (MFI) or (**c**) analysis of live cells. **d** Gating of U87 or Jurkat cells (Supplementary Fig. 12b-d) for analysis of GFP positivity or CypA MFI.

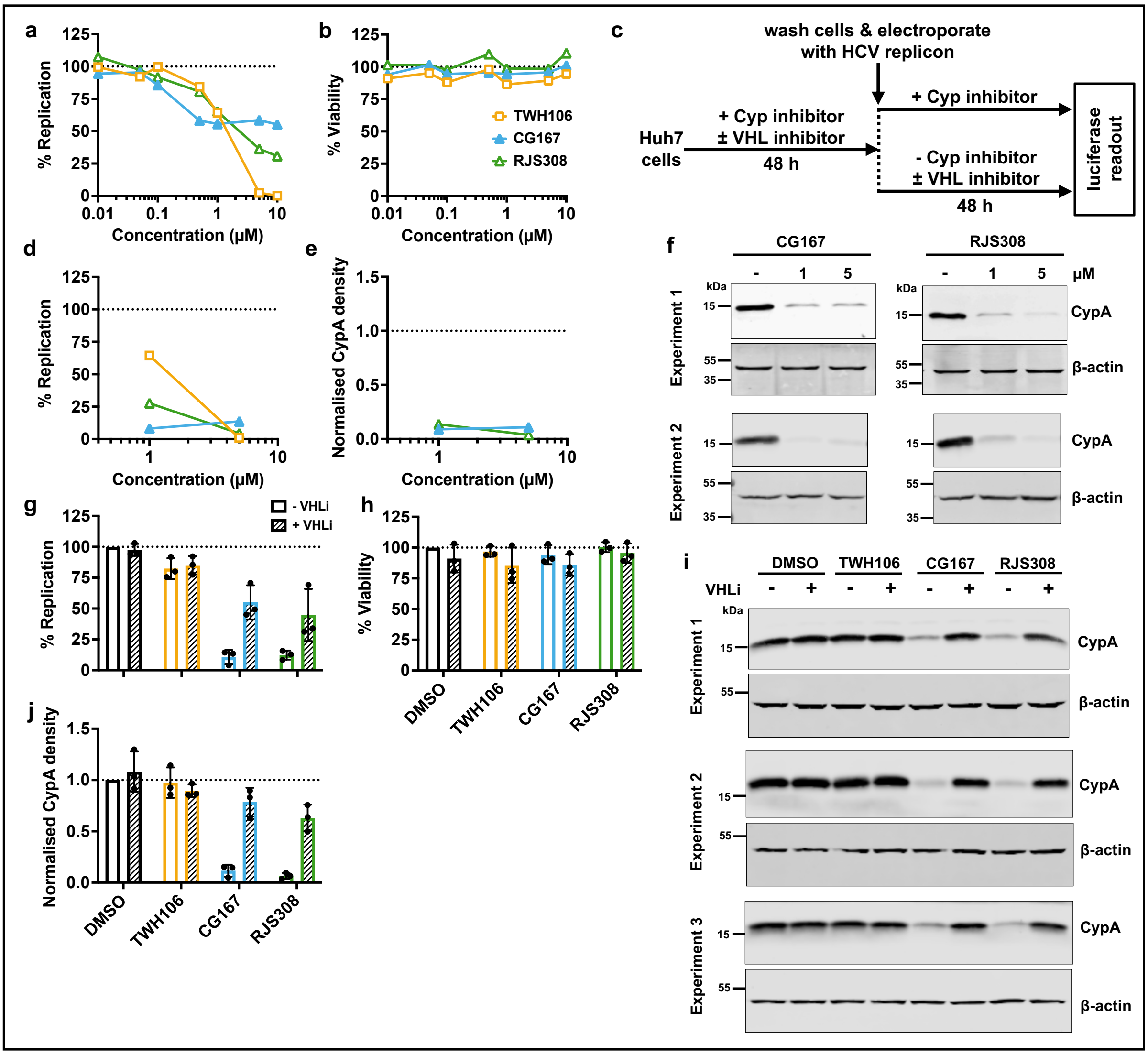

Supplementary Figure 16: Cyp-PROTACs are antiviral against HCV replicon

a Replication levels (luciferase readout) in Huh7 cells electroporated with HCV JFH-1 replicon RNA, plated and washed after 4 h, then treated with 0.01 – 10 µM TWH106 (orange), CG167 (blue) or RJS308 (green). Readout 48 h post electroporation (hpe), normalised to DMSO, mean (*n* = 2 independent experiments performed in triplicate). **b** Viability of cells treated with Cyp inhibitors as in (**a**), measured by MTT assay, normalised to DMSO, mean (*n* = 1 independent experiment performed in triplicate). **c** Experimental design for (**d-j**): Huh7 cells were pretreated for 48 h with TWH106, CG167 or RJS308 at 1 µM, 5 µM, or 5 µM and 50 µM VHL inhibitor VH298 (VHLi), then washed and electroporated with HCV JFH-1 replicon RNA, plated and washed after 4 h. Cyp inhibitors were then readded (**d-f**) or not readded (**g-j**). **d** Replication levels (luciferase readout) 48 hpe, normalised to DMSO, mean (*n* = 2 independent experiments performed in triplicate). **e-f** Immunoblots (**f**) detecting CypA, or β-actin as loading control, in Huh7 cells treated with 1 µM or 5 µM CG167 or RJS308 for 48 h. CypA densities (**e**) adjusted for loading by reference to β-actin densities, mean (*n* = 2 independent experiments performed in triplicate). **g** Replication levels (luciferase readout) 48 hpe, normalised to DMSO, mean ± SD (*n* = 3 independent experiments performed in triplicate). **h** Viability of cells treated with Cyp inhibitors ± VHLi for 48 h as for (**g**), measured by MTT assay, normalised to DMSO, mean ± SD (*n* = 3 independent experiments performed in triplicate). **i-j** Immunoblots (**i**) detecting CypA, or β-actin as loading control, at the time of electroporation in (**g**). CypA densities (**j**) relative to β-actin, mean ± SD (*n* = 3 independent experiments).

Synthesis and characterisation of compounds

General

All commercially available solvents and reagents were used without further treatment as received unless otherwise noted.

Nuclear Magnetic resonance (NMR) spectra were measured on Avance III 400/600 MHz or Avance Neo 700/500 MHz Bruker spectrometers. Chemical shifts are reported in parts per million (ppm) with the residual protic solvent resonance as the internal standard ($CDCl_3$: 7.27 (^1H), 77.0 (^{13}C) CD_3OD: 4.87 (^1H), 49.0 (^{13}C), Acetone-d_6: 2.04 (^1H), 29.80 (^{13}C), DMSO-d_6: 2.50 (^1H), 39.50 (^{13}C)). Data is reported as follows: chemical shift, multiplicity (s = singlet, d = doublet, t = triplet, q = quartet, m = multiplet), coupling constant in Hz, integration. All NMR spectra were recorded at 298 K, unless reported otherwise. Raw data was processed with Mestrenova (MestreLab, 14.3.1).

High resolution mass spectra were measured by electrospray ionisation (ESI) on a LCT Premier XE Q-TOF mass spectrometer or by atmospheric solids analysis probe - heated electrospray ionisation (ASAP-HESI) on a Q exactive Plus mass spectrometer. Measurements were performed by the UCL mass spectrometry service.

LC-MS data was obtained using an Agilent single quadrupole mass spectrometer using ESI and an analytical C18 ZORBAX SB column (2.1 x 50 mm, 1.8 µm, 0.7 mL/min) or C4 Phenomenex Gemini column (3.6 x 50 mm, 5 µm, 1 mL/min). The gradient used was 10-95% MeCN/H_2O (C18) or 20-95% MeCN/H_2O (C4) with 0.1% formic acid modifier.

Flash column chromatography was performed using a Biotage Isolera I or IV using commercially available cartridges from Biotage.

The VHL ligand 2 (1948273-03-7) was bought from Medchem Express as the hydrochloride salt.

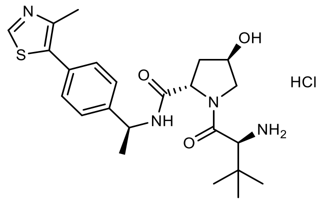

VHL ligand 2

7-bromo-N-methoxy-N-methylquinoline-2-carboxamide (s5)

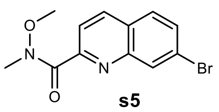

s5

7-bromoquinoline-2-carboxylic acid (2.97 g, 11.8 mmol, 1.00 eq), HATU (5.87 g, 15.4 mmol, 1.31 eq) and *i*Pr-NEt$_2$ (5.0 mL, 28.4 mmol, 2.40 eq) were dissolved in dry DMF (35 mL), producing a brown precipitate within minutes. N,O-dimethyl hydroxylamine hydrochloride (1.39 g, 14.3 mmol, 1.21 eq) was added in one portion and the now clear brown solution was stirred for 5h, showing completion by LC-MS. The reaction was diluted with EtOAc and washed with sat. aq. LiCl (2x). The aqueous layer was extracted with EtOAc (1x) and the combined organic layers were washed with $NaHCO_3$ (1x) and brine (1x). The organic layer was dried over $MgSO_4$ and concentrated under reduced pressured to yield a brown oil (9.01 g). This oil was purified by flash chromatography (Silica, 20-40% EtOAc/cHex) to afford Weinreb amide **s5** as a yellow oil which solidified upon standing (3.35 g, 11.4 mmol, 96% yield).

R_f = 0.18 (20% EtOAc/cHex).

^{1}H NMR (600 MHz, $CDCl_3$) δ 8.34 (s, 1H), 8.24 (d, *J* = 8.4 Hz, 1H), 7.75 (d, *J* = 8.6 Hz, 1H), 7.70 (dd, *J* = 8.7, 1.9 Hz), 7.68 (br s, 1H), 3.79 (br s, 3H), 3.44 (br s, 3H).

^{13}C NMR (151 MHz, $CDCl_3$) δ 168.53, 153.80, 146.66, 137.36, 131.55, 131.39, 128.90, 126.88, 124.64, 120.20, 61.93, 32.68. Note: Signals greatly reduced due to rotational isomers

HRMS (ESI+): calcd. for $[C_{12}H_{11}BrN_2O_2+H]^+$ (MH^+) 295.0077; found 295.0078.

LC trace (254 nM)

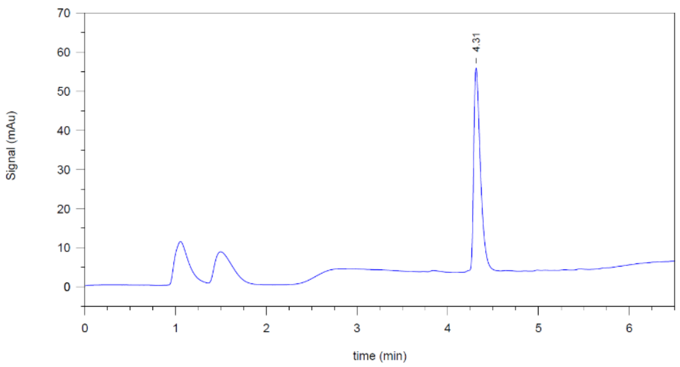

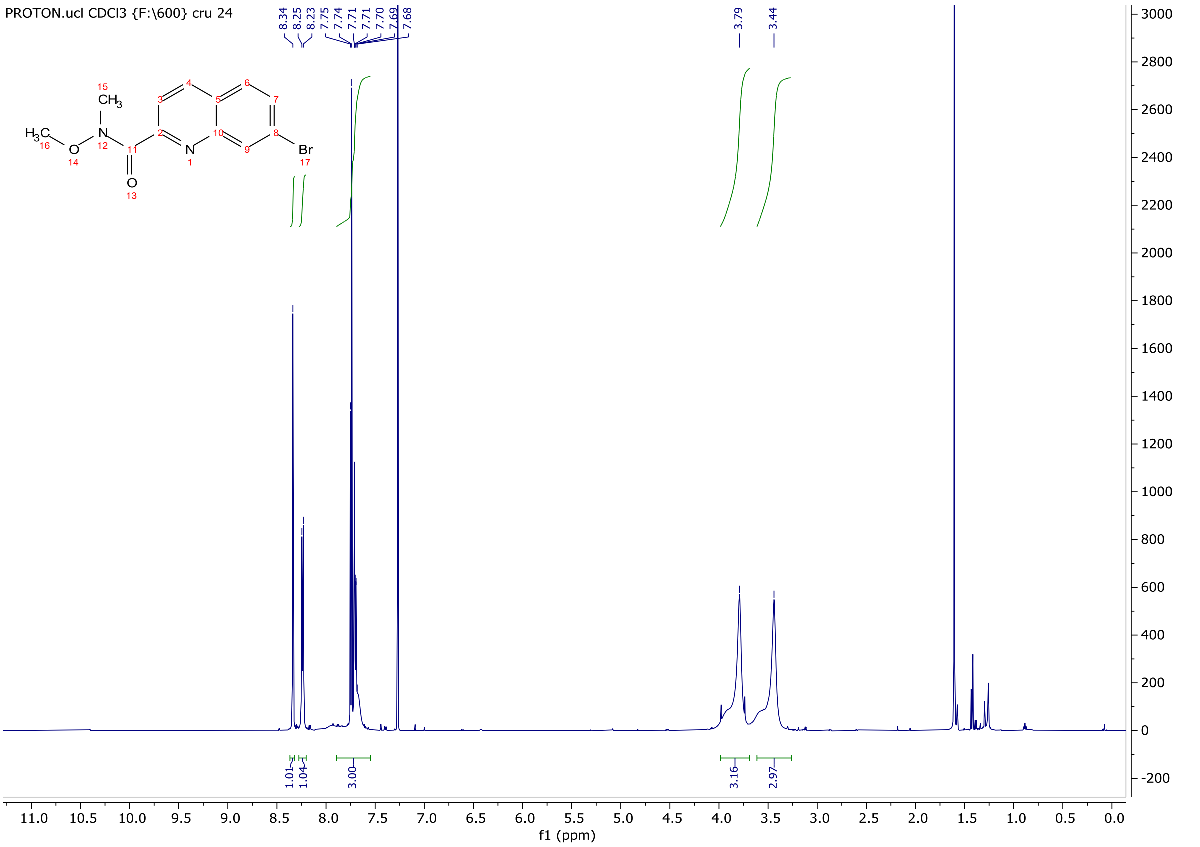
PROTON.ucl CDCl3 {F:\600} cru 24
8.34
8.25
8.23
7.75
7.74
7.71
7.71
7.70
7.69
7.68
3.79
3.44
1.01
1.04
3.00
3.16
2.97
f1 (ppm)

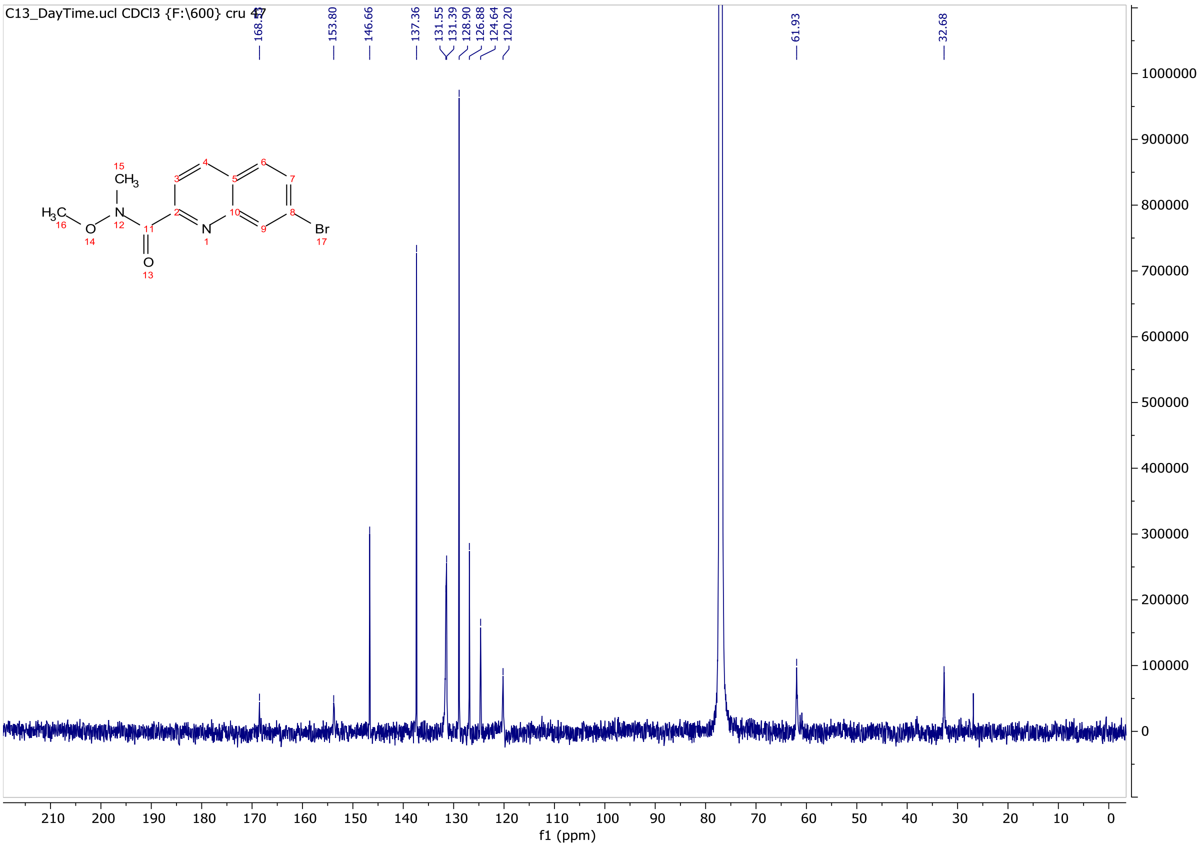
C13_DayTime.ucl CDCl3 {F:\600} cru 47
168.47
153.80
146.66
137.36
131.55
131.39
128.90
126.88
124.64
120.20
61.93
32.68
f1 (ppm)

1-(7-bromoquinolin-2-yl)ethan-1-one (s6)

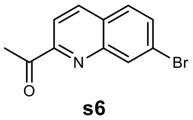

s6

7-bromo-N-methoxy-N-methylquinoline-2-carboxamide **s5** (655 mg, 2.22 mmol, 1.00 eq), was dissolved in dry THF (30 mL) at -78°C under N_2. A solution of MeMgBr (3M in Et_2O, 1.2 mL, 3.60 mmol, 1.62 eq) was added dropwise for 20 min. The orange solution was stirred for 15 h upon warming up to 25 °C. The reaction was quenched with sat. aq. NH_4Cl (5 mL), then diluted with H_2O and EtOAc. The layers were separated, and the aqueous layer was extracted with EtOAc (1x). The combined organic layers were washed with brine (1x), dried over $MgSO_4$, and concentrated under reduced pressure to afford pure methyl ketone **s6** (537 mg, 2.15 mmol, 97% yield) as a light brown solid.

^{1}H NMR (600 MHz, $CDCl_3$) δ 8.42 (s, 1H), 8.26 (d, *J* = 8.4 Hz, 1H), 8.15 (d, *J* = 8.4 Hz, 1H), 7.76 (d, *J* = 8.7 Hz, 1H), 7.74 (dt, *J* = 8.7, 1.4 Hz, 1H), 2.86 (s, 3H).

^{13}C NMR (151 MHz, $CDCl_3$) δ 200.30, 153.80, 147.79, 136.88, 132.76, 132.01, 128.83, 128.11, 124.10, 118.29, 25.56.

HRMS (ESI+): calcd. for $[C_{11}H_8BrNO+H]^+$ (MH^+) 249.9864; found 249.9862.

LC trace (254 nM)

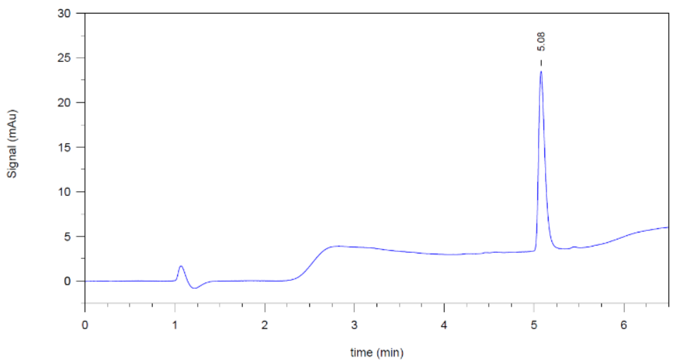

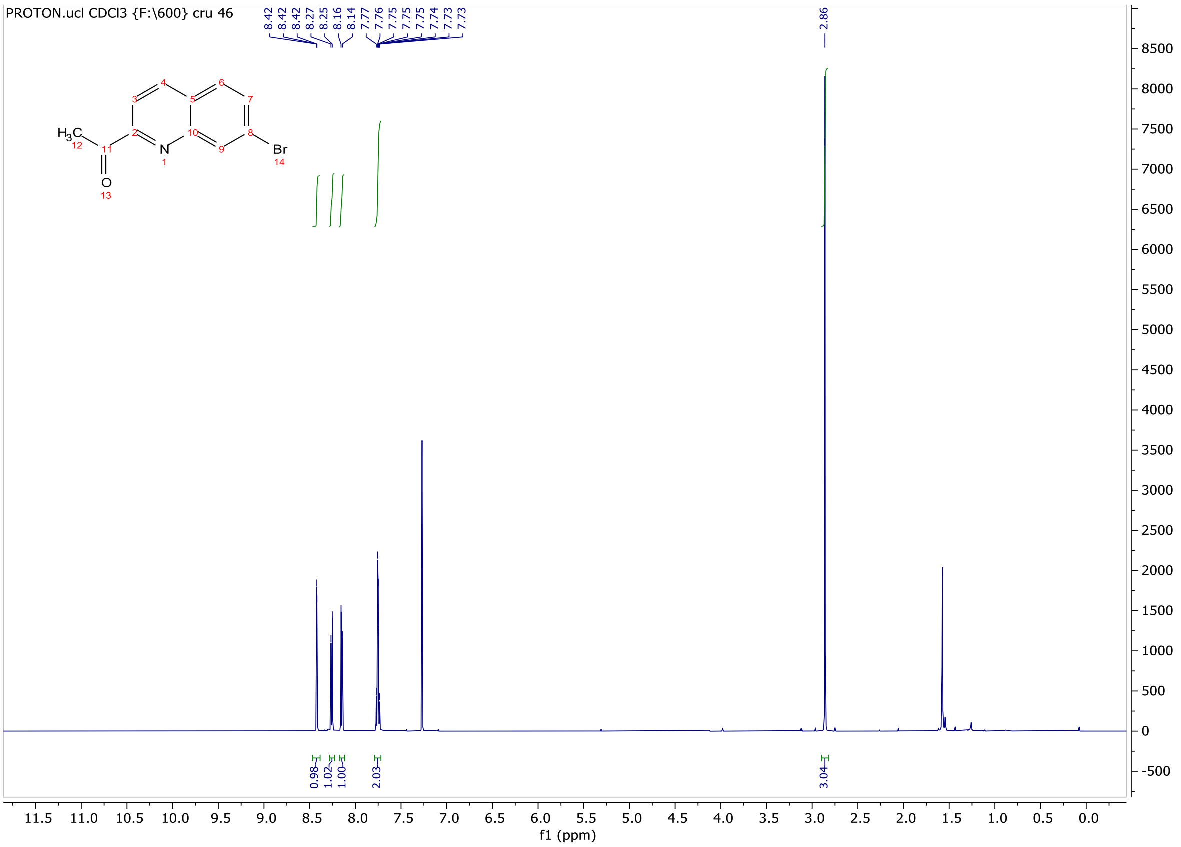
PROTON.ucl CDCl3 {F:\600} cru 46
8.42
8.42
8.42
8.27
8.25
8.16
8.14
7.77
7.76
7.75
7.75
7.75
7.74
7.73
7.73
2.86
H3C
12
11
O
13
N
1
2
3
4
5
6
7
8
9
10
Br
14
0.98
1.02
1.00
2.03
3.04
f1 (ppm)

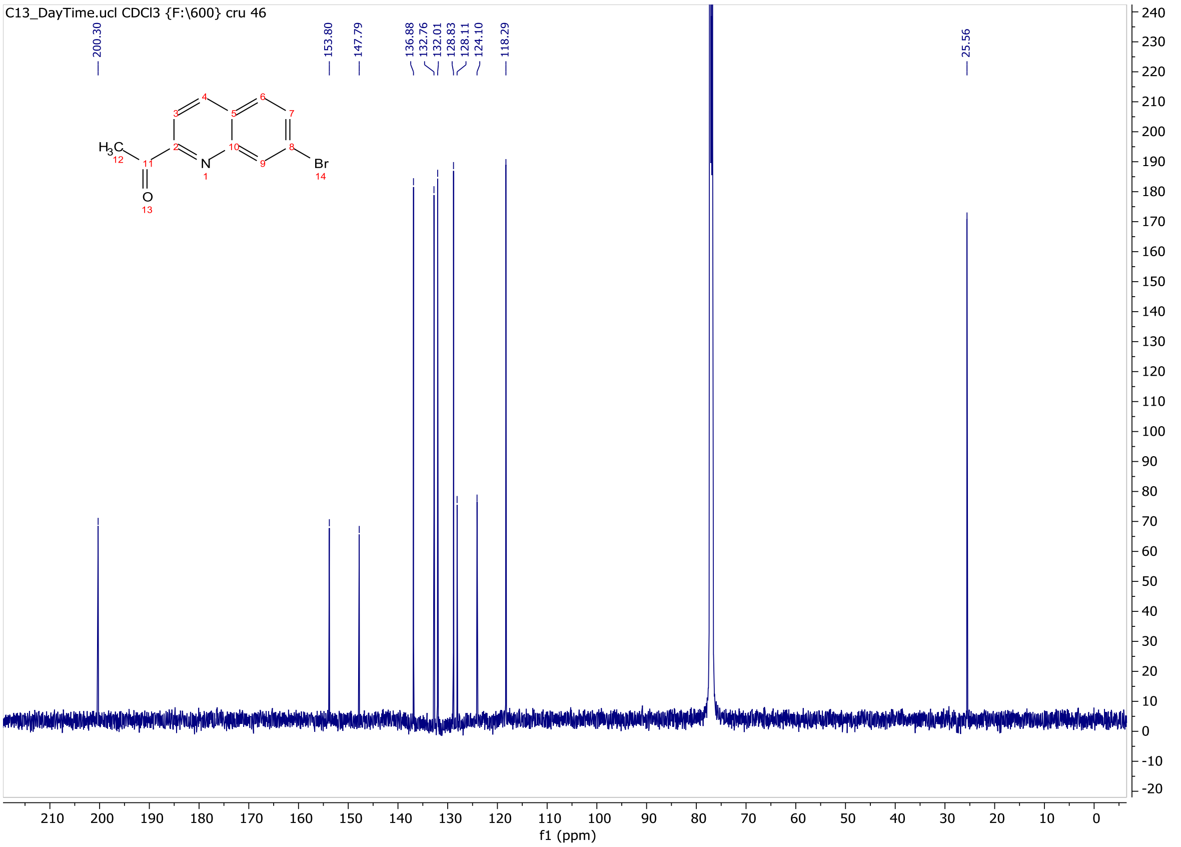
C13_DayTime.ucl CDCl3 {F:\600} cru 46
200.30
153.80
147.79
136.88
132.76
132.01
128.83
128.11
124.10
118.29
25.56
H3C
12
11
O
13
N
1
2
3
4
5
6
7
8
9
10
Br
14
f1 (ppm)

1-(7-bromoquinolin-2-yl)-6-(trimethylsilyl)hex-5-yn-1-one (s7)

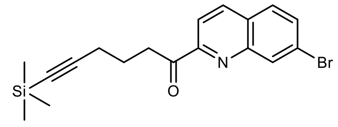

s7

For 16h prior to the reaction, several grams of Mg(0) turnings were stirred under N_2 for activation. Trimethylsilylpentynyl chloride (4.08 g, 23.0 mmol, 1.00 eq) in dry THF (20 mL) was added to activated Mg(0) turnings (1.15 g, 47.0 mmol, 2.00 eq) under N_2. The black mixture was slowly heated to 60°C while dibromoethane (0.44 mL, 5.10 mmol, 0.50 eq) was added dropwise for 10 min. Gas evolution was observed at around 50°C, and the reaction was stirred for further 3 h at 60°C. Titration with salicylphenylhydrazone[8] indicated 0.5 M Grignard concentration.

A solution of Weinreb amide **s5** (2.08 g, 7.05 mmol, 1.00 eq) in dry THF (45 mL) under N_2 was cooled down to -78°C. The prepared Grignard solution was added in several portions over 2h (4 x 5.5 mL, ca 11 mmol) with a syringe equipped with a cotton filter. The reaction was let to warm up to 25°C for 45 min, after which completion was observed by TLC. The reaction was quenched with sat aq. NH_4Cl (5 mL). The solution was diluted with EtOAc and H_2O, and the layers were separated. The aqueous layer was extracted with EtOAc (2x). The combined organic layers were dried over $MgSO_4$ and concentrated under reduced pressure to yield a crude orange oil (3.08 g). Purification by flash chromatography (silica, 0-3% EtOAc/cHex) afforded alkyne ketone **s7** (734 mg, 1.96 mmol, 28% yield) as a white solid.

Note: The low yield is due to a demethylation side reaction of the Weinreb amide[9]. This product is best used within 24h as it degrades slowly to a coloured specie which inhibits subsequent catalysis.

R_f = 0.28 in 1% EtOAc/cHex.

^{1}H NMR (600 MHz, $CDCl_3$) δ 8.41 (s, 1H); 8.26 (d, J = 8.5 Hz, 1H); 8.14 (d, J = 8.5 Hz, 1H); 7.79 -7.71 (m, 2H); 3.50 (t, J = 7.2 Hz, 2H); 2.42 (t, J = 7.2 Hz, 2H); 2.03 (p, J = 7.2 Hz, 2H); 0.15 (s, 9H).

^{13}C NMR (151 MHz, $CDCl_3$) δ 201.57, 153.53, 147.75, 136.88, 132.75, 131.97, 128.83, 128.14, 124.08, 118.43, 106.68, 85.24, 36.36, 23.02, 19.50, 0.13.

HRMS (ESI+): calcd. for $[C_{18}H_{20}BrNOSi+H]^+$ (MH^+) 374.0570; found 374.0574.

LC trace (254 nM)

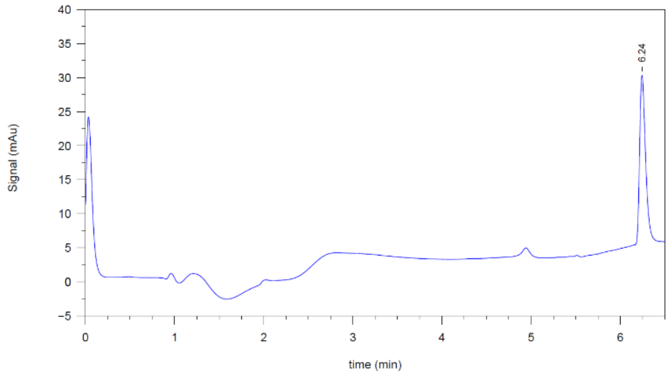

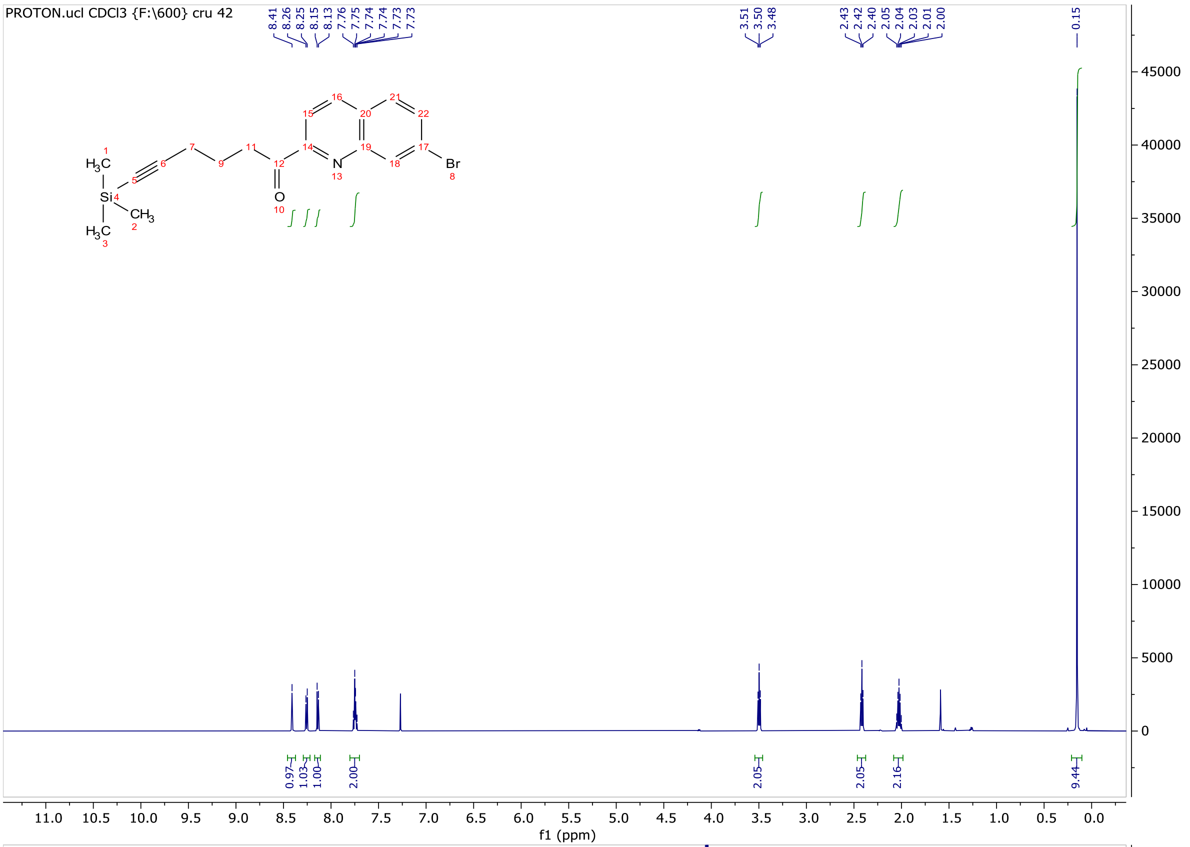
PROTON.ucl CDCl3 {F:\600} cru 42
8.41
8.26
8.25
8.15
8.13
7.76
7.75
7.74
7.74
7.73
7.73
3.51
3.50
3.48
2.43
2.42
2.40
2.05
2.04
2.03
2.01
2.00
0.15
0.97
1.03
1.00
2.00
2.05
2.05
2.16
9.44
Br
O
N
Si
f1 (ppm)
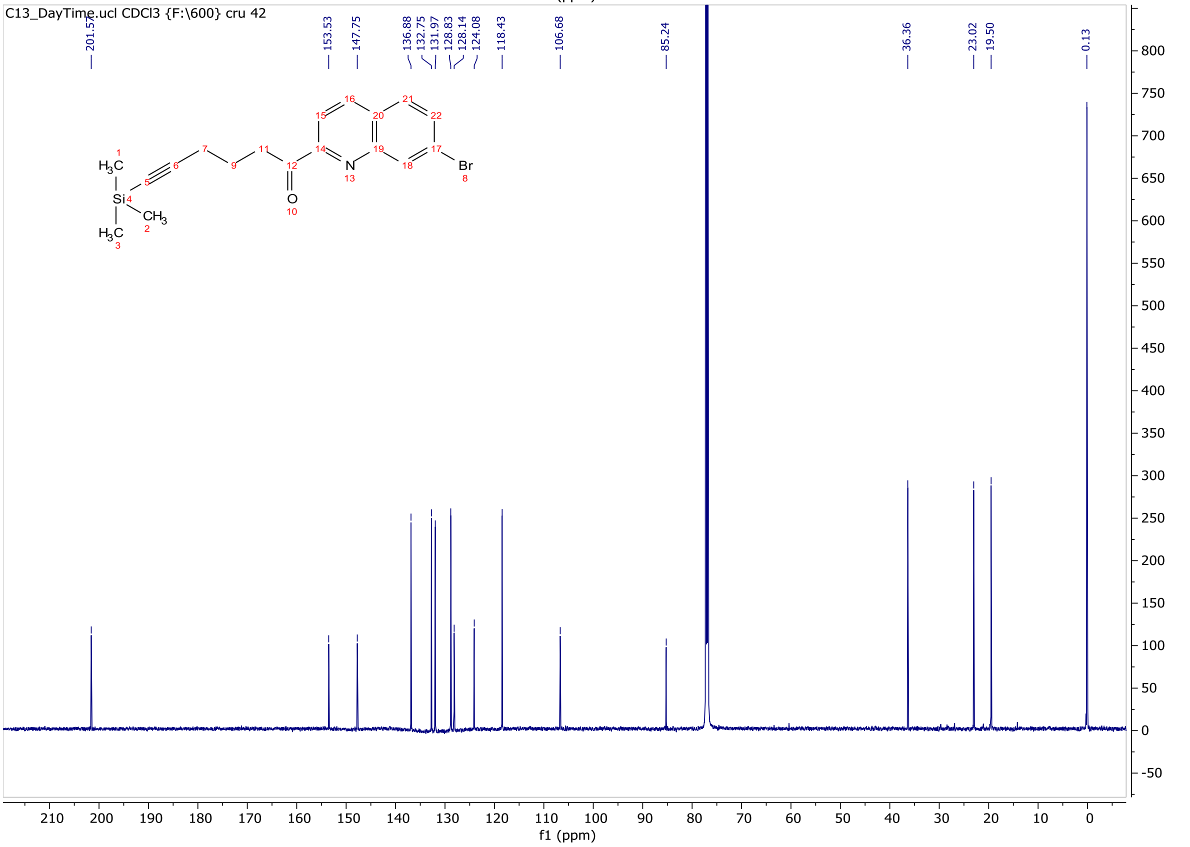
C13_DayTime.ucl CDCl3 {F:\600} cru 42
201.57
153.53
147.75
136.88
132.75
131.97
128.83
128.14
124.08
118.43
106.68
85.24
36.36
23.02
19.50
0.13
Br
O
N
Si
f1 (ppm)

(R)-1-(7-bromoquinolin-2-yl)ethan-1-ol (2a)

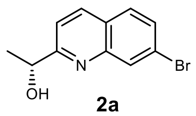

2a

dichloro(*p*-cymene)ruthenium(II) dimer (7.0 mg, 11.0 µmol, 0.6 mol-%) and (1R, 2R)-(-)-N-p-tosyl-1,2-diphenylethylenediamine (8.0 mg, 22.0 µmol, 1.1 mol-%) were added to degassed H_2O (22 mL). The yellow solution was heated to 70°C for 1.5 h under N_2. After cooling to 25°C, sodium formate (765 mg, 11.3 mmol, 5.63 eq) was added in one portion followed by the substrate ketone **s6** (500 mg, 2.00 mmol, 1.00 eq) in THF (15 mL). The reaction was stirred vigorously at 40°C and changed to a dark brown colour. After 2.5 h, completion was observed by LC-MS and the solution was extracted with EtOAc (3x). The combined organic layers were washed with brine (1x), concentrated under reduced pressure, dissolved in EtOAc, and filtered over a thiol scavenger column (Isolute Si-Thiol, Biotage). The solution was concentrated under reduced pressure to yield a brown oil (589 mg). Purification by flash chromatography (silica, 10-30% EtOAc/cHex) afforded methyl quinoline alcohol **2a** (453 mg, 1.80 mmol, 90% yield) as an off-white solid.

R_f = 0.23 in 20% EtOAc/cHex.

^{1}H NMR (700 MHz, $CDCl_3$) δ 8.28 (d, *J* = 1.9 Hz 1H); 8.14 (d, *J* = 8.5 Hz, 1H); 7.70 (d, *J* = 8.6 Hz, 1H); 7.64 (dd, *J* = 8.6 Hz, 1.9 Hz, 1H); 7.39 (d, *J* = 8.5 Hz, 1H); 5.04 (q, *J* = 6.7 Hz, 1H); 4.84 (s, 1H); 1.58 (d, *J* = 6.7 Hz, 3H).

^{13}C NMR (176 MHz, $CDCl_3$) δ 164.07, 146.86, 137.03, 131.07, 130.03, 128.80, 126.01, 124.03, 118.39, 68.84, 24.04.

HRMS (ESI+): calcd. for $[C_{11}H_{10}BrNO+H]^+$ (MH^+) 252.0019; found 252.0019.

Procedure followed from literature where absolute conformation was determined by X-Ray[10].

LC trace (254 nM)

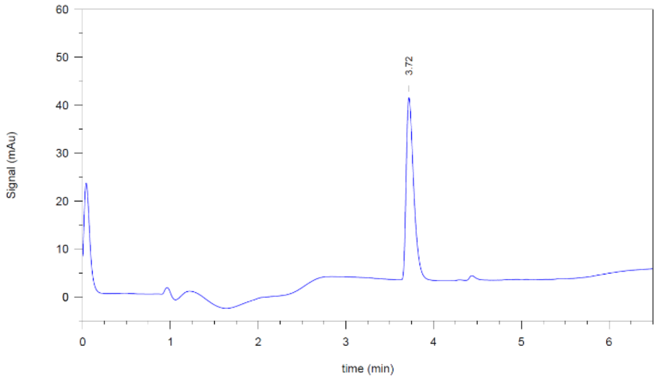

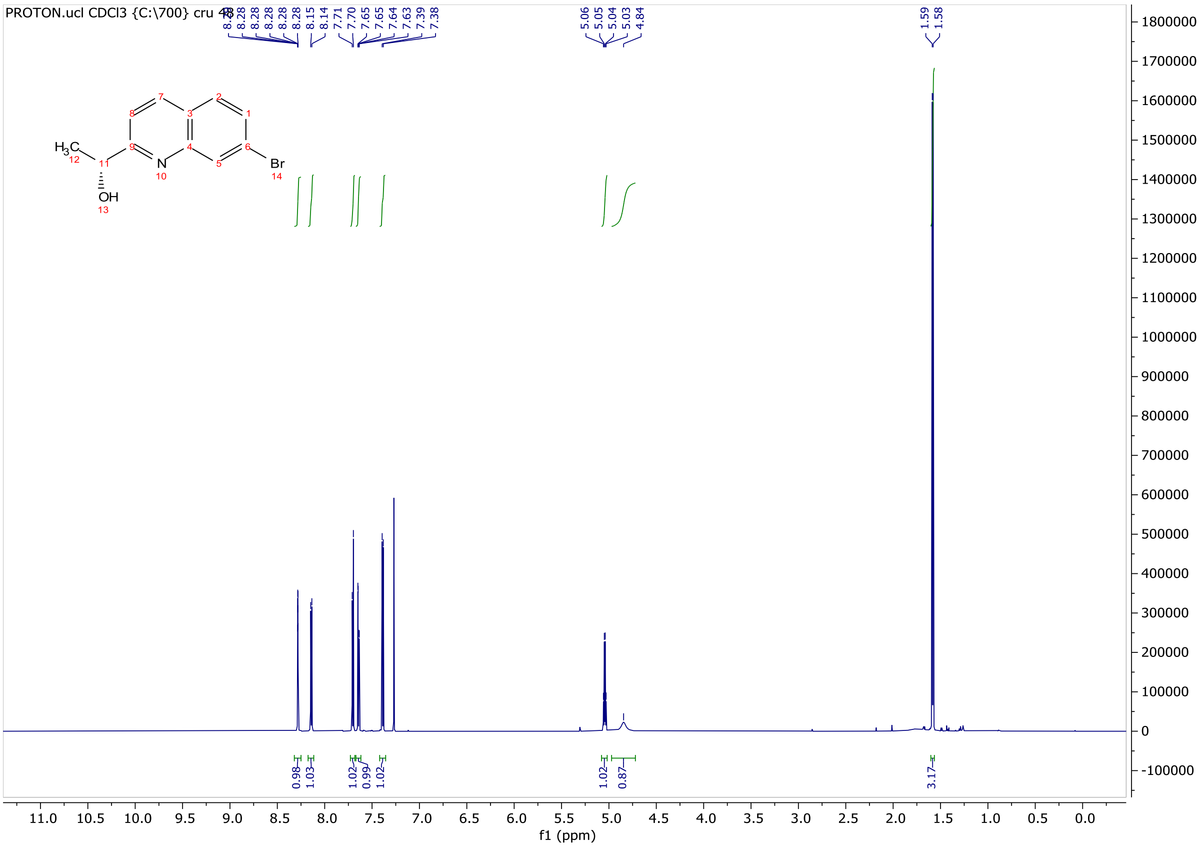
PROTON.ucl CDCl3 {C:\700} cru 48
8.28 8.28 8.28 8.28 8.28 8.28 8.15 8.14 7.71 7.70 7.65 7.65 7.64 7.63 7.39 7.38
5.06 5.05 5.04 5.03 4.84
1.59 1.58
H3C 12 11 OH 13 9 8 7 3 2 1 6 Br 14 5 4 N 10
0.98 1.03 1.02 0.99 1.02 1.02 0.87 3.17
f1 (ppm)

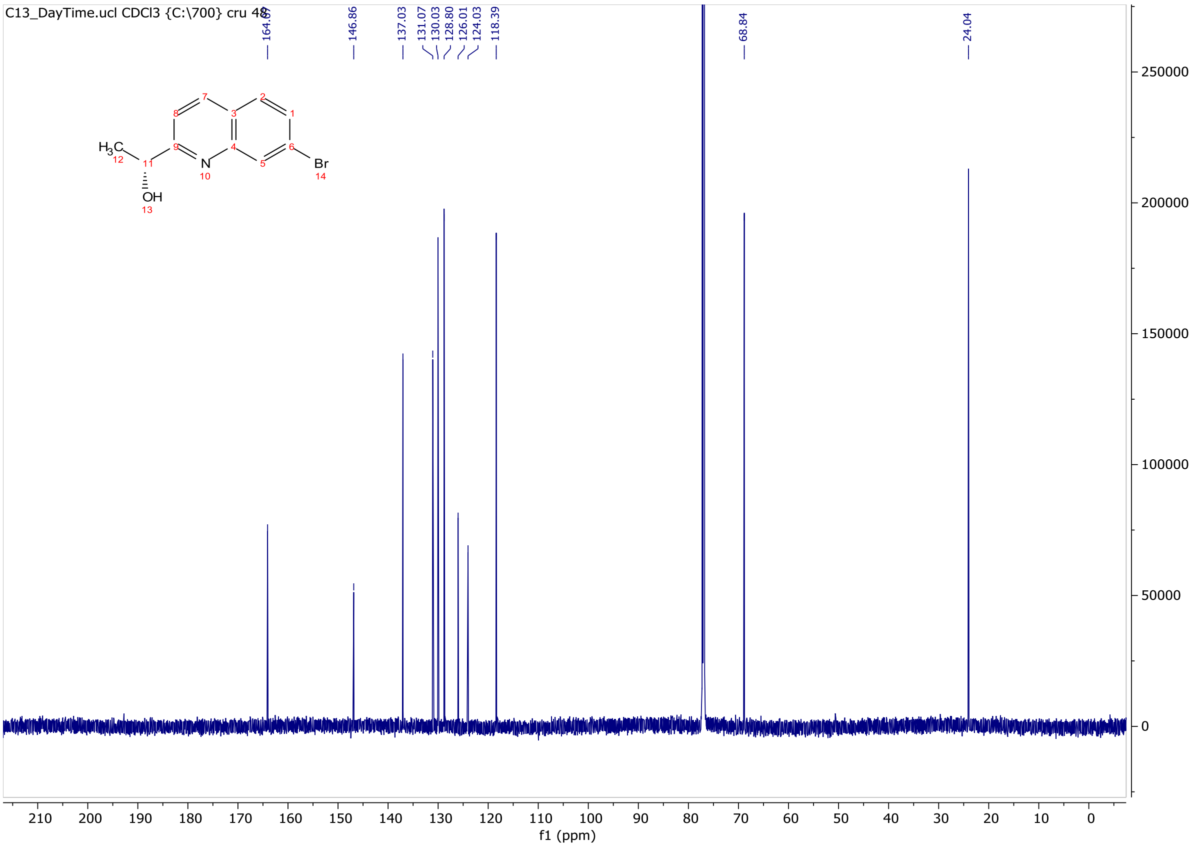
C13_DayTime.ucl CDCl3 {C:\700} cru 48
164.97 146.86 137.03 131.07 130.03 128.80 126.01 124.03 118.39 68.84 24.04
H3C 12 11 OH 13 9 8 7 3 2 1 6 Br 14 5 4 N 10
f1 (ppm)

(R)-1-(7-bromoquinolin-2-yl)-6-(trimethylsilyl)hex-5-yn-1-ol (2b)

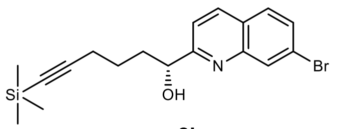

2b

dichloro(*p*-cymene) ruthenium (II) dimer (3.4 mg, 6.0 µmol, 0.5 mol-%) and (1*R*,2*R*)-(-)-*N*-*p*-tosyl-1,2-diphenylethylenediamine (4.2 mg, 10.0 µmol, 0.1 mol-%) were added to degassed H_2O (8 mL). The yellow solution was heated at 70°C for 1.5 h under N_2. After cooling to 25°C, sodium formate (398 mg, 5.85 mmol, 5.01 eq) was added in one portion followed by the substrate ketone **s7** (437 mg, 1.17 mmol, 1.00 eq) in THF (5 mL). The light orange reaction was stirred vigorously at 40°C for 3.5h, after which completion was observed by LC-MS. the solution was extracted with EtOAc (2x) and the combined organic layers were dried over $MgSO_4$, concentrated under reduced pressure, dissolved in EtOAc, and filtered over a thiol scavenger column equilibriated with EtOAc (Isolute Si-Thiol, biotage). Concentration under reduced pressure afforded a yellow oil (512 mg) which was purified by flash chromatography (silica, 0-10% EtOAc/cHex) to yield alkyne quinoline alcohol **2b** (379 mg, 1.01 mmol, 86% yield) as a transparent oil.

R_f = 0.25 in 20% EtOAc/cHex.

^{1}H NMR (600 MHz, $CDCl_3$) δ 8.28 (d, *J* = 1.9 Hz, 1H), 8.14 (d, *J* = 8.4 Hz, 1H), 7.71 (d, *J* = 8.6 Hz, 1H), 7.64 (dd, *J* = 8.6, 1.8 Hz, 1H), 7.38 (d, *J* = 8.5 Hz, 1H), 4.95 (d, *J* = 7.4 Hz, 1H), 4.84 (s, 1H), 2.30 (m, 2H), 2.14 – 2.05 (m, 1H), 1.85 – 1.71 (m, 2H), 1.68 – 1.57 (m, 1H), 0.15 (d, *J* = 0.9 Hz, 9H).

^{13}C NMR (151 MHz, $CDCl_3$) δ 162.92, 146.95, 136.83, 131.15, 130.00, 128.82, 126.03, 123.94, 118.60, 107.00, 84.87, 71.96, 36.90, 23.95, 19.64, 0.15.

HRMS (ESI+): calcd. for $[C_{18}H_{22}BrNOSi+H]^+$ (MH^+) 376.0727; found 376.0726.

*Note: NMR studies on the final molecule (CG167) are in accordance with stereocenter configuration. Er of this step could not be determined but was later deduced as >90:10 based on diastereoisomeric resolution (compound **s11**).*

LC trace (254 nM)

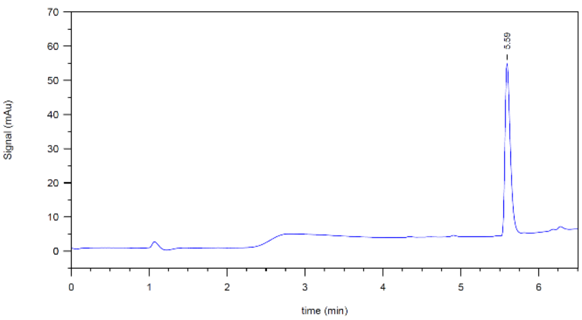

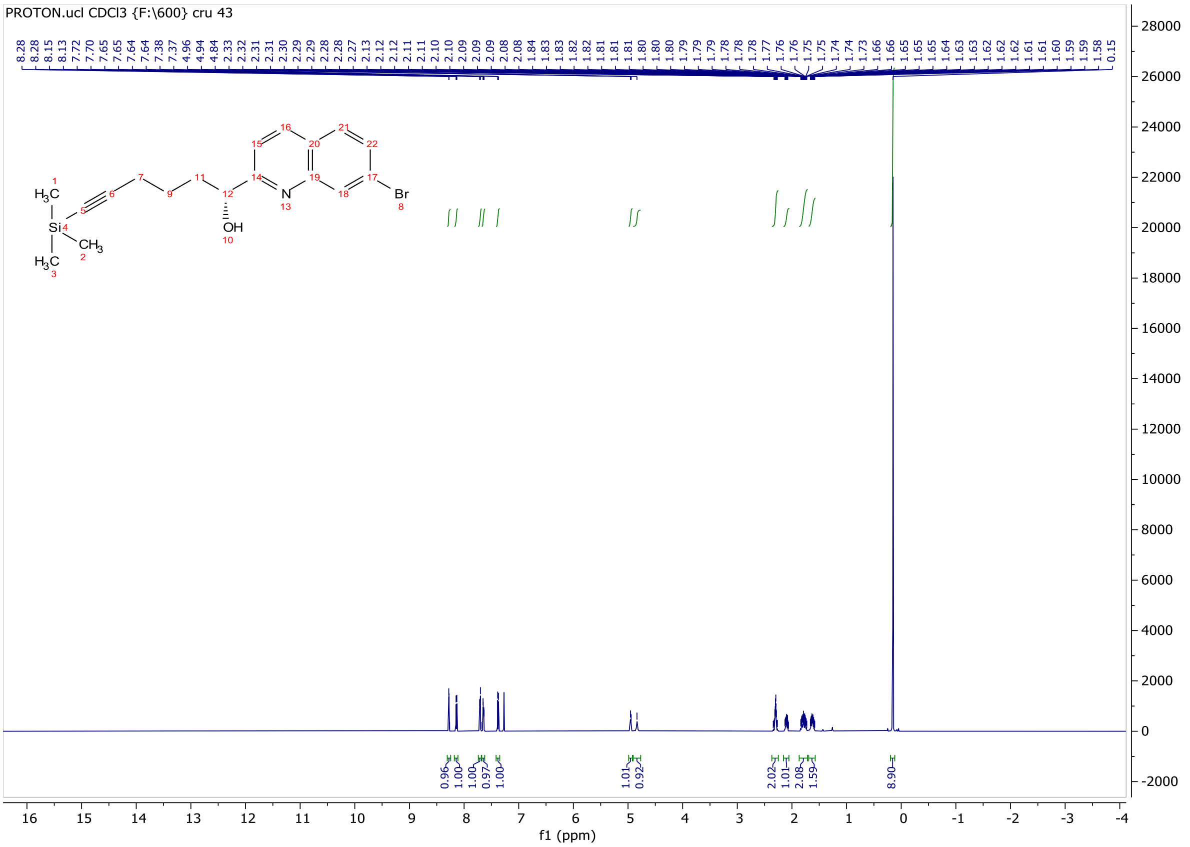
PROTON.ucl CDCl3 {F:\600} cru 43
8.28 8.28 8.15 8.13 7.72 7.70 7.65 7.65 7.64 7.64 7.38 7.37 4.96 4.94 4.84 2.33 2.32 2.31 2.31 2.30 2.29 2.29 2.28 2.28 2.27 2.13 2.12 2.12 2.11 2.11 2.10 2.10 2.09 2.09 2.09 2.08 1.84 1.83 1.83 1.82 1.82 1.81 1.81 1.80 1.80 1.80 1.79 1.79 1.78 1.78 1.78 1.77 1.76 1.76 1.75 1.75 1.74 1.74 1.73 1.66 1.66 1.65 1.65 1.65 1.64 1.63 1.63 1.62 1.62 1.61 1.61 1.60 1.59 1.59 1.58 0.15
0.96 1.00 1.00 0.97 1.00 1.01 0.92 2.02 1.01 2.08 1.59 8.90
f1 (ppm)
Br
OH
N
Si
H_3C
CH_3
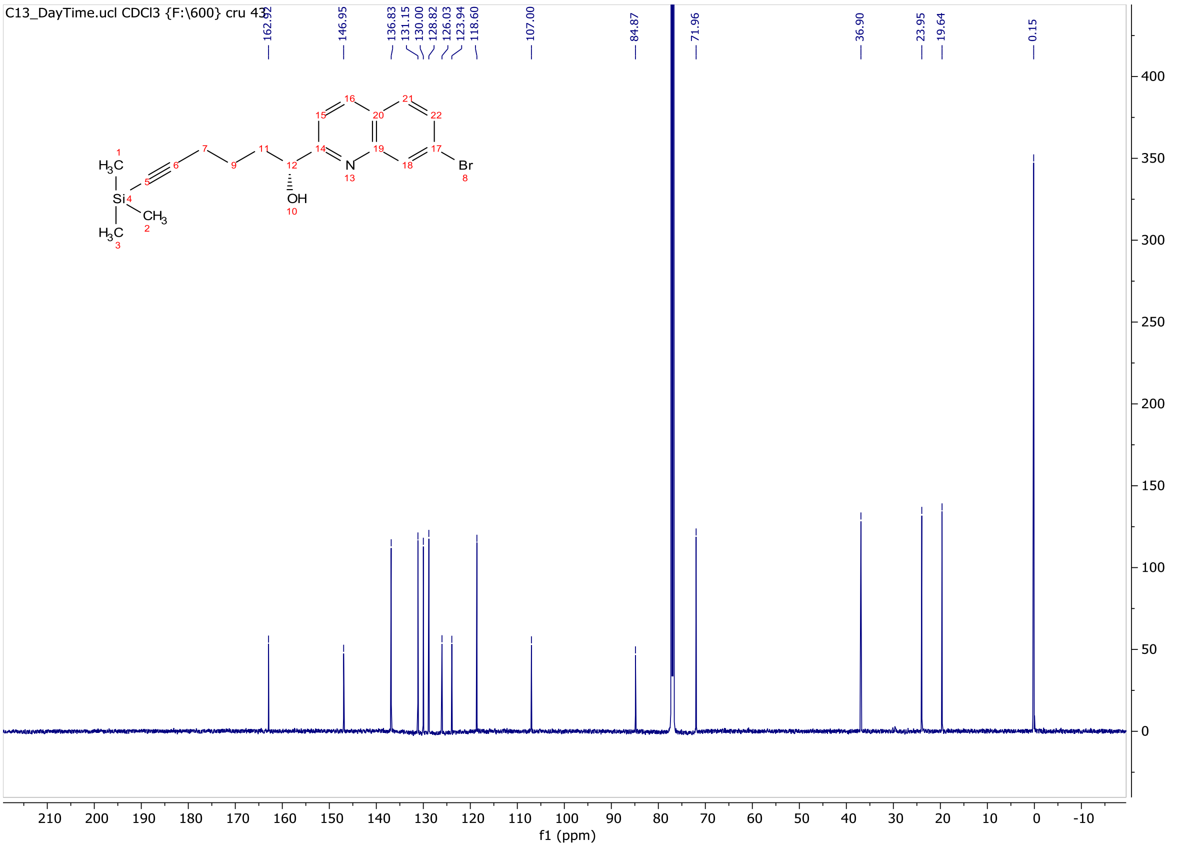
C13_DayTime.ucl CDCl3 {F:\600} cru 43
162.92 146.95 136.83 131.15 130.00 128.82 126.03 123.94 118.60 107.00 84.87 71.96 36.90 23.95 19.64 0.15
f1 (ppm)
Br
OH
N
Si
H_3C
CH_3

(S)-4-benzyl-3-(4-bromobutanoyl)oxazolidin-2-one (s8)

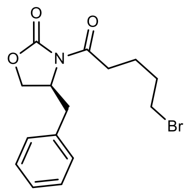

s8

(S)-4-benzyloxazolidin-2-one (2.00 g, 11.3 mmol, 1.00 eq) was dissolved in dry THF (35 mL) and cooled down to -78°C under N_2. A solution of *n*-BuLi in hexane (2.5 M, 5.0 mL, 1.06 eq) was added dropwise over 15 min to the clear solution and the reaction was stirred for further 15 min. Bromovaleroyl chloride (2.48 g, 12.4 mmol, 1.10 eq) in dry THF (35 mL) was added dropwise to the clear solution over 15 min and the reaction was stirred for further 15 min at -78°C. The solution was then warmed slowly to 25°C and further stirred for 50 min. The reaction was quenched with sat. aq. NH_4Cl (5 mL), then diluted with H_2O. The aqueous layer was extracted with CH_2Cl_2 (2x) and the organic layers were washed with brine (1x) at each step. The combined organic layers were dried over $MgSO_4$ and concentrated under reduced pressure to yield a transparent yellow oil (4.40 g). Purification by flash chromatography (silica, 0-20% EtOAc/cHex) afforded compound **s8** as a transparent oil (3.46 g, 10.2 mmol, 90% yield).

R_f = 0.41 in 20% EtOAc/cHex.

^{1}H NMR (500 MHz, $CDCl_3$) δ 7.42 – 7.32 (m, 2H), 7.32 – 7.28 (m, 1H), 7.22 (d, *J* = 7.3 Hz, 2H), 4.69 (ddt, *J* = 9.7, 6.3, 3.0 Hz, 1H), 4.26 – 4.16 (m, 2H), 3.46 (t, *J* = 6.6 Hz, 2H), 3.31 (dd, *J* = 13.3, 3.4 Hz, 1H), 3.02 (dt, *J* = 17.3, 7.2 Hz, 1H), 2.95 (dt, *J* = 17.4, 7.2 Hz, 1H), 2.78 (dd, *J* = 13.4, 9.7 Hz, 1H), 1.97 (p, *J* = 6.7 Hz, 2H), 1.93 – 1.82 (m, 2H).

^{13}C NMR (126 MHz, $CDCl_3$) δ 172.63, 153.46, 135.21, 129.40, 128.98, 127.40, 66.28, 55.15, 37.95, 34.62, 33.10, 32.02, 22.81.

HRMS (ESI+): calcd. for $[C_{15}H_{18}BrNO_3+H]^+$ (MH^+) 340.0543; found 340.0543.

LC trace (250 nM)

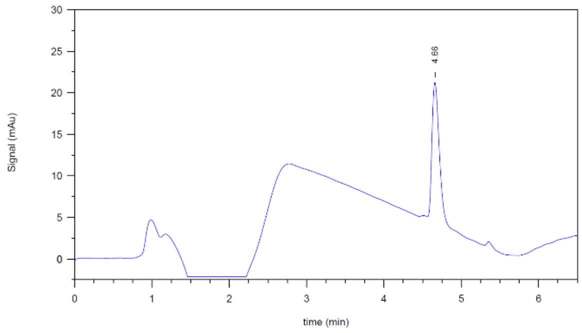

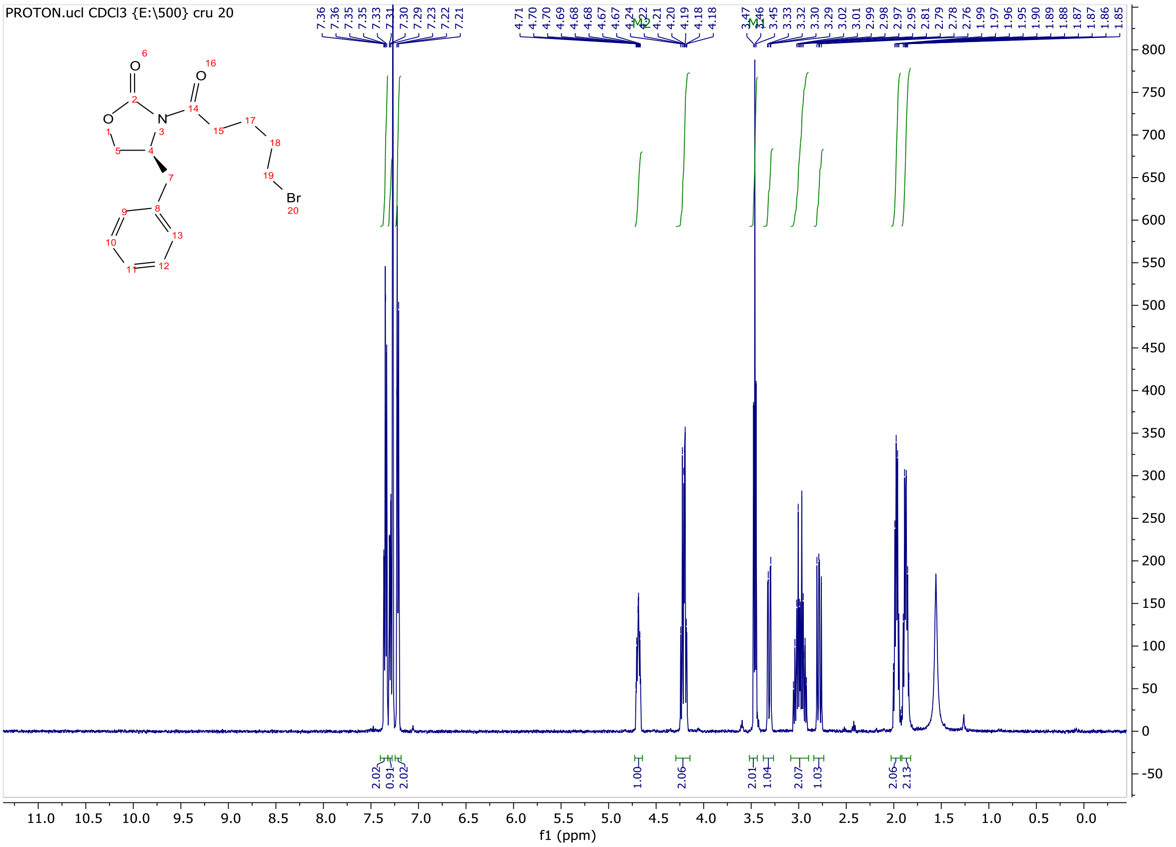
PROTON.ucl CDCl3 {E:\500} cru 20
7.36 7.36 7.35 7.35 7.33 7.31 7.30 7.29 7.23 7.22 7.21
4.71 4.70 4.69 4.68 4.68 4.67 4.67 4.24 4.22 4.21 4.20 4.19 4.18 4.18
3.47 3.46 3.45 3.33 3.32 3.30 3.29 3.02 3.01 2.99 2.98 2.97 2.95 2.81 2.79 2.78 2.76 1.99 1.97 1.96 1.95 1.90 1.89 1.88 1.87 1.86 1.85
M2
M1
2.02 0.91 2.02 1.00 2.06 2.01 1.04 2.07 1.03 2.06 2.13
Br
f1 (ppm)

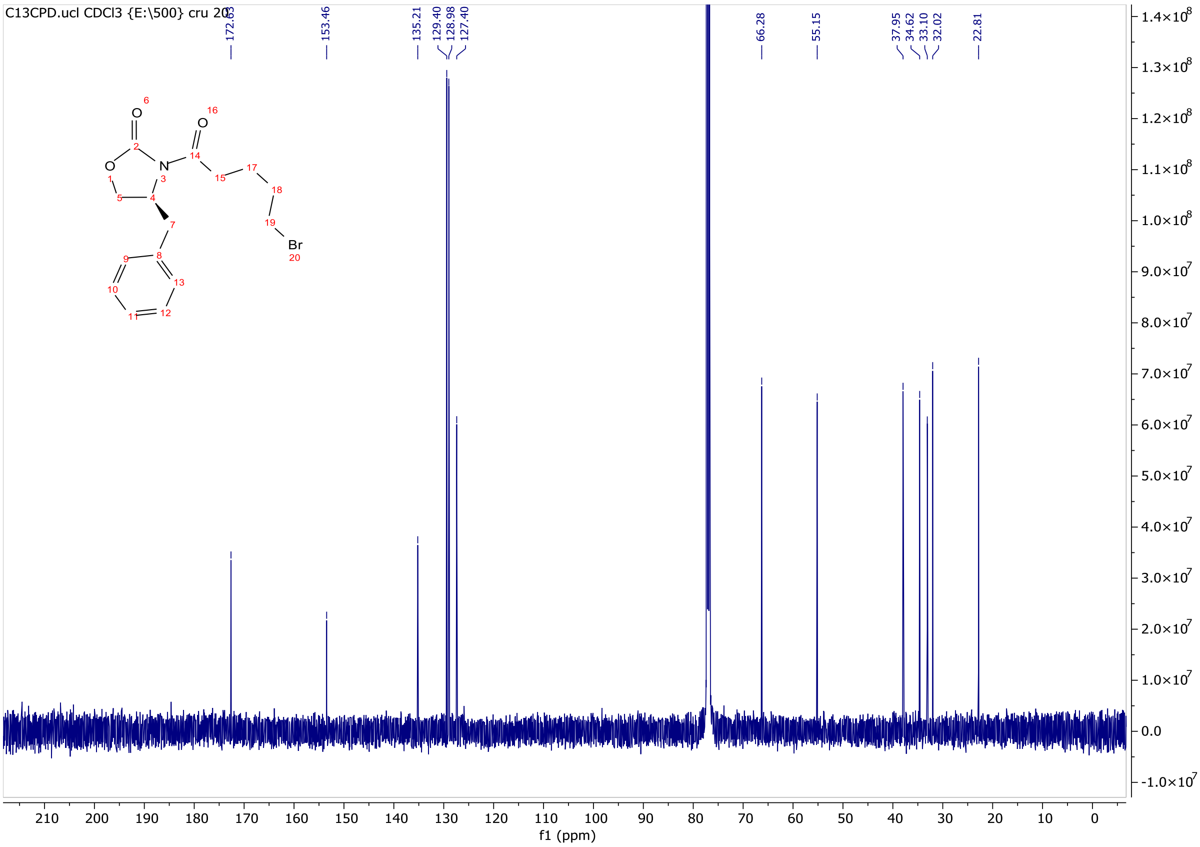
C13CPD.ucl CDCl3 {E:\500} cru 20
172.63
153.46
135.21 129.40 128.98 127.40
66.28
55.15
37.95 34.62 33.10 32.02
22.81
Br
f1 (ppm)

(S)-1,2-bis(Boc)hexahydropyridazine-3-carboxylic acid (4)
Adapted from Hale *et al*[11, 12].

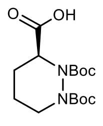

4

Oxazolidinone alkyl bromide **s8** (3.34 g, 9.82 mmol, 1.00 eq) was dissolved in dry THF (20 mL) and cooled down to -78°C under N_2. A solution of LDA (7.2 mL, 1.35 M in Heptane/THF/Ethylbenzene, 1.10 eq) was added dropwise over 15 min. The yellow solution was stirred at -78°C for 1 h, after which di-*tert*-butyl azodicarboxylate (2.59 g, 11.1 mmol, 1.15 eq) in dry CH_2Cl_2 (10 mL) was added dropwise for 15 min. After further 1 h stirring, DMPU (20 mL, 165 mmol, 16.8 eq) was added dropwise for 30 min. The reaction was then stirred for 25 min, slowly warmed up to 25°C and stirred for 5 min. The reaction mixture was poured onto Et_2O layered over sat. aq. K_2HPO_4 and briefly shaken. The layers were separated, and the organic layer was washed with H_2O (2x). The combined aqueous layers (pH = 7) were acidified to pH = 2 with a few drops conc. HCl and then solid $NaHSO_4$ and extracted with Et_2O (2x). The combined organic layers were washed with H_2O (1x). The organic layer was dried over $MgSO_4$ and concentrated under reduced pressured to yield an orange oil (7.01 g).

The crude oil was dissolved in THF (40 mL) at 0°C and a solution of $LiOH{\cdot}H_2O$ (900 mg, 21.4 mmol, 2.19 eq) in H_2O (20 mL) was added dropwise. The resulting mixture was stirred for 2.5 h at 0°C, after which completion of hydrolysis was observed by LC-MS. The solution was diluted with EtOAc and washed with sat. aq. $NaHCO_3$ (3x). The aqueous layers were then combined and acidified to pH 4 with 6 M aq. HCl and extracted with EtOAc (3x). The combined EtOAc extracts were dried over $MgSO_4$ and concentrated under reduced pressure to yield a yellow oil (3.46 g). Purification by flash chromatography (C18, 20-100% MeCN/H_2O 0.1% FA) afforded Boc-protected (3S)-piperazic acid **4** as an off-white solid (2.04 g, 6.28 mmol, 63% yield).

^{1}H NMR (600 MHz, CD_3OD) δ 4.79 (br s, partially hidden by H_2O peak), 4.11 – 3.97 (br m, 1H), 3.10 – 2.80 (br m, 1H), 2.12 – 2.00 (br m, 1H), 1.95 – 1.70 (br m, 2H), 1.64 – 1.39 (br m, 19H) (rotamers present).

^{13}C NMR (151 MHz, CD_3OD) δ 173.06, 172.88, 155.96, 155.87, 155.15, 155.00, 83.50, 83.25, 82.88, 82.57, 81.90, 58.39, 57.24, 56.38, 55.52, 45.99, 45.70, 43.98, 28.56, 28.49, 28.45, 28.39, 25.42, 21.35, 21.02, 20.91. (rotamers present multiplying signals, in accordance with literature[11, 12])

HRMS (ESI+): calcd. for $[C_{15}H_{26}N_2O_6+Na]^+$ (MNa^+) 353.1683; found 353.1679.

LC trace (TIC+)

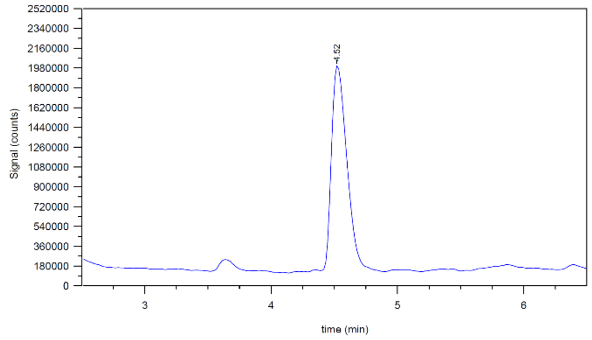

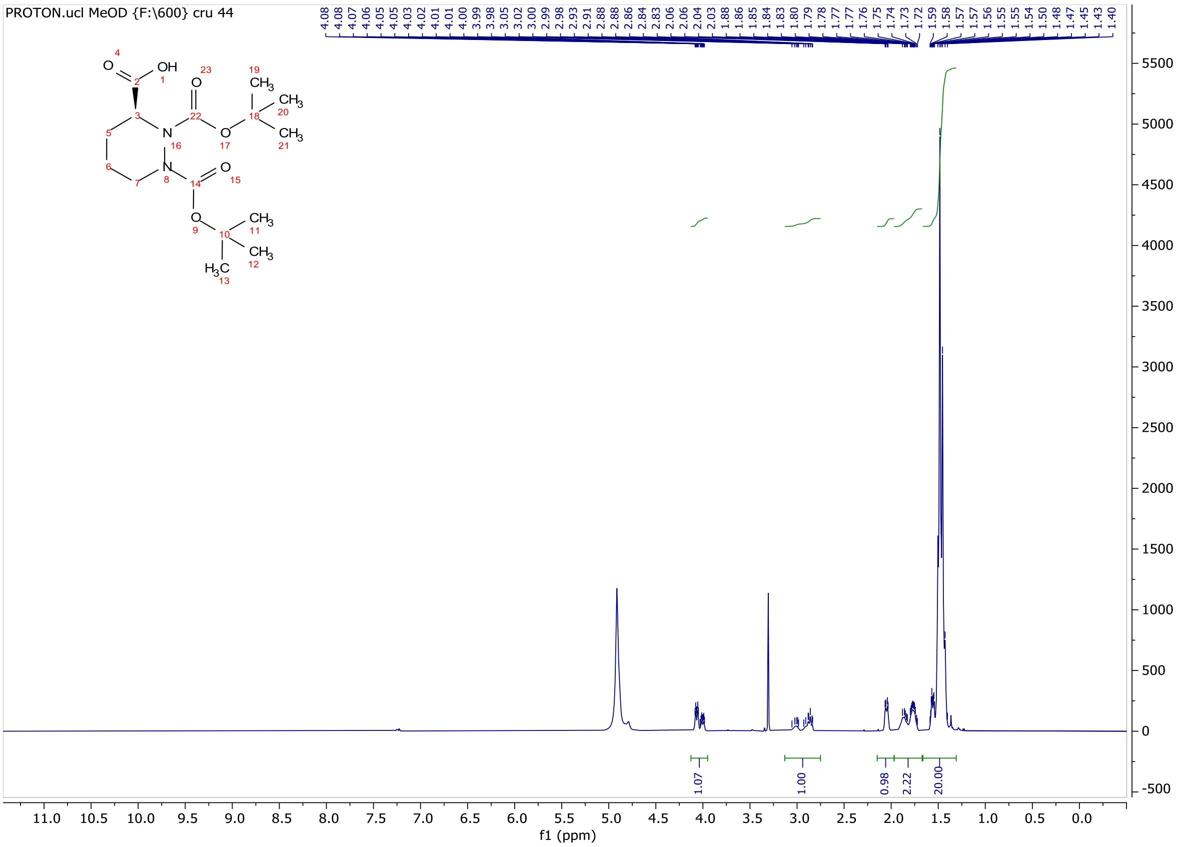
PROTON.ucl MeOD {F:\600} cru 44
f1 (ppm)

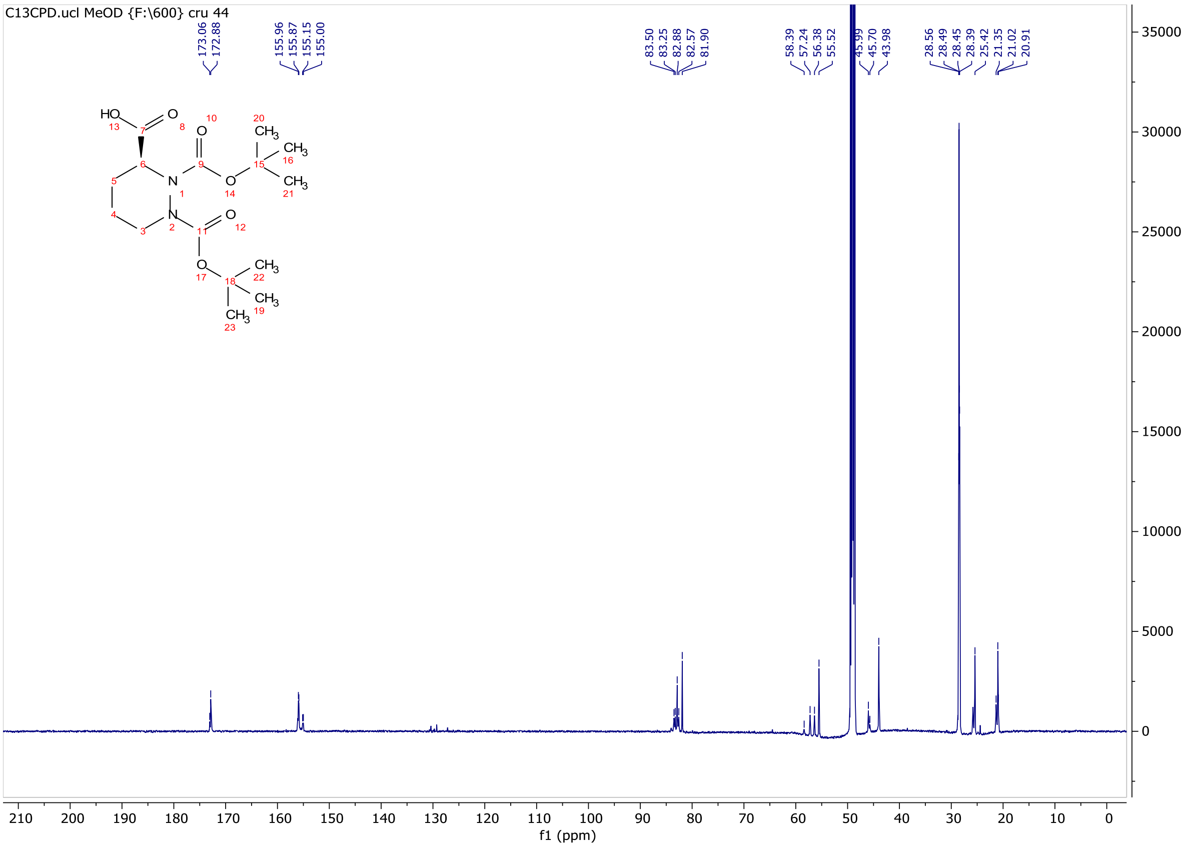
C13CPD.ucl MeOD {F:\600} cru 44
f1 (ppm)

3-((R)-1-(7-bromoquinolin-2-yl)ethyl) 1,2-di-tert-butyl (S)-tetrahydropyridazine-1,2,3-tricarboxylate (s9)

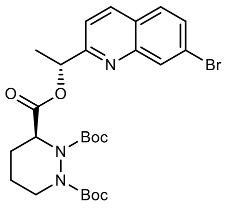

s9

Quinoline alcohol **2a** (390 mg, 1.55 mmol, 1.00 eq), Boc-protected (3S)-piperazic acid **4** (590 mg, 1.79 mmol, 1.18 eq), DMAP (20 mg, 0.164 mmol, 11 mol-%), EDC (591 mg, 3.08 mmol, 1.99 eq) and *i*Pr_2NEt (540 µL, 3.10 mmol, 2.00 eq) were combined in dry CH_2Cl_2 (25 mL). The clear reaction was stirred at 25°C for 15 h after which completion was observed by LC-MS. The solution was diluted with CH_2Cl_2 and sat. aq. NH_4Cl. The layers were separated, and the aqueous layer was extracted with CH_2Cl_2 (1x). The organic layer was dried over $MgSO_4$ and concentrated under reduced pressure to yield a brown oil (1.68 g). The oil was purified by flash chromatography (silica, 10-20 % EtOAc/cHex) to afford ester **s9** as a white solid (685 mg, 1.21 mmol, 78% yield).

R_f = 0.22 in 20 % EtOAc/cHex.

^{1}H NMR (400 MHz, DMSO-d_6, 60°C) δ 8.40 (d, *J* = 8.7 Hz, 1H), 8.20 (d, *J* = 2.0 Hz, 1H), 7.95 (d, *J* = 8.7 Hz, 1H), 7.73 (dd, *J* = 8.7, 2.0 Hz, 1H), 7.62 (br d, *J* = 8.5 Hz, 1H), 5.95 (br s, 1H), 4.95 (br s, 1H), 3.96 (s, 1H), 2.85 (s, 1H), 1.99 (s, 1H), 1.74 (s, 2H), 1.62 (d, *J* = 6.7 Hz, 3H), 1.54 (d, *J* = 5.1 Hz, 1H), 1.43 (s, 9H), 1.23 (very br s, 9H).

*Note: High temperature ^{1}H NMR cleared up spectrum, ^{13}C NMR not analysable due to rotamers. See compound **7a** and **s14** for full characterisation of next step).*

HRMS (ESI+): calcd. for $[C_{26}H_{34}BrN_3O_6+H]^+$ (MH^+) 564.1703; found 564.1704.

LC trace (254 nM)`

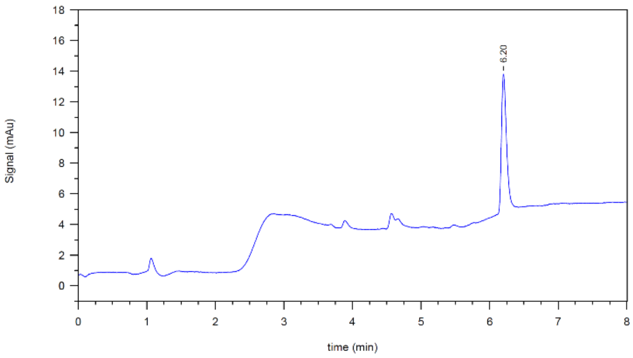

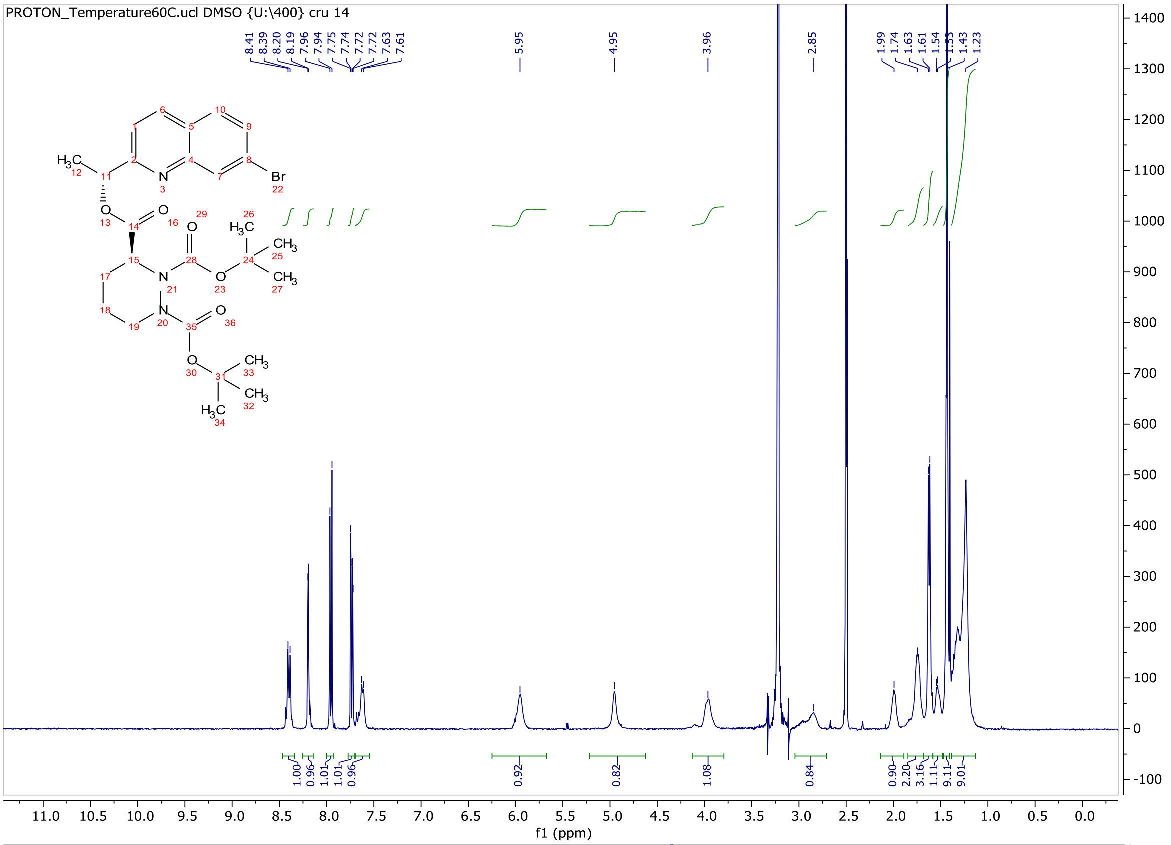
PROTON_Temperature60C.ucl DMSO {U:\400} cru 14
8.41
8.39
8.20
8.19
7.96
7.94
7.75
7.74
7.72
7.72
7.63
7.61
5.95
4.95
3.96
2.85
1.99
1.74
1.63
1.61
1.54
1.53
1.43
1.23
1.00
0.96
1.01
1.01
0.96
0.92
0.82
1.08
0.84
0.90
2.20
3.16
1.11
9.11
9.01
f1 (ppm)

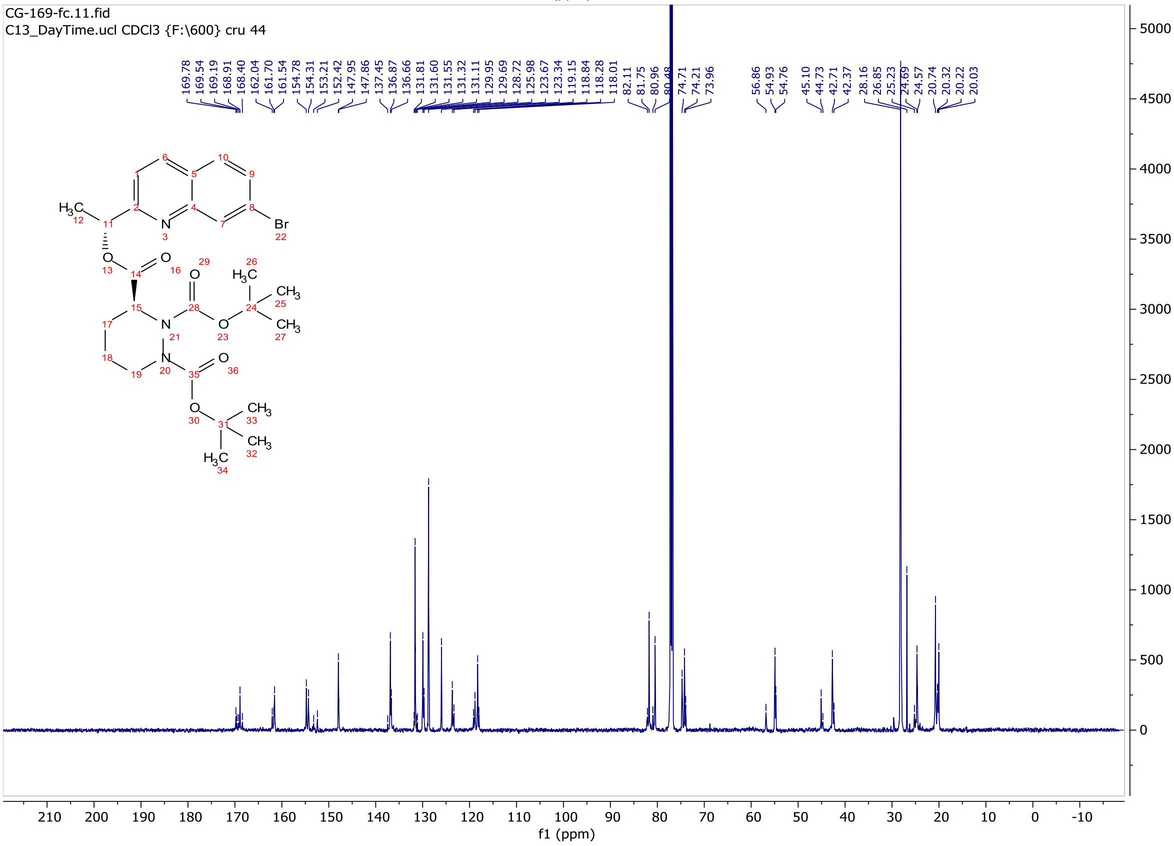
CG-169-fc.11.fid
C13_DayTime.ucl CDCl3 {F:\600} cru 44
169.78
169.54
169.19
168.91
168.40
162.04
161.70
161.54
154.78
154.31
153.21
152.42
147.95
147.86
137.45
136.87
136.66
131.81
131.60
131.55
131.32
131.11
129.95
129.69
128.72
125.98
123.67
123.34
119.15
118.84
118.28
118.01
82.11
81.75
80.96
80.48
74.71
74.21
73.96
56.86
54.93
54.76
45.10
44.73
42.71
42.37
28.16
26.85
25.23
24.69
24.57
20.74
20.32
20.22
20.03
f1 (ppm)

3-((R)-1-(7-bromonaphthalen-2-yl)-6-(trimethylsilyl)hex-5-yn-1-yl) 1,2-di-tert-butyl (S)-tetrahydropyridazine-1,2,3-tricarboxylate (s10)

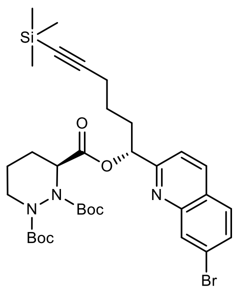

s10

Quinoline alcohol **2b** (350 mg, 0.93 mmol, 1.00 eq), Boc-protected (3S)-piperazic acid **4** (396 mg, 1.20 mmol, 1.29 eq), DMAP (15.0 mg, 0.12 mmol, 13 mol-%) and *i*Pr_2NEt (405 µL, 2.33 mmol, 2.50 eq) were combined in dry CH_2Cl_2 (17 mL). The clear yellow solution was stirred at 25°C for 24 h. The solution was diluted with EtOAc (40 mL) and sat. aq. NH_4Cl was added. The layers were separated, and the organic layer was washed with brine (1x). The aqueous layers were extracted with EtOAc (1x) at each step. The combine organic layers were dried over $MgSO_4$ and concentrated under reduced pressure to yield a yellowish oil (613 mg). Purification by flash chromatography (silica, 0-10% EtOAc/cHex) afforded ester **s10** as a transparent oil (603 mg, 0.88 mmol, 95% yield).

HRMS (ESI+): calcd. for $[C_{33}H_{46}BrN_3O_6Si+H]^+$ (MH^+) 688.2412; found 688.2411.

R_f = 0.34 in 20% EtOAc/cHex.

LC trace (214 nM)

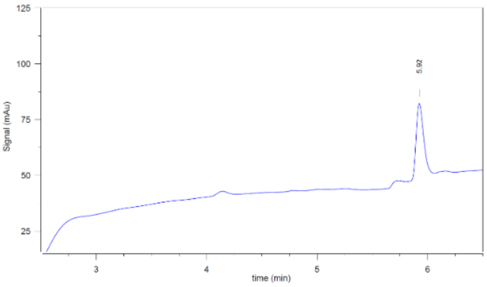

(R)-1-(7-bromoquinolin-2-yl)hex-5-yn-1-yl (S)-3,4,5,6-tetrahydropyridazine-3-carboxylate (s11)

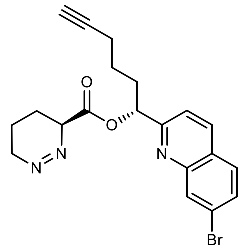

s11

Rotamers make NMR analysis impractical even at high temperature, so **s10** (20 mg, 26 µmol, 1.00 eq) was stirred at 25°C in 1:1 CH_2Cl_2:TFA (0.4 mL) for 2h to remove the Boc groups. The deprotected ring is not stable and oxidises readily. The reaction was therefore concentrated, and the residue let to stand to air for several days, to afford the TH-pyridazine compound **s11** (14 mg, 26 µmol, quant.).

NMR indicates the presence of ≤5% diastereoisomers, mainly visible at shifts of the TH-pyridazine ester ring. The isomer at the C-O bond was undetectable by 1D NMR but could be detected in 2D NMR cross peaks. This implies that enantiomeric ratios were >90:10 in the starting materials (95% yield * 95% purity = 90%). Absolute configuration was studied by NMR on the final molecule (CG167) as NOEs were not useful here.

^1H NMR (500 MHz, $CDCl_3$) δ 8.31 (s, 1H), 8.13 (d, *J* = 8.5 Hz, 1H), 7.67 (d, *J* = 8.7 Hz, 1H), 7.61 (dd, *J* = 8.7, 1.9 Hz, 1H), 7.54 (d, *J* = 8.6 Hz, 1H), 6.52 (br s, 1H), 6.09 (t, *J* = 6.8 Hz, 1H), 3.28 (t, *J* = 5.5 Hz, 2H), 2.49 (td, *J* = 6.7, 3.2 Hz, 2H), 2.26 (td, *J* = 7.2, 2.7 Hz, 2H), 2.23 (dt, *J* = 7.5, 7.0 Hz, 2H), 1.94 (t, *J* = 2.7 Hz, 1H), 1.98 – 1.89 (m, 2H), 1.76 (dt, *J* = 15.1, 7.0 Hz, 1H), 1.69 (dt, *J* = 15.0, 7.0 Hz, 1H).

^{13}C NMR (126 MHz, $CDCl_3$) δ 164.31, 161.29, 147.99, 137.01, 131.54, 130.04, 128.72, 126.07, 123.84, 118.77, 83.89, 76.55, 68.71, 41.87, 34.06, 24.57, 21.15, 18.22, 17.36. (*α*-C to C=O not visible*).*

HRMS (ESI+): calcd. for $[C_{20}H_{20}BrN_3O_2H]^+$ (MH^+) 414.0812; found 414.0816.

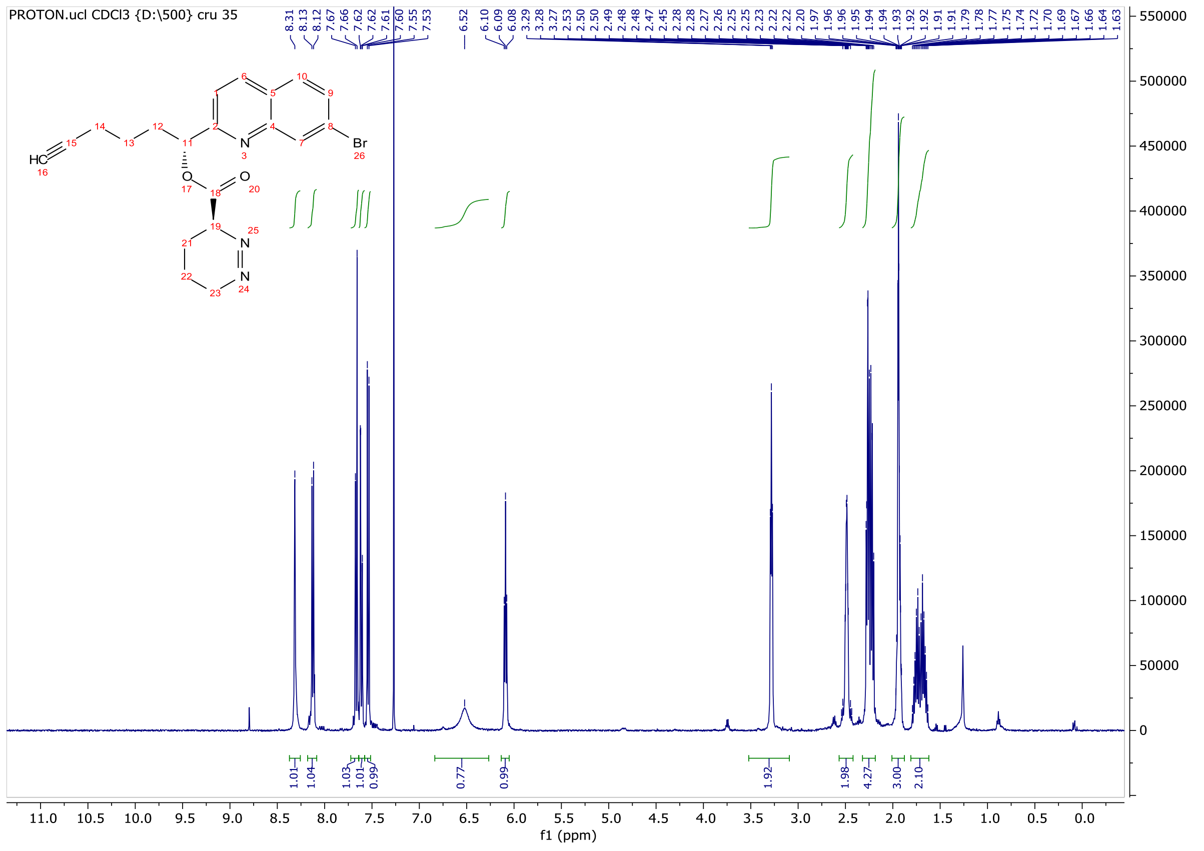

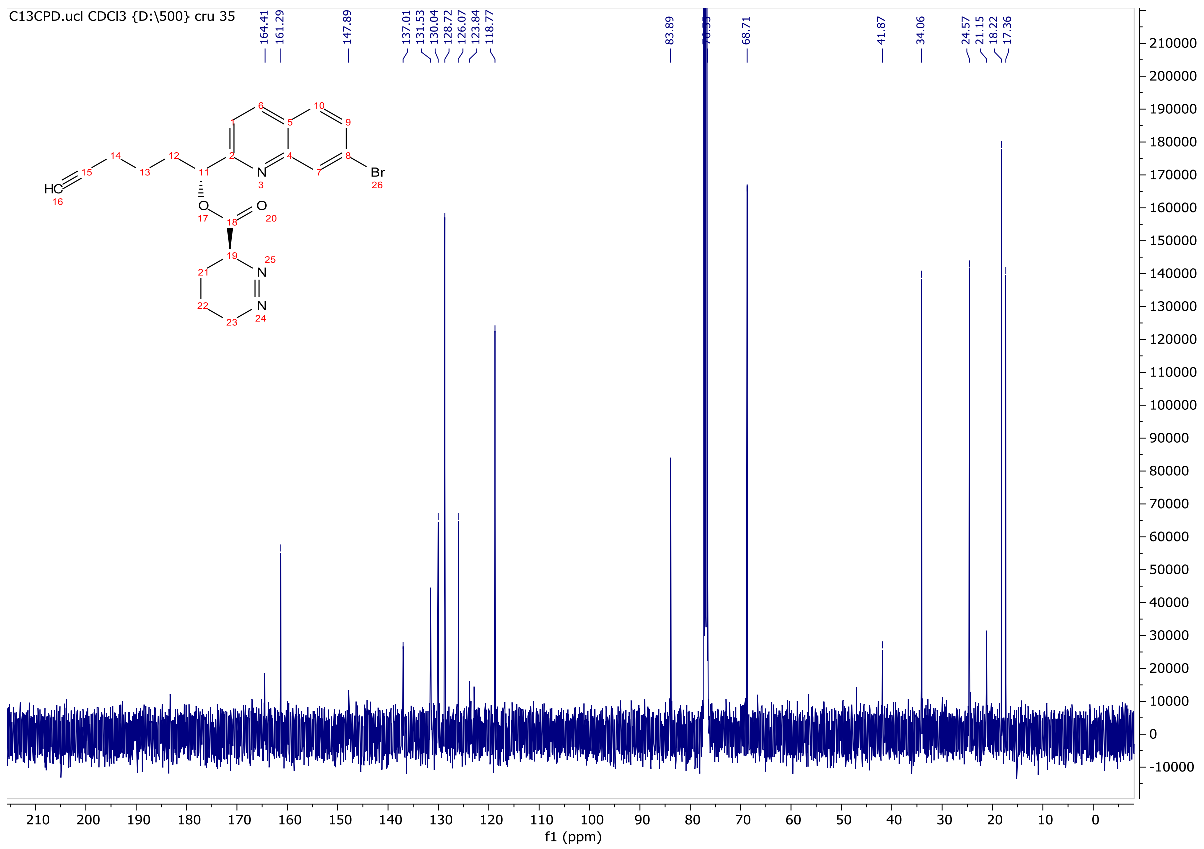

Note: broad signal is proton α to carbonyl of piperazic ester, which undergoes exchange. Carbon shift absent as confirmed by HSQC.

methyl ((S)-2-((tert-butoxycarbonyl)amino)-3-(4-nitrophenyl)propanoyl)-L-alaninate (s12)

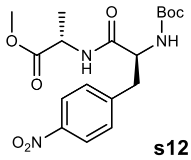

s12

To a solution of N-Boc-(4-nitro)phenylalanine (225 mg, 0.72 mmol, 1.00 eq) and alanine methyl ester hydrochloride (102 mg, 0.72 mmol, 1.00 eq) in dry MeCN (4 mL) was added HATU (286 mg, 0.752 mmol), OxymaPure (102 mg, 0.72 mmol, 1.00 eq) and *i*Pr_2NEt (374 µL, 2.15 mmol, 3.00 eq). The reaction was stirred for 15h, after which completion was observed by LC-MS. The solution was diluted with EtOAc, then washed with H_2O (1x) and brine (1x). The organic layer was dried over $MgSO_4$ and concentrated under reduced pressure. Purification by flash chromatography (C18, 10-100% MeCN/H_2O, 0.1% FA) afforded peptide **s12** as a light-brown solid (147 mg, 0.37 mmol, 52 % yield).

^{1}H NMR (600 MHz, Acetone-d_6) δ 8.16 (d, *J* = 8.5 Hz, 2H), 7.69 (br s, 1H), 7.57 (d, *J* = 8.4 Hz, 2H), 6.15 (d, *J* = 8.9 Hz, 1H), 4.52 – 4.42 (m, 2H), 3.69 (s, 3H), 3.34 (dd, *J* = 13.9, 5.2 Hz, 1H), 3.04 (dd, *J* = 13.9, 9.0 Hz, 1H), 1.36 (d, *J* = 7.2 Hz, 3H), 1.32 (s, 9H).

^{13}C NMR (151 MHz, Acetone-d_6) δ 173.73, 171.47, 156.16, 147.79, 147.13, 131.67, 124.04, 79.52, 55.89, 52.49, 48.88, 38.85, 28.49, 17.91.

LCMS calcd. for $[C_{18}H_{25}N_3O_7Na]^+$ (MNa^+) 418.17; found 418.2.

LC Trace (254 nM).

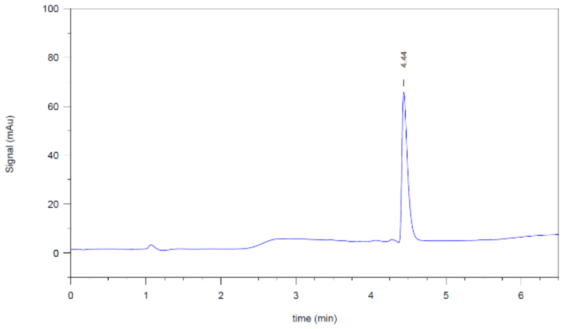

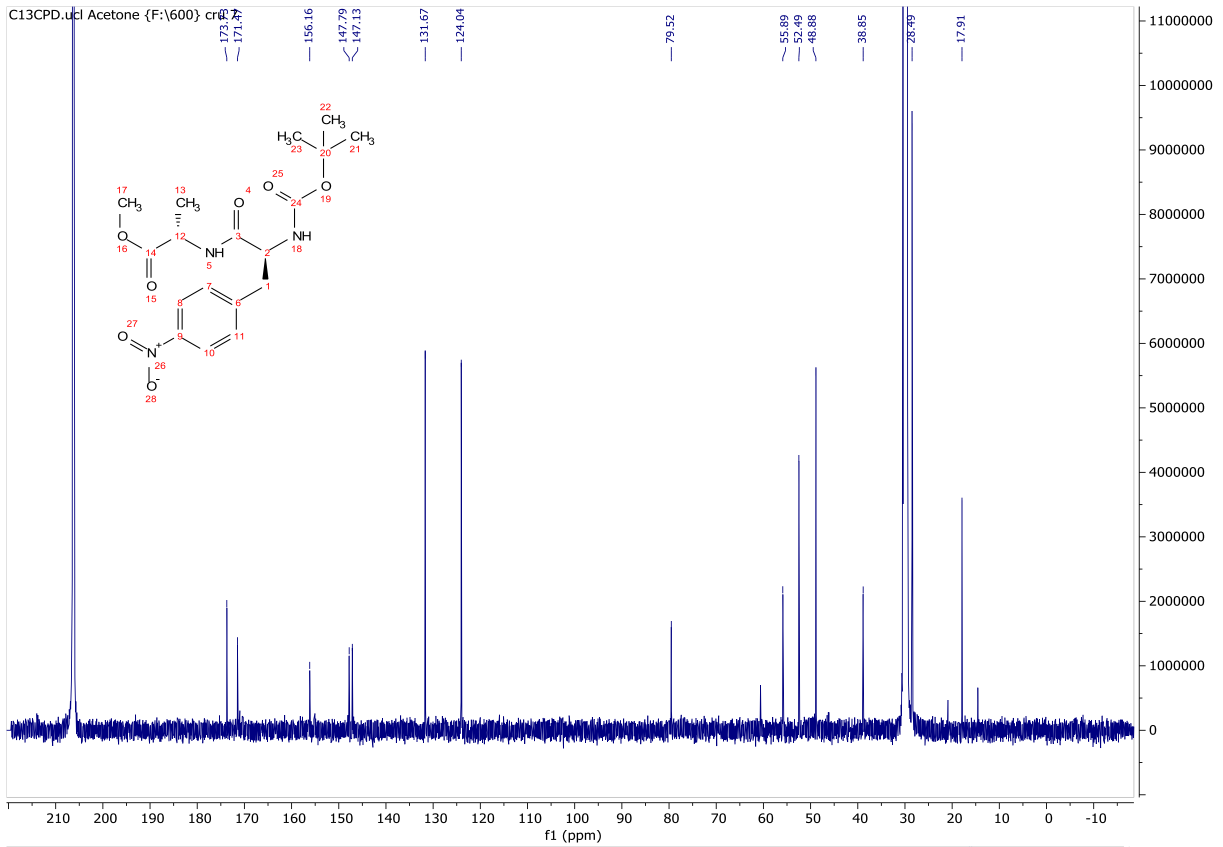
C13CPD.ucl Acetone {F:\600} cru 7
173.7
171.47
156.16
147.79
147.13
131.67
124.04
79.52
55.89
52.49
48.88
38.85
28.49
17.91
f1 (ppm)

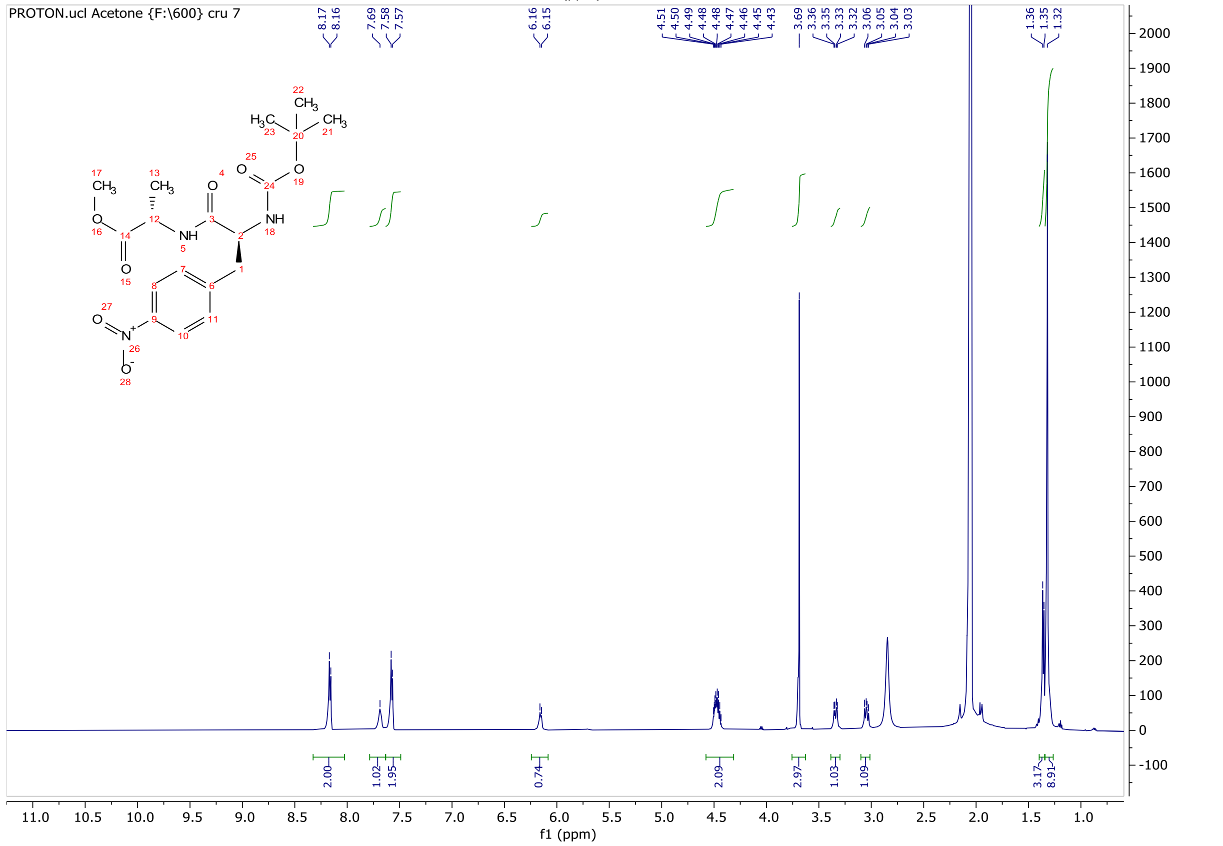
PROTON.ucl Acetone {F:\600} cru 7
8.17
8.16
7.69
7.58
7.57
6.16
6.15
4.51
4.50
4.49
4.48
4.48
4.47
4.46
4.45
4.43
3.69
3.36
3.35
3.33
3.32
3.06
3.05
3.04
3.03
1.36
1.35
1.32
2.00
1.02
1.95
0.74
2.09
2.97
1.03
1.09
3.17
8.91
f1 (ppm)

methyl ((S)-2-(but-3-enamido)-3-(4-nitrophenyl)propanoyl)-L-alaninate (s13)

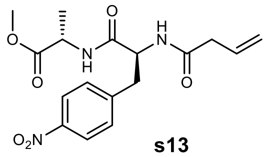

s13

Boc-protected peptide **s12** (432 mg, 1.09 mmol, 1.00 eq) was dissolved in 1:1 TFA:CH_2Cl_2 (4 mL). The reaction was stirred for 1.5 h at 25°C, after which completion was observed by LC-MS. The solution was concentrated under reduced pressure and the residue was dissolved in dry MeCN (5 mL). *i*Pr_2NEt (761 µL, 4.37 mmol, 4.00 eq) was added, followed by 3-butenoic acid (97 µL, 1.15 mmol, 1.05 eq) and PyAOP (599 mg, 1.15 mmol, 1.05 eq). The reaction was stirred at 25°C for 15 h, showing completion by LC-MS. The solution was diluted with EtOAc, then washed with H_2O (x1) and brine (x1). The organic layer was dried over $MgSO_4$ and concentrated under reduced pressure. Purification by flash chromatography (C18, 10-100% MeCN/H_2O, 0.1% FA) afforded peptide **s12** as a light-yellow powder (164 mg, 0.45 mmol, 41% yield).

^{1}H NMR (600 MHz, Acetone-d6) δ 8.13 (d, *J* = 8.7 Hz, 2H), 7.76 (d, *J* = 6.9 Hz, 1H), 7.53 (d, *J* = 8.7 Hz, 2H), 7.30 (d, *J* = 8.4 Hz, 1H), 5.84 (ddt, *J* = 17.2, 10.2, 7.0 Hz, 1H), 5.06 (dq, *J* = 17.2, 1.7 Hz, 1H), 5.02 (dq, *J* = 7.2, 1.3 Hz, 1H), 4.80 (td, *J* = 8.3, 5.4 Hz, 1H), 4.42 (p, *J* = 7.3 Hz, 1H), 3.67 (s, 3H), 3.30 (dd, *J* = 13.8, 5.4 Hz, 1H), 3.03 (dd, *J* = 13.8, 8.2 Hz, 1H), 2.94 (dd, *J* = 7.0, 1.4 Hz, 2H), 1.33 (d, *J* = 7.3 Hz, 3H).

^{13}C NMR (151 MHz, Acetone-d6) δ 173.53, 171.08, 170.61, 147.67, 146.61, 133.14, 131.59, 123.88, 117.95, 54.01, 52.35, 48.77, 41.45, 38.62, 17.65.

LCMS calcd. for $[C_{17}H_{21}N_3O_6Na]^+$ (MNa^+) 386.14; found 386.2

LC trace (254 nM)

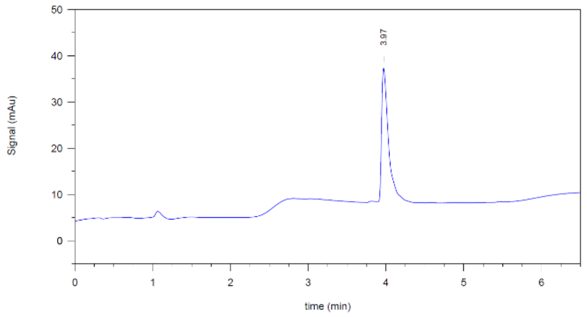

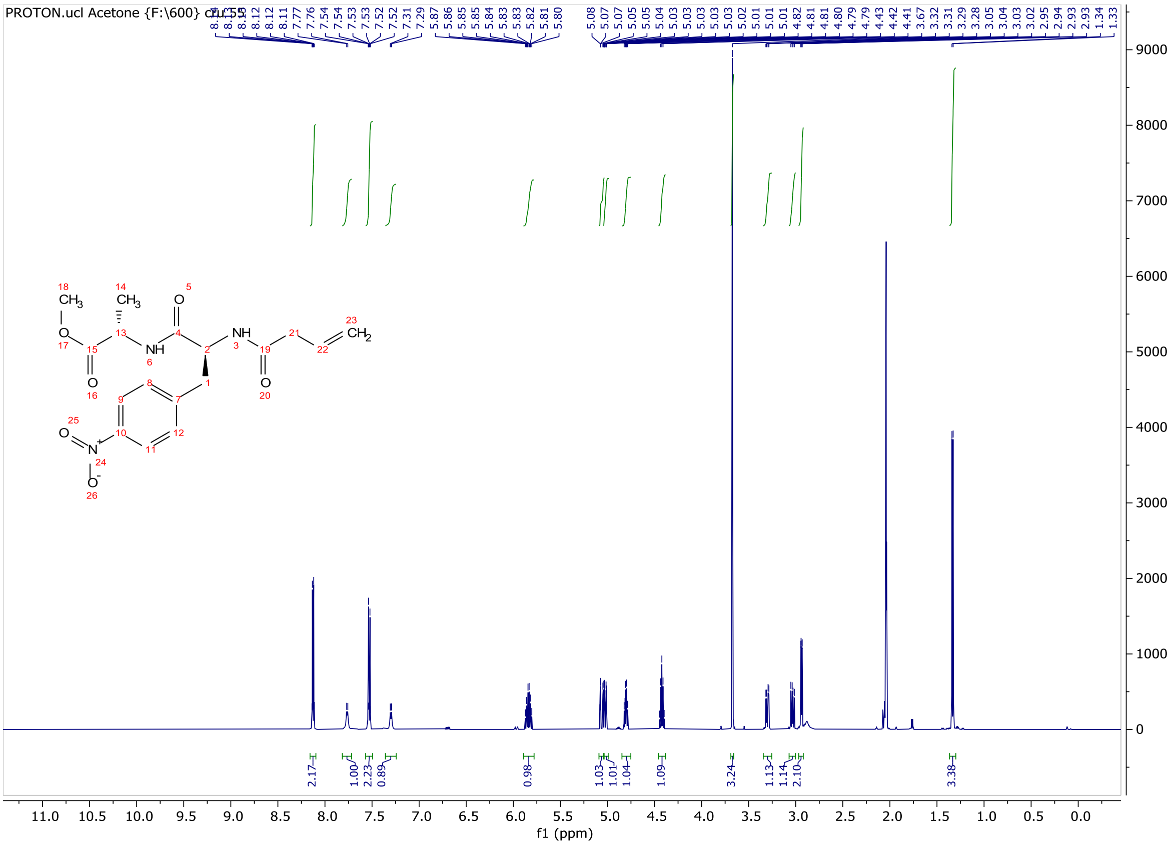
PROTON.ucl Acetone {F:\600} cru.55
f1 (ppm)

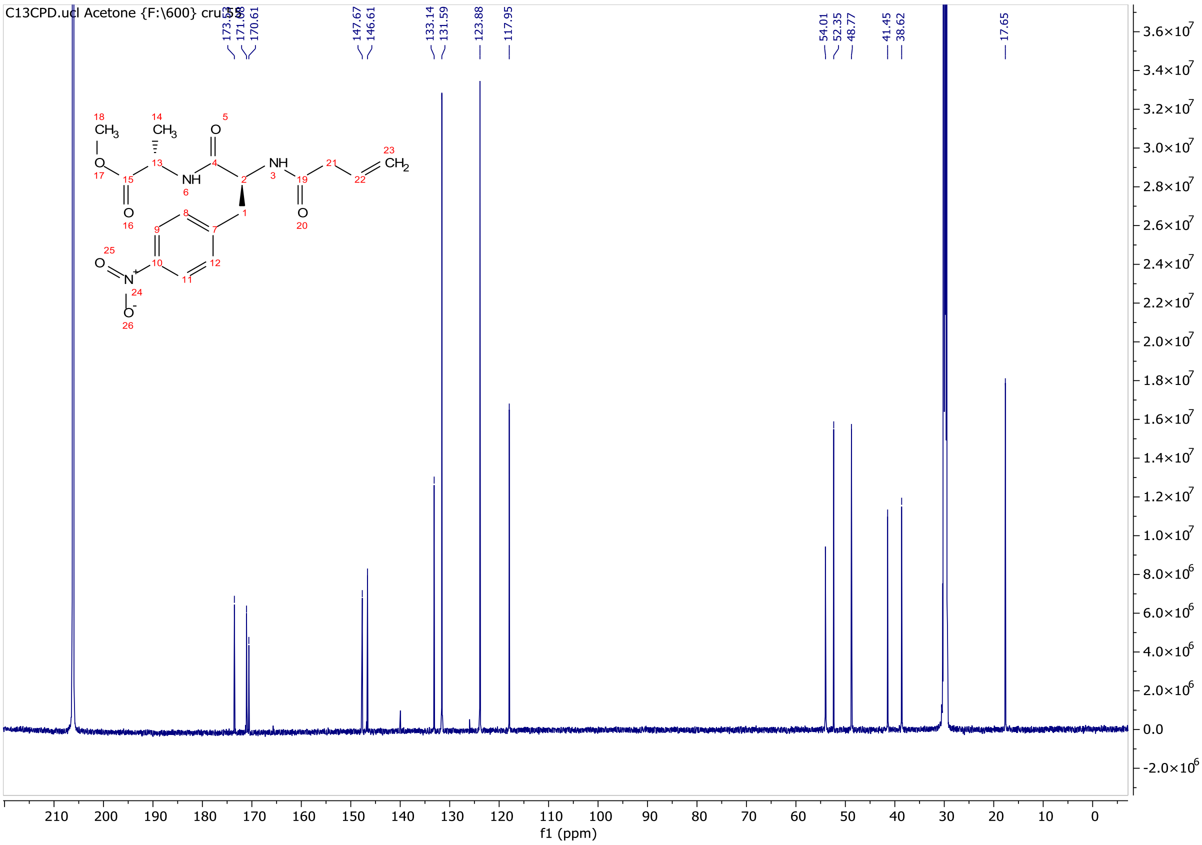
C13CPD.ucl Acetone {F:\600} cru.55
173.3
171.0
170.61
147.67
146.61
133.14
131.59
123.88
117.95
54.01
52.35
48.77
41.45
38.62
17.65
f1 (ppm)

((S)-2-(but-3-enamido)-3-(4-nitrophenyl)propanoyl)-L-alanine (6)

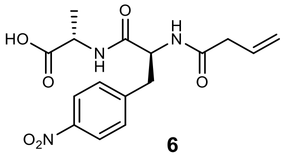

6

Methyl ester **s13** (164 mg, 0.45 mmol, 1.00 eq) was dissolved in 2M HCl in 1:1 dioxane:H_2O (4 mL). The reaction was stirred for 24 h at 25°C after which completion was observed by LC-MS. The solution was concentrated under reduced pressure. Purification by flash chromatography (C18, 10-100% MeCN/H_2O, 0.1% FA) afforded free acid **6** as a white solid (104 mg, 0.30 mmol, 66% yield).

^{1}H NMR (600 MHz, CD_3OD) δ 8.18 (d, *J* = 8.7 Hz, 2H), 7.54 (d, *J* = 8.8 Hz, 2H), 5.83 (ddt, *J* = 17.1, 10.2, 7.0 Hz, 1H), 5.13 (dq, *J* = 17.0, 1.5 Hz, 1H), 5.11 (dq, *J* = 10.0, 1.4 Hz, 1H), 4.78 (dd, *J* = 9.0, 5.5 Hz, 1H), 4.42 (q, *J* = 7.3 Hz, 1H), 3.33 (dd, *J* = 13.8, 5.4 Hz, 1H), 3.05 (dd, *J* = 13.9, 9.1 Hz, 1H), 2.98 (dd, *J* = 6.9, 1.5 Hz, 2H), 1.44 (d, *J* = 7.3 Hz, 3H).

^{13}C NMR (151 MHz, CD_3OD) δ 175.89, 173.80, 172.90, 148.62, 146.74, 132.88, 131.95, 124.66, 119.10, 55.24, 41.92, 39.15, 17.90.

HRMS (ESI+): calcd. for [$C_{16}H_{19}N_3O_6$+H^+] (MH^+) 350.1347; found 350.1347.

LC-MS trace (254 nM)

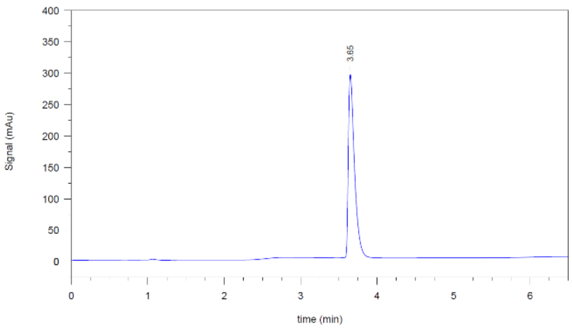

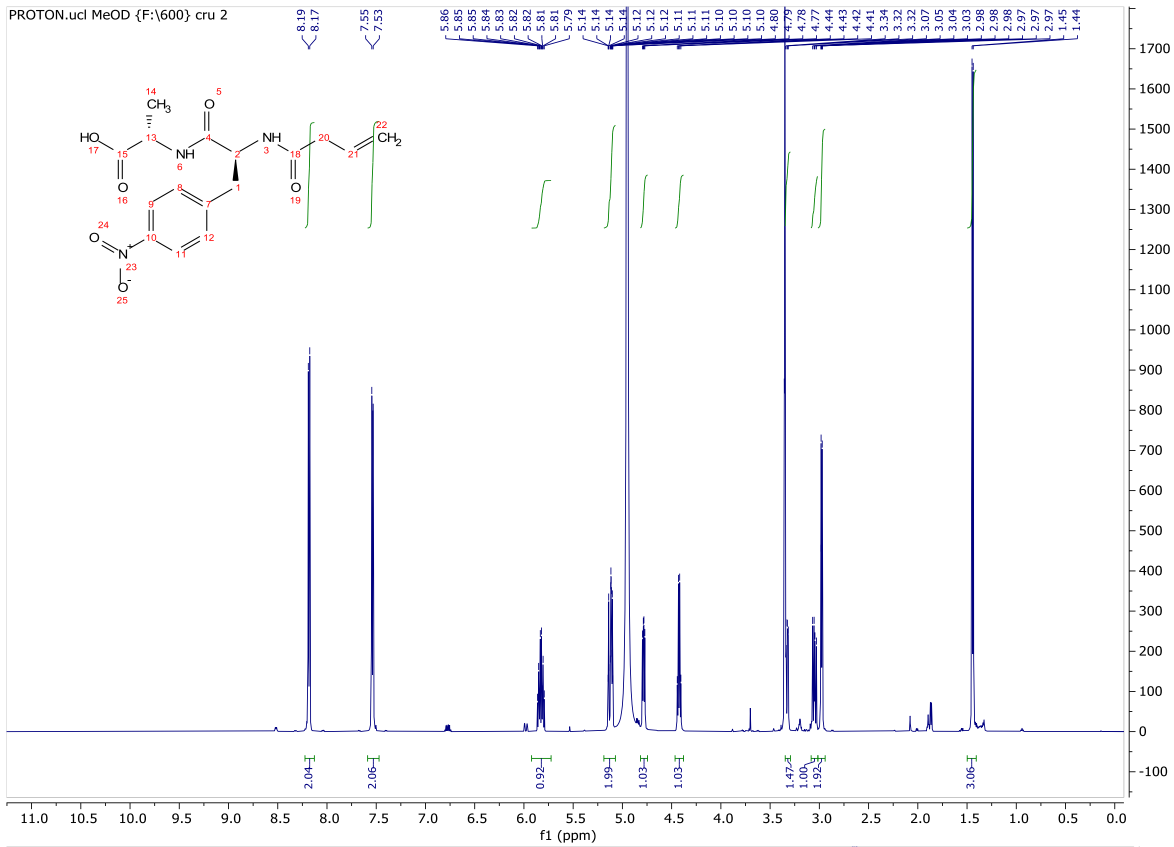
PROTON.ucl MeOD {F:\600} cru 2
f1 (ppm)

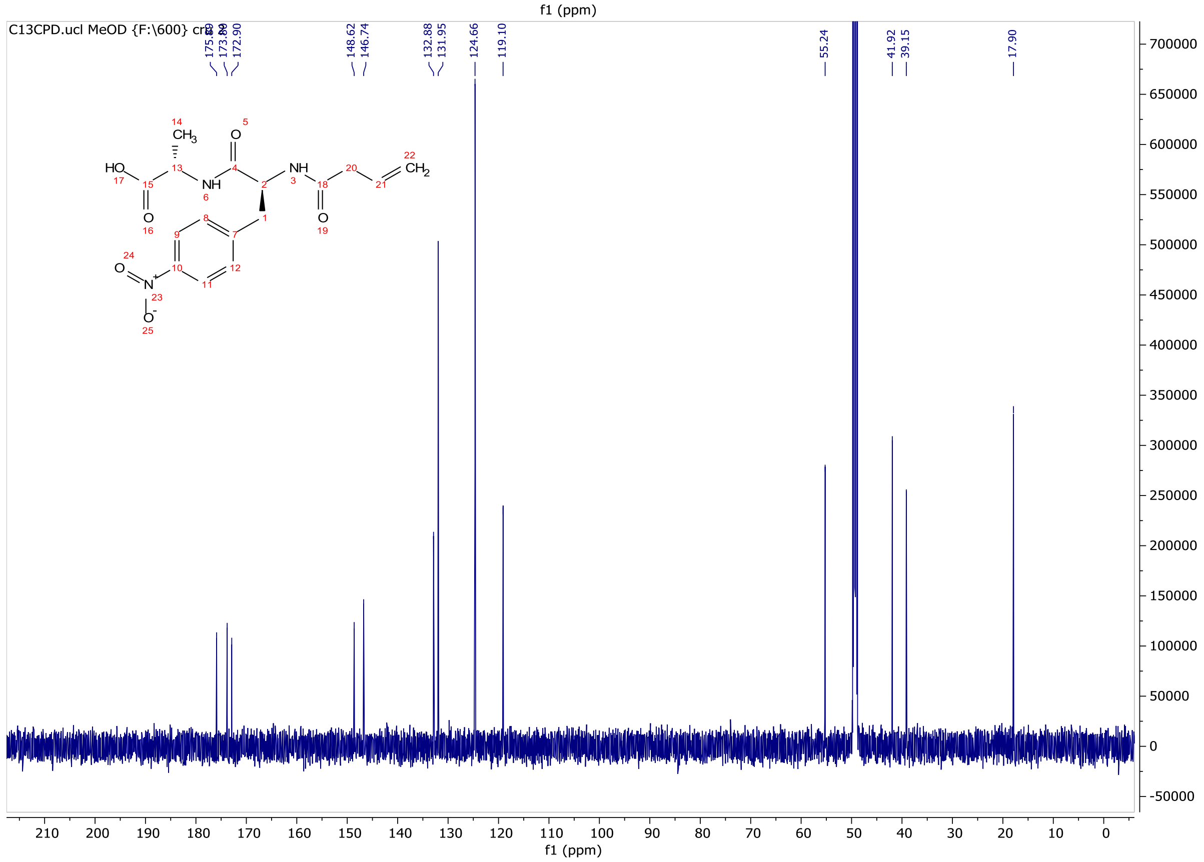
C13CPD.ucl MeOD {F:\600} cru 2
f1 (ppm)

(R)-1-(7-bromoquinolin-2-yl)ethyl (S)-1-(((S)-2-(but-3-enamido)-3-(4-nitrophenyl)propanoyl)-L-alanyl)hexahydropyridazine-3-carboxylate (7a)

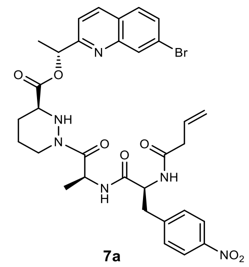

7a

Boc-protected piperazic ester **s9** (390 mg, 0.69 mmol, 1.00 eq) was dissolved in 1:1 TFA:CH_2Cl_2 (10 mL) under N_2. The clear solution was stirred protected from light for 1 h at 25°C. To avoid reactions with TFA observed before, the reaction was diluted with MeCN (5 mL), and the solvents were removed under reduced pressure several times at 30°C to yield a transparent oil. Avoiding exposure to air and light, the product was dissolved in dry MeCN (10 mL). *i*Pr_2NEt (400 µL, 2.30 mmol, 3.32 eq) was added along with peptide **6** (269 mg, 0.77 mmol, 1.11 eq), OxymaPure (108 mg, 0.76 mmol, 1.10 eq) and HATU (394 mg, 1.04 mmol, 1.50 eq). The clear brown solution was stirred for 12 h at 25°C after which completion was observed by LC-MS. The reaction was diluted with EtOAc and 0.5 M aq. HCl. The layers were separated, and the organic layer was washed with 1M aq. NaOH (1x) and brine (1x). The aqueous layer was extracted with EtOAc (1x) at each step. The combined organic layers were dried over $MgSO_4$ and concentrated under reduced pressure to yield a yellow oil (540 mg). Purification by flash chromatography (C18, 20-100% MeCN/H_2O, 0.1% TFA then silica, 0-5% MeOH/CH_2Cl_2) afforded **7a** as a yellow solid (265 mg, 0.38 mmol, 55% yield).

R_f = 0.24 in 5% MeOH/CH_2Cl_2.

^{1}H NMR (500 MHz, CD_3OD) δ 8.39 (d, *J* = 8.4 Hz, 1H), 8.24 (d, *J* = 2.0 Hz, 1H), 8.13 (d, *J* = 8.7 Hz, 2H), 7.89 (d, *J* = 8.7 Hz, 1H), 7.73 (dd, *J* = 8.7, 1.9 Hz, 1H), 7.63 (d, *J* = 8.6 Hz, 1H), 7.44 (d, *J* = 8.7 Hz, 2H), 6.10 (q, *J* = 6.7 Hz, 1H), 5.81 (ddt, *J* = 17.1, 10.1, 6.9 Hz, 1H), 5.37 (q, *J* = 6.9 Hz, 1H), 5.12 (dq, *J* = 17.1, 1.6 Hz, 1H), 5.09 (dq, *J* = 10.1, 1.5 Hz, 1H), 4.70 (dd, *J* = 9.0, 5.6 Hz, 1H), 3.93 – 3.86 (m, 1H), 3.80 (dd, *J* = 8.4, 4.1 Hz, 1H), 3.19 (dd, *J* = 13.9, 5.5 Hz, 1H), 3.01 – 2.90 (m, 1H), 2.95 (dt, *J* = 6.9, 1.5 Hz, 3H), 2.11 (ddd, *J* = 10.0, 6.5, 3.0 Hz, 1H), 2.04 – 1.81 (m, 2H), 1.83 – 1.73 (m, 1H), 1.71 (d, *J* = 6.7 Hz, 3H), 1.34 (d, *J* = 6.9 Hz, 3H).

^{13}C NMR (126 MHz, CD_3OD) δ 175.52, 173.74, 172.29, 172.08, 163.56, 149.29, 148.57, 146.81, 139.40, 132.84, 132.00, 131.85, 131.61, 130.95, 128.02, 125.40, 124.66, 120.06, 119.13, 75.57, 55.43, 47.74, 42.93, 41.94, 39.03, 28.80, 23.67, 21.28, 18.67 (1 signal likely under solvent).

HRMS (ESI+): calcd. for $[C_{32}H_{35}N_6O_7Br+H]^+$ (MH^+) 695.1823; found 695.1821.

LC trace (254 nM)

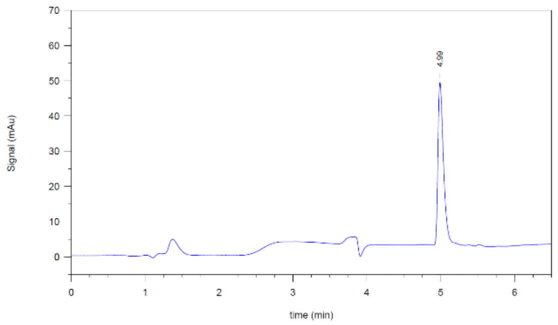

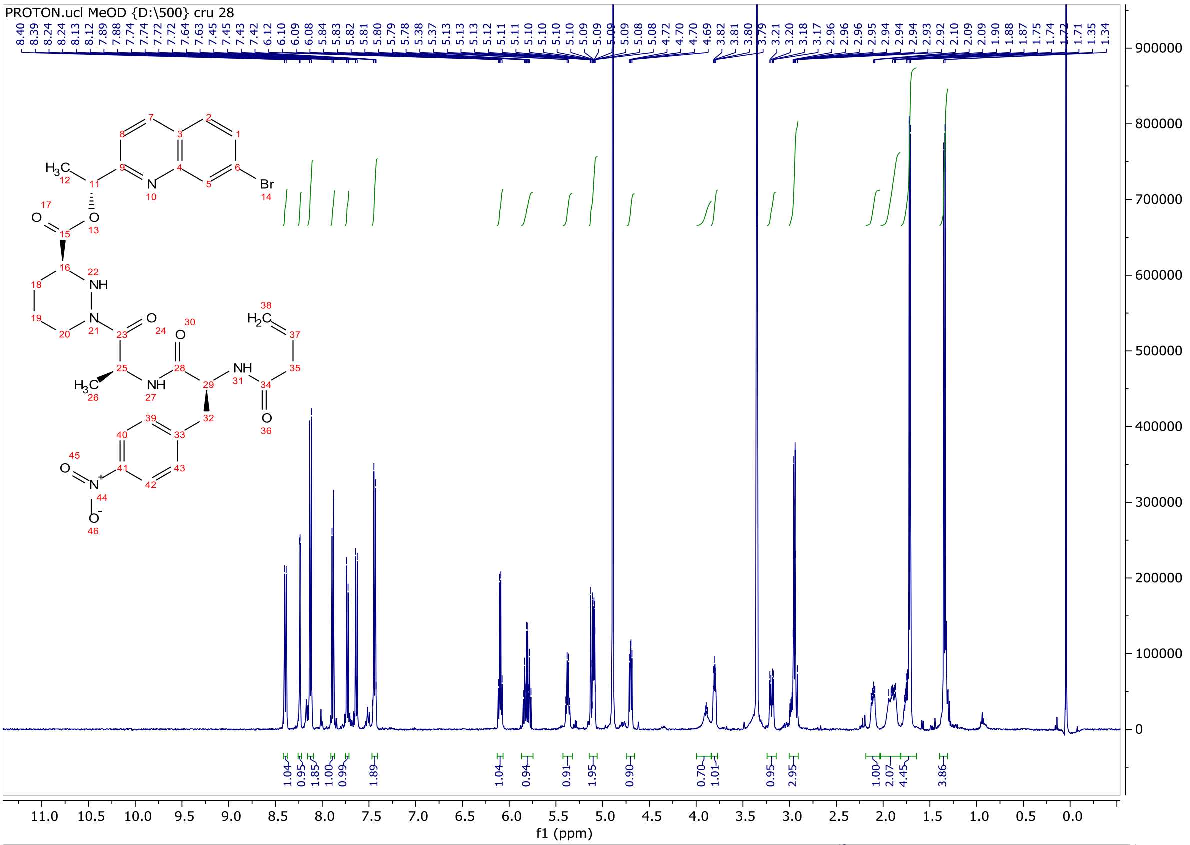
PROTON.ucl MeOD {D:\500} cru 28
f1 (ppm)

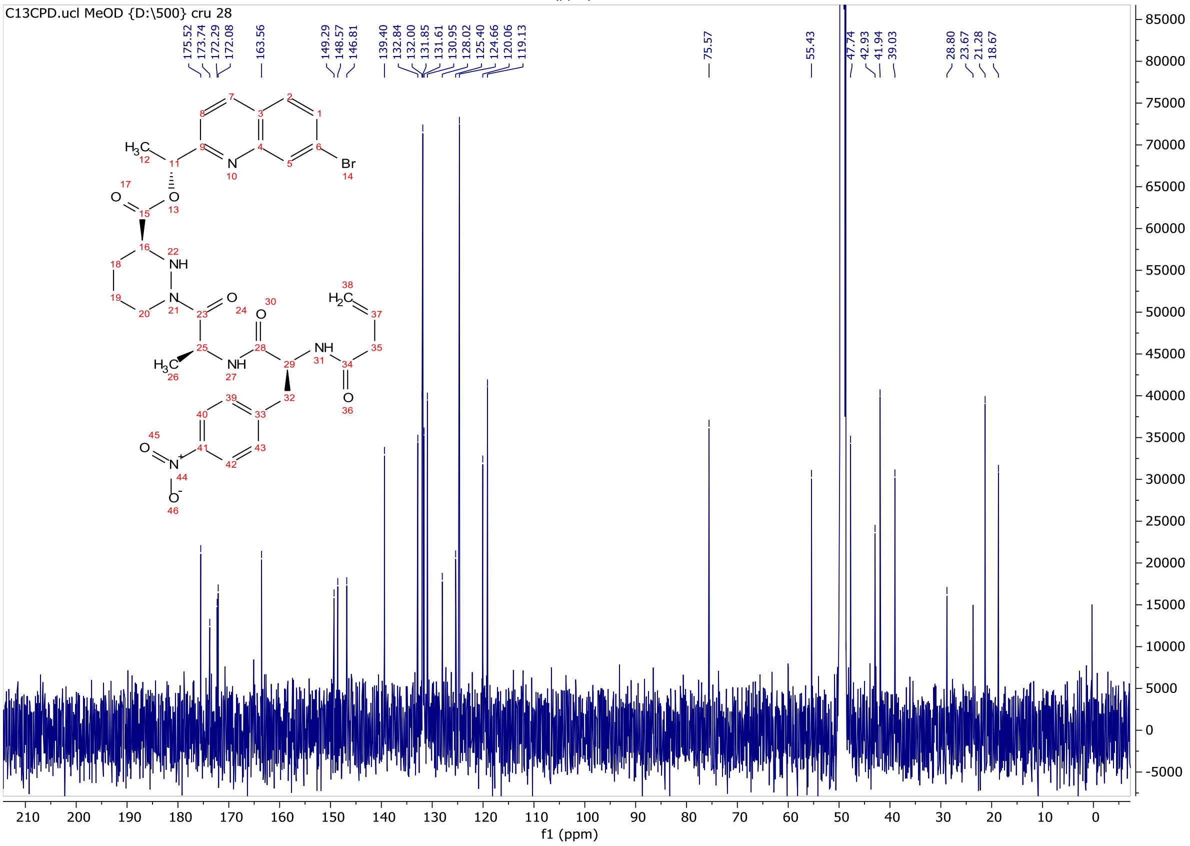
C13CPD.ucl MeOD {D:\500} cru 28
175.52
173.74
172.29
172.08
163.56
149.29
148.57
146.81
139.40
132.84
132.00
131.85
131.61
130.95
128.02
125.40
124.66
120.06
119.13
75.57
55.43
47.74
42.93
41.94
39.03
28.80
23.67
21.28
18.67
f1 (ppm)

(R)-1-(7-bromoquinolin-2-yl)hex-5-yn-1-yl (S)-1-(((S)-2-(but-3-enamido)-3-(4-nitrophenyl) propanoyl)-L-alanyl)hexahydropyridazine-3-carboxylate (7b)

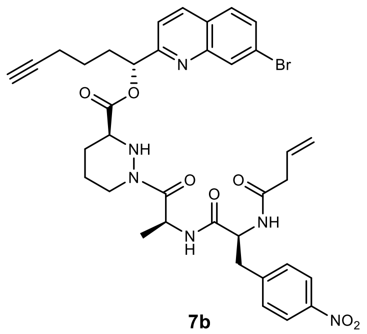

7b

TMS- and bis Boc-protected alkyne piperazic ester **s10** (404 mg, 0.59 mmol, 1.00 eq) was dissolved in 1:1 TFA:CH_2Cl_2 (9 mL) under N_2. The clear solution was stirred protected from light for 2 h at 25°C. To avoid reactions with TFA observed previously, the reaction was diluted with MeCN (5 mL), and the solvents were removed under reduced pressure several times at 30°C to yield a transparent oil. Avoiding exposure to air and light, the product was dissolved in dry MeCN (5 mL) and *i*Pr_2NEt (256 µL, 1.47 mmol, 2.50 eq) was added. This solution was then added to a solution of peptide **6** (205 mg, 0.59 mmol, 1.00 eq), OxymaPure (83.4 mg, 0.59 mmol, 1.00 eq), HATU (268 mg, 0.70 mmol, 1.20 eq) in dry MeCN (10 mL). The clear brown solution was stirred for 2 h at 25°C after which completion was observed by LC-MS. The reaction was diluted with EtOAc and 1M aq. HCl. The layers were separated, and the organic layer was washed with 2M aq. NaOH (1x) and brine (1x). The aqueous layer was extracted with EtOAc (1x) at each step. The combined organic layers were dried over $MgSO_4$ and concentrated under reduced pressure to yield a yellow oil (464 mg). Purification by flash chromatography (C18, 0-100% MeCN/H_2O, 0.1% TFA then silica, 0-5% MeOH/CH_2Cl_2) afforded **7b** as a grey solid (165 mg, 0.22 mmol 38% yield).

R_f = 0.46 in 5% MeOH/CH_2Cl_2.

^{1}H NMR (600 MHz, $CDCl_3$) δ 8.27 (d, *J* = 1.8 Hz, 1H), 8.19 (d, *J* = 8.4 Hz, 1H), 8.08 (d, *J* = 8.2 Hz, 2H), 7.70 (d, *J* = 8.6 Hz, 1H), 7.65 (dt, *J* = 8.6, 1.5 Hz, 1H), 7.44 (d, *J* = 8.4 Hz, 1H), 7.30 (d, *J* = 8.3 Hz, 2H), 6.59 (d, *J* = 7.4 Hz, 1NH), 6.36 (d, *J* = 7.7 Hz, 1 NH), 6.02 (dd, *J* = 7.6, 5.9 Hz, 1H), 5.86 (ddt, *J* = 17.3, 10.3, 7.1 Hz, 1H), 5.22 (dt, *J* = 10.1, 1.3 Hz, 1H), 5.19 (dq, *J* = 17.0, 1.5 Hz, 1H), 5.14 (t, *J* = 6.9 Hz, 1H), 4.67 (q, *J* = 6.9 Hz, 1H),4.27 (br s, 1 NH), 3.86 (d, *J* = 11.1 Hz, 1H), 3.65 (s, 1H), 3.11 (d, *J* = 6.5 Hz, 2H), 2.99 (dd, *J* = 7.4, 1.4 Hz, 2H), 2.95 – 2.86 (m, 1H), 2.27 (td, *J* = 7.1, 2.6 Hz, 2H), 2.24 – 2.14 (m, 3H), 1.98 (t, *J* = 2.7 Hz, 1H), 1.93 (dt, *J* = 12.9, 3.4 Hz, 1H), 1.84 – 1.54 (m, 4H), 1.25 (d, *J* = 6.8 Hz, 3H).

^{13}C NMR (151 MHz, $CDCl_3$) δ 172.90, 170.36, 170.25, 168.70, 159.51, 147.96, 146.98, 144.05, 137.35, 131.51, 130.62, 130.40, 130.35, 130.31, 128.87, 126.19, 124.17, 123.59, 120.09, 118.79, 83.44, 69.14, 58.75, 53.78, 46.14, 41.80, 41.35, 38.65, 33.58, 28.60, 24.29, 22.65, 18.76, 18.13.

HRMS (ESI+): calcd. for $[C_{36}H_{39}N_6O_7+H]^+$ (MH^+) 747.2137; found 747.2130.

LC trace (254 nM)

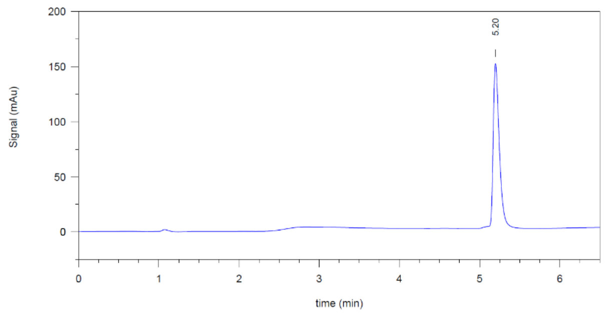

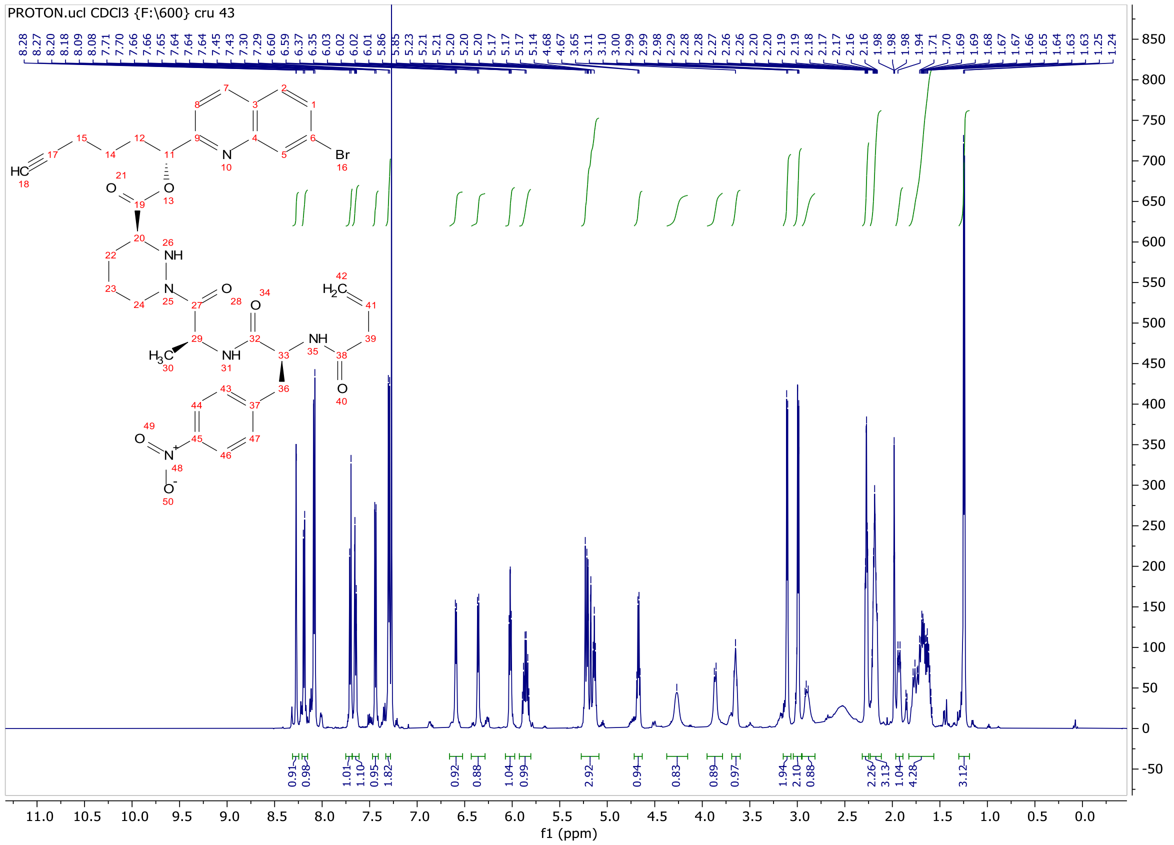

PROTON.ucl CDCl3 {F:\600} cru 43
f1 (ppm)

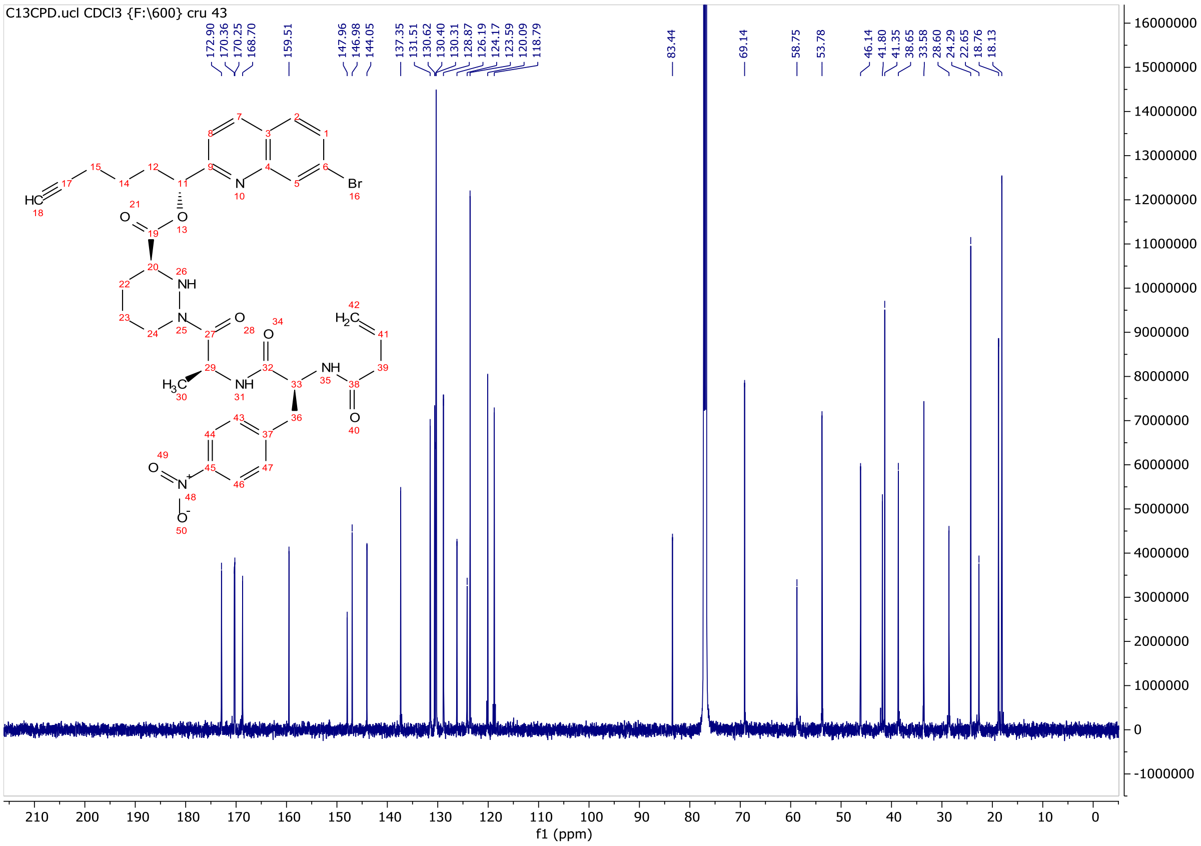

C13CPD.ucl CDCl3 {F:\600} cru 43
172.90 170.36 170.25 168.70 159.51 147.96 146.98 144.05 137.35 131.51 130.62 130.40 130.31 128.87 126.19 124.17 123.59 120.09 118.79 83.44 69.14 58.75 53.78 46.14 41.80 41.35 38.65 33.58 28.60 24.29 22.65 18.76 18.13
f1 (ppm)

(R)-1-(7-bromoquinolin-2-yl)ethyl (S)-1-((S)-2-((tert-butoxycarbonyl)amino)pent-4-ynoyl)hexahydropyridazine-3-carboxylate (s14)

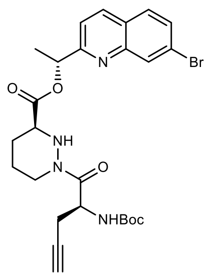

s14

Boc-protected (3S)-piperazic acid **4** (359 mg, 0.64 mmol, 1.00 eq) was dissolved in 4M HCl in dioxane (10 mL) and stirred for 1.5 h after which completion was visible by LC-MS. The solvents were removed under reduced pressure and the crude residue was dissolved in dry MeCN (2 mL). N-Boc-propargyl glycine (142 mg, 0.67 mmol, 1.05 eq), OxymaPure (95 mg, 0.67 mmol, 1.05 eq), HATU (254 mg, 0.67 mmol, 1.05 eq) and *i*Pr_2NEt (443 µL, 2.54 mmol, 4.00 eq) were added and the reaction was stirred at 25°C for 15 h. The reaction was diluted with EtOAc, then washed with H_2O (1x) and brine (1x). The organic layer was dried over $MgSO_4$ and concentrated under reduced pressure. Purification by flash chromatography (C18, 10-90% MeCN/H_2O, 0.1% TFA) afforded the coupled amide **s14** as a yellow foam (TFA salt, 233 mg, 0.35 mmol, 54% yield).

^{1}H NMR (600 MHz, Acetone-d6) δ 8.43 (d, *J* = 8.6 Hz, 1H), 8.26 (d, *J* = 2.0 Hz, 1H), 7.92 (d, *J* = 8.6 Hz, 1H), 7.72 (dd, *J* = 8.7, 2.0 Hz, 1H), 7.67 (d, *J* = 8.6 Hz, 1H), 6.09 (q, *J* = 6.9 Hz, 1H), 5.20 (br t, *J* = 5.3 Hz, 1H), 3.87 (dd, *J* = 8.9, 3.9 Hz, 1H), 4.09 – 3.74 (m, 1H), 3.32 (br s, 1H), 2.68 (dd, *J* = 5.5, 2.7 Hz, 2H), 2.39 (t, *J* = 2.7 Hz, 1H), 2.13 – 2.07 (m, 1H), 1.94 – 1.79 (m, 2H), 1.68 (d, *J* = 6.6 Hz, 3H), 1.69 – 1.63 (m, 1H), 1.36 (m, 9H). (rotamers increase multiplicity of some peaks near Boc group).

^{13}C NMR (151 MHz, Acetone-d6) δ 171.48, 171.19, 162.49, 158.71 (q TFA), 155.37, 148.20, 138.68, 131.53, 130.77, 130.47, 127.14, 124.31, 119.55, 116.02 (q TFA), 80.84, 79.04, 74.40, 72.03, 59.17 (br), 50.15, 42.04, 28.42, 28.23 (br), 23.16, 23.13 (br), 20.64. broadening due to rotamers.

LCMS calcd. for $[C_{26}H_{31}BrN_4O_5+H]^+$ (MH^+) 559.15; found 559.2

LC trace (254 nM)

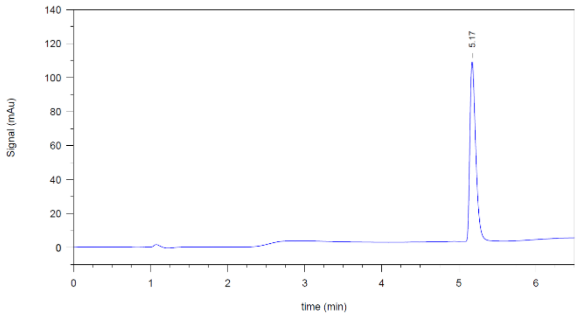

PROTON.ucl Acetone {F:\600} cru 27

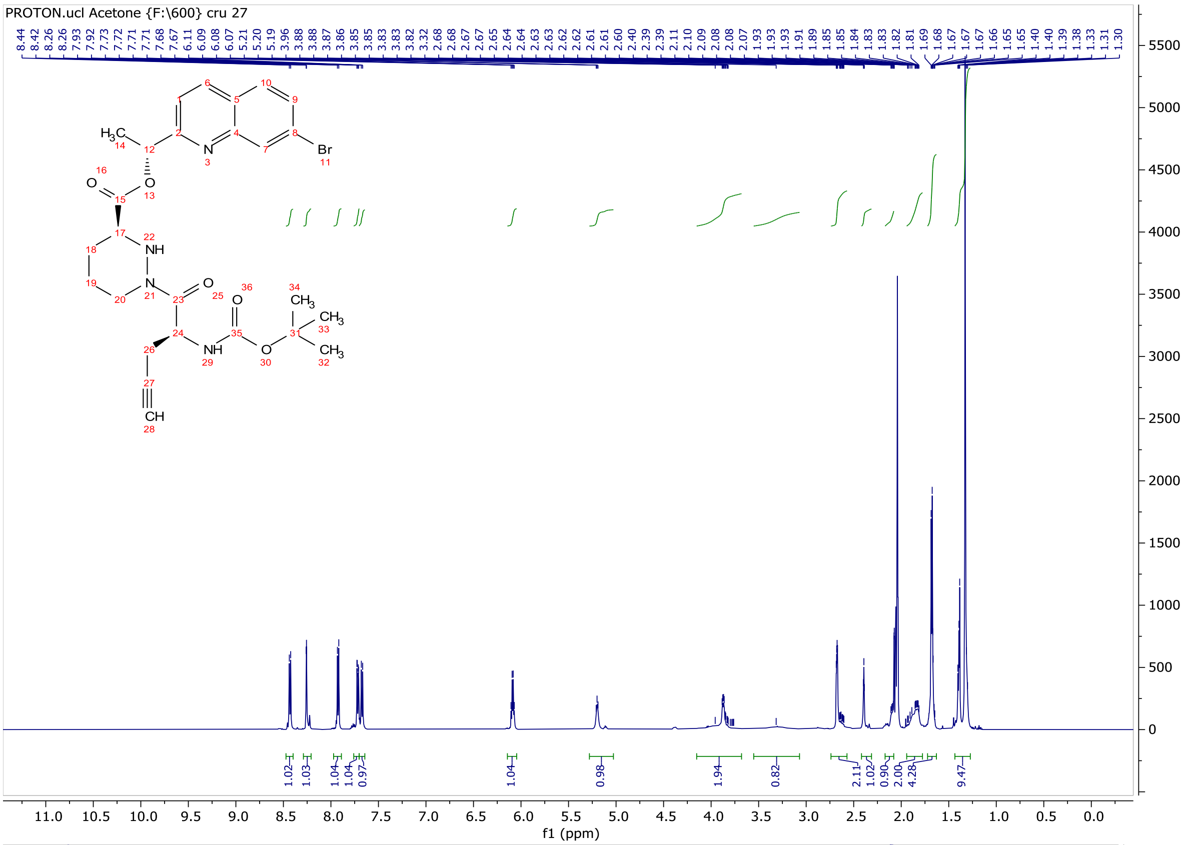

C13CPD.ucl Acetone {F:\600} cru 27

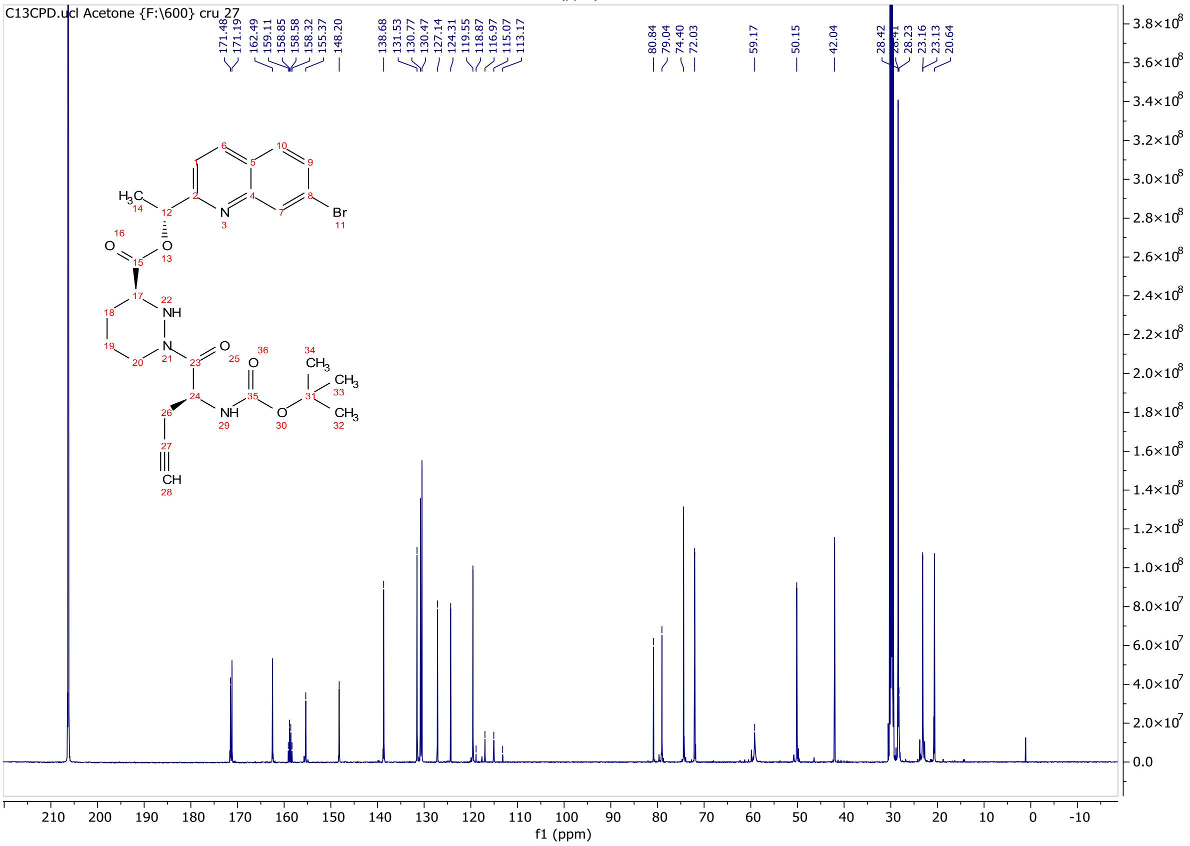

(R)-1-(7-bromoquinolin-2-yl)ethyl (S)-1-((S)-2-((S)-2-((tert-butoxycarbonyl)amino)-3-(4-nitrophenyl)propanamido)pent-4-ynoyl)hexahydropyridazine-3-carboxylate (s15)

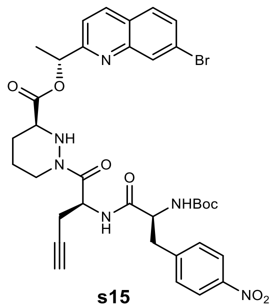

s15

Boc-protected amine **s14** (233 mg, 0.42 mmol, 1.00 eq) was dissolved in 1:1 CH_2Cl_2:TFA (5 mL) and was stirred for 1h, after which completion was observed by LC-MS. The solvents were removed under reduced pressure and the residue dissolved in dry MeCN (5 mL). To this solution was added *i*Pr_2NEt (117 µL, 0.83 mmol, 4.00 eq), N-Boc-(4-nitro)phenylalanine (136 mg, 0.44 mmol, 1.05 eq), OxymaPure (62 mg, 0.44 mmol, 1.05 eq) and HATU (166 mg, 0.44 mmol, 1.05 eq). The reaction was stirred for 15 h at 25°C, diluted with EtOAc, then washed with H_2O (1x) and brine (1x). The organic layer was dried over $MgSO_4$ and concentrated under reduced pressure. Purification by flash chromatography (C18, 0-100% MeCN/H_2O, 0.1% TFA) provided **s15** as an orange oil (TFA salt, 247 mg, 0.29 mmol, 69% yield).

^{1}H NMR (600 MHz, Acetone-d6) δ 8.43 (d, *J* = 8.5 Hz, 1H), 8.23 (d, *J* = 2.0 Hz, 1H), 8.10 (d, *J* = 8.3 Hz, 2H), 7.92 (d, *J* = 8.7 Hz, 1H), 7.71 (dd, *J* = 8.7, 2.0 Hz, 1H), 7.68 (d, *J* = 8.5 Hz, 1H), 7.50 (d, *J* = 8.4 Hz, 2H), 6.09 (q, *J* = 6.8 Hz, 1H), 5.46 (s, 1H), 4.47 (dd, *J* = 9.3, 5.1 Hz, 1H), 4.13 – 3.68 (br m, 1H), 3.90 (dd, *J* = 8.8, 4.1 Hz, 1H), 3.58 – 3.13 (br m, 1H), 3.25 (dd, *J* = 14.0, 5.0 Hz, 1H), 3.01 (dd, *J* = 13.8, 9.3 Hz, 1H), 2.76 – 2.64 (m, 2H), 2.40 (t, *J* = 2.7 Hz, 1H), 2.16-2.08 (m, 1H), 1.97 – 1.81 (m, 2H), 1.75 – 1.63 (m, 1H), 1.67 (d, *J* = 6.8 Hz, 3H), 1.75 – 1.63 (m, 1H), 1.30 (s, 9H).

^{13}C NMR (151 MHz, Acetone-d6) δ 171.23, 170.95, 170.91, 162.48, 158.58 (q, TFA), 155.98, 148.27, 147.55, 147.03, 138.67, 131.59, 131.46, 131.41, 130.78, 130.49, 127.15, 124.32, 123.89, 119.58, 115.94 (q, TFA), 80.63, 79.49, 74.44, 72.21, 59.12 (br), 56.02, 49.21, 42.05, 38.60, 28.38, 28.24 (br), 23.09 (br), 22.74, 20.65. Some signals are broadened due to rotamers.

LCMS calcd. for $[C_{35}H_{39}BrN_6O_8+H]^+$ (MH^+) 751.20; found 751.3

LC trace (254 nM)

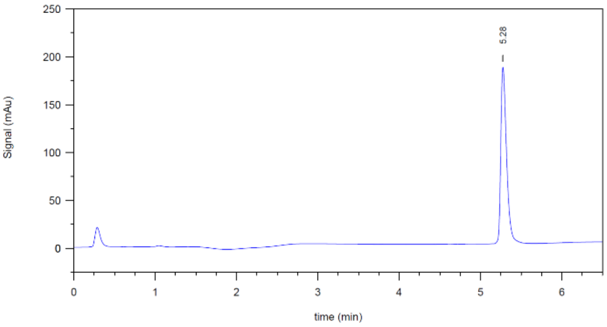

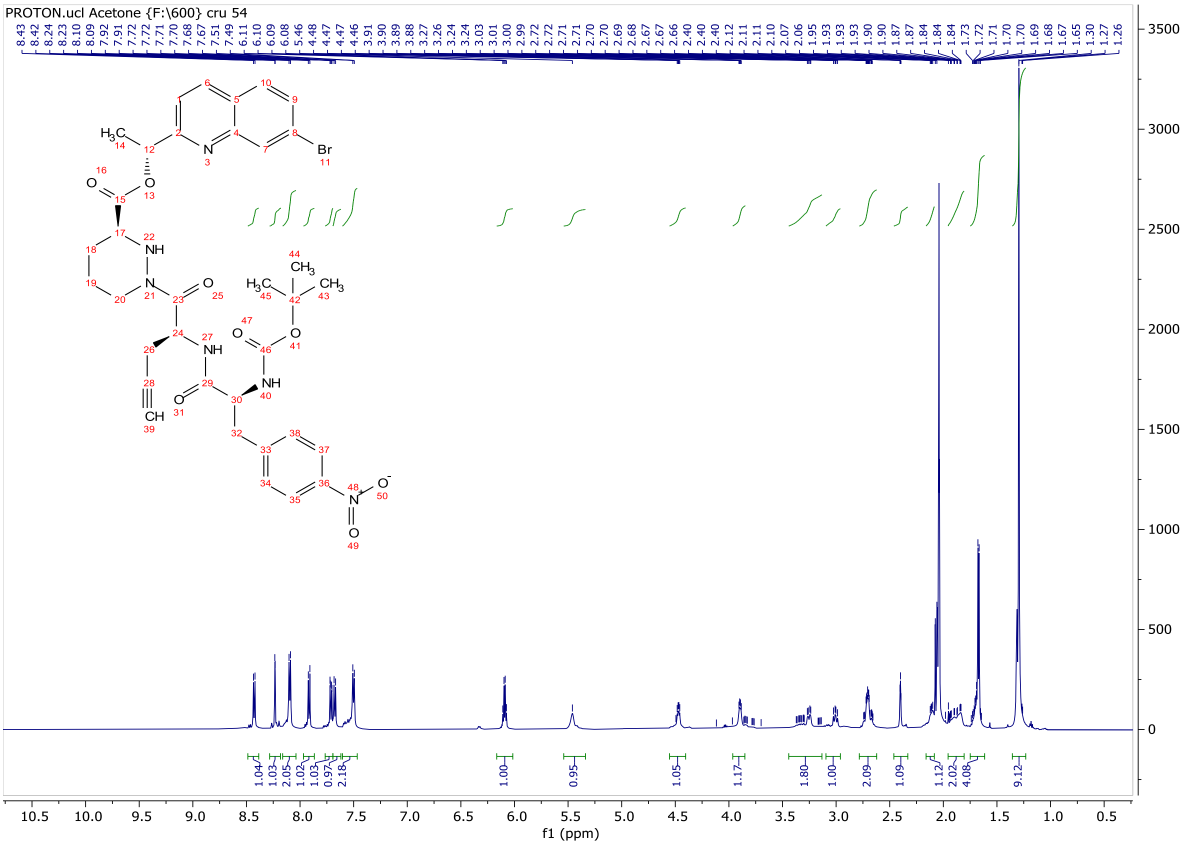
PROTON.ucl Acetone {F:\600} cru 54
f1 (ppm)

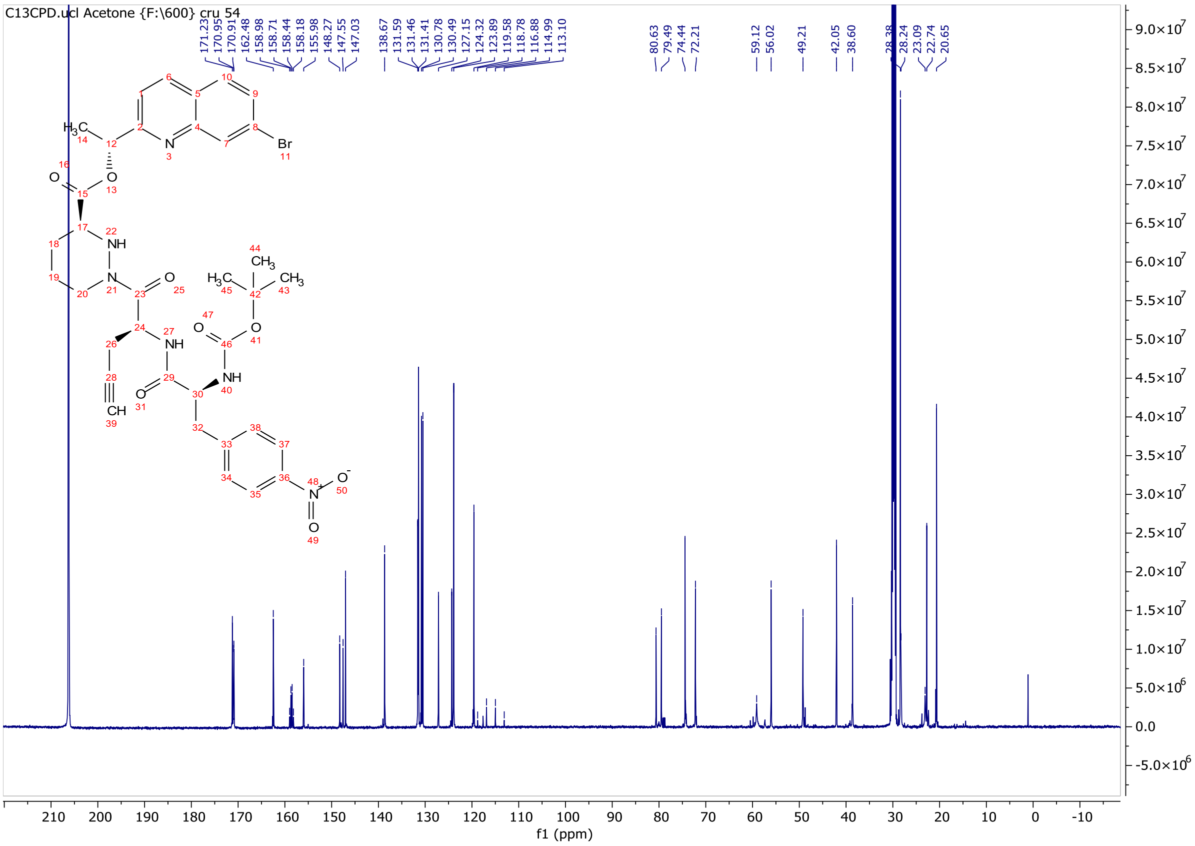
C13CPD.ucl Acetone {F:\600} cru 54
f1 (ppm)

(R)-1-(7-bromoquinolin-2-yl)ethyl (S)-1-((S)-2-((S)-2-(but-3-enamido)-3-(4-nitrophenyl) propanamido)pent-4-ynoyl)hexahydropyridazine-3-carboxylate (7c)

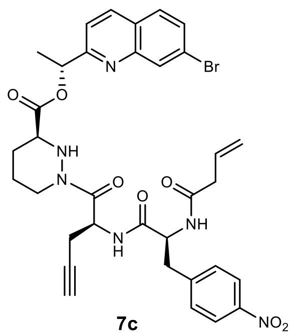

7c

Boc-protected amine **s15** (247 mg, 0.33 mmol, 1.00 eq) was dissolved in 1:1 TFA:CH_2Cl_2 (2 mL) and stirred for 1.5 h at 25°C, after which completion was observed by LC-MS. The reaction was concentrated under reduced pressure and the residue dissolved in dry MeCN (4 mL). To the reaction was added 3-butenoic acid (29 µL, 0.34 mmol, 1.05 eq), PyAOP (131 mg, 0.34 mmol, 1.05 eq), *i*Pr_2NEt (229 µL, 1.31 mmol, 4.00 eq) and the reaction was stirred at 25°C for 15 h. The reaction was diluted with EtOAc, then washed with 1M aq. HCl (2x) and brine (1x). The organic layer was dried over $MgSO_4$ and concentrated under reduced pressure. The residue was purified by flash chromatography (C18, 10-100% MeCN/H_2O, 0.1% TFA) and dissolved in EtOAc. The solution was washed with 1M HCl (5x, to remove residual phosphine species), dried over $MgSO_4$ and concentrated under reduced pressure to afford **7c** as a light-yellow powder (HCl salt, 141 mg, 0.19 mmol, 57%).

^{1}H NMR (600 MHz, Acetone-d_6) δ 8.40 (d, *J* = 8.5 Hz, 1H), 8.23 (d, *J* = 2.0 Hz, 1H), 8.05 (d, *J* = 8.6 Hz, 2H), 7.90 (d, *J* = 8.7 Hz, 1H), 7.70 (dd, *J* = 8.7, 2.0 Hz, 1H), 7.66 (d, *J* = 8.6 Hz, 1H), 7.59 (d, *J* = 7.7 Hz, 1NH), 7.44 (d, *J* = 8.5 Hz, 2H), 7.39 (d, *J* = 8.3 Hz, 1NH), 6.09 (q, *J* = 6.7 Hz, 1H), 5.83 (ddtd, *J* = 17.2, 10.2, 7.0, 1.0 Hz, 1H), 5.67 (br s, 1 NH), 5.46 (s, 1H), 5.06 (dd, *J* = 17.2, 1.8 Hz, 1H), 5.01 (dd, *J* = 9.9, 1.8 Hz, 1H), 4.83 (dd, *J* = 13.9, 7.8 Hz, 1H), 4.03 – 3.67 (br m, 1H), 3.90 (t, *J* = 4.1 Hz, 1H), 3.59 – 3.24 (br m, 1H), 3.19 (dd, *J* = 13.7, 5.5 Hz, 1H), 3.03 – 2.92 (m, 1H), 2.94 (dt, *J* = 6.9, 1.3 Hz, 2H), 2.68 (dd, *J* = 5.7, 2.7 Hz, 2H), 2.40 (t, *J* = 2.7 Hz, 1H), 2.16 – 2.07 (m, 1H), 1.96 – a1.79 (m, 2H), 1.80 – 1.63 (m, 1H), 1.67 (d, *J* = 6.8 Hz, 3H).

^{13}C NMR (151 MHz, Acetone-d6) δ 171.30, 170.94, 170.61, 170.47, 162.63, 148.80, 147.63, 146.74, 138.21, 133.13, 132.03, 131.52, 130.67, 130.51, 127.17, 124.08, 123.93, 119.60, 118.09, 80.66, 74.66, 72.21, 59.30, 54.36, 49.36, 42.12, 41.52, 38.48, 28.37, 23.19, 22.69, 20.69.

HRMS (ESI+): calcd. for $[C_{34}H_{34}BrN_6O_7]^-$ $(M-H)^-$ 717.1678; found 717.1677.

LC trace (254 nM)

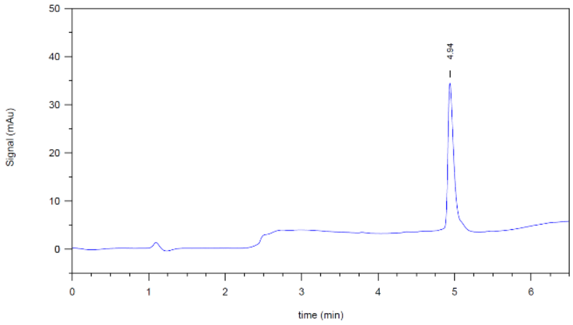

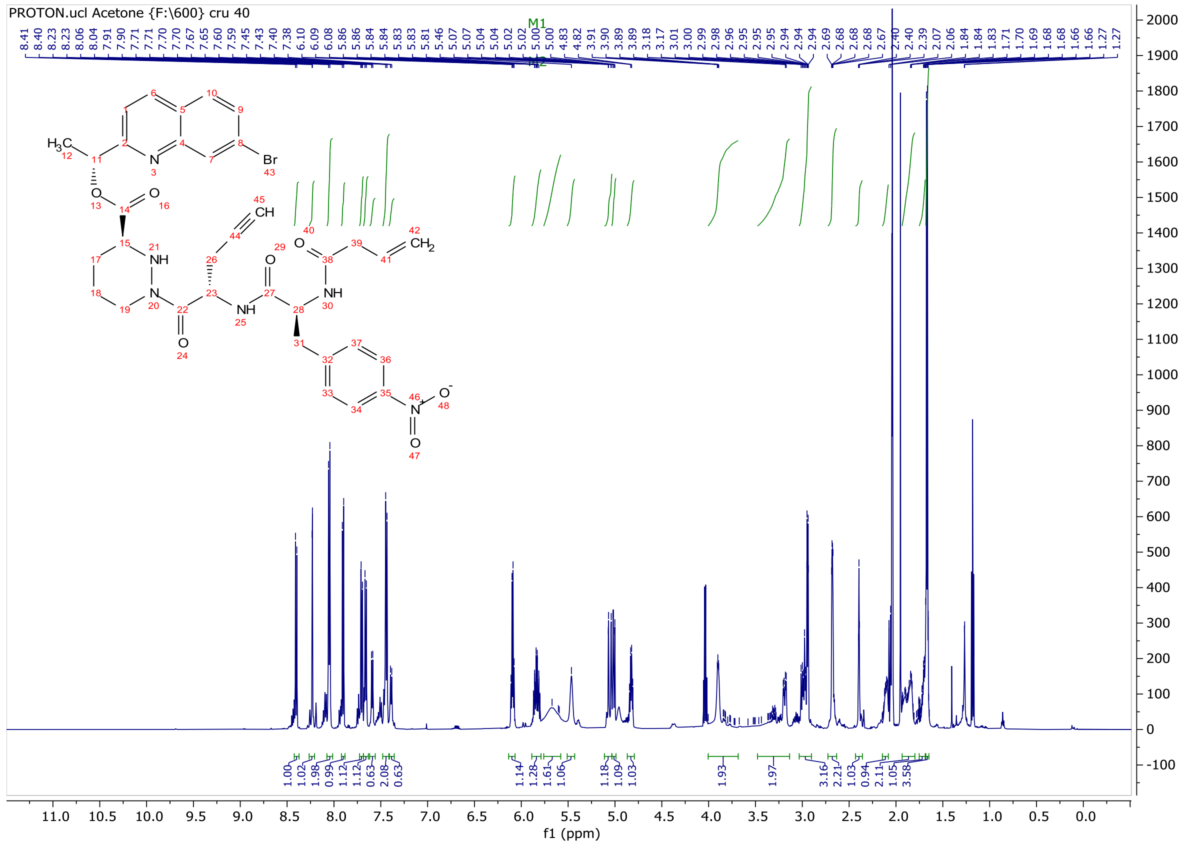
PROTON.ucl Acetone {F:\600} cru 40
f1 (ppm)

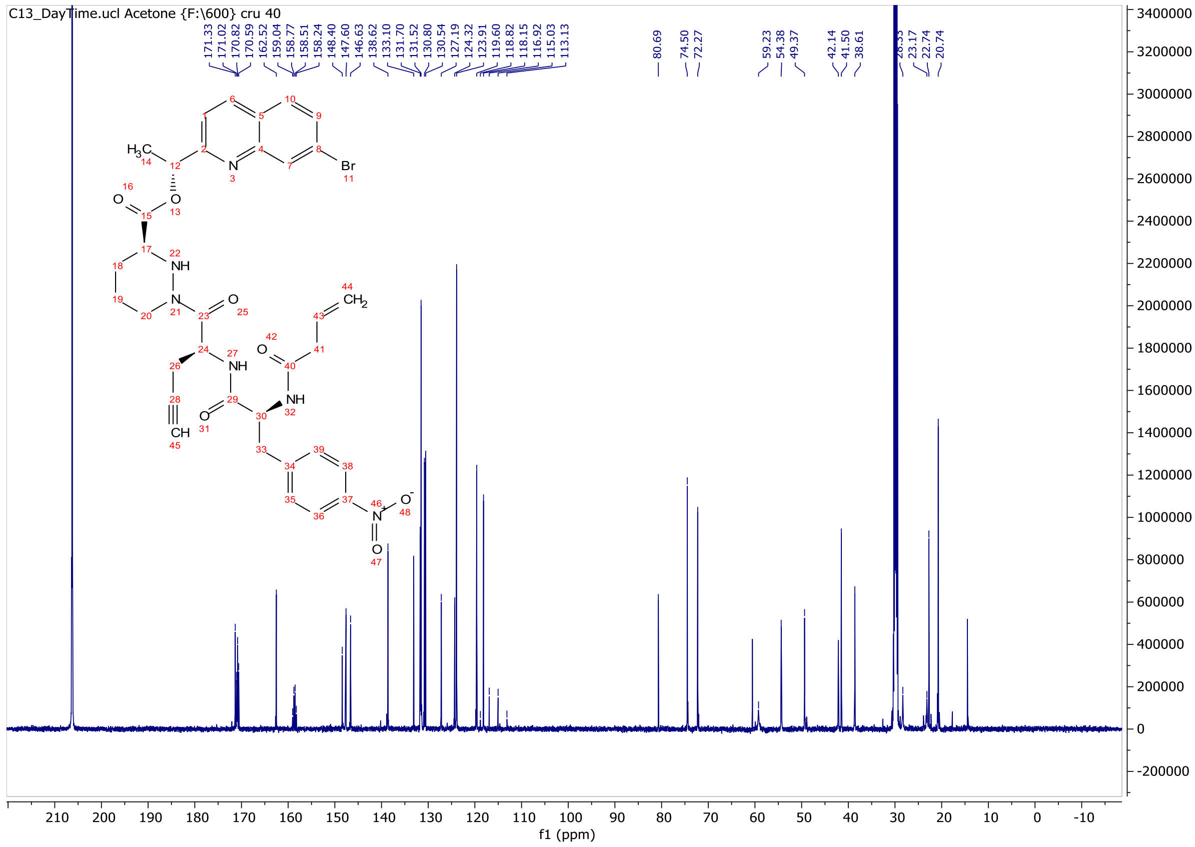
C13_DayTime.ucl Acetone {F:\600} cru 40
f1 (ppm)

6-(4-((R)-4-(7-bromoquinolin-2-yl)-4-(((S)-1-(((S)-2-(but-3-enamido)-3-(4-nitrophenyl)propanoyl)-L-alanyl)hexahydropyridazine-3-carbonyl)oxy)butyl)-1H-1,2,3-triazol-1-yl)hexanoic acid (s16)

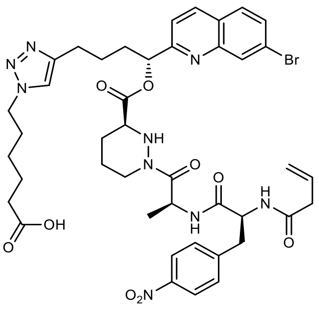

s16

Terminal alkyne **7b** (45.0 mg, 60 µmol, 1.00 eq) was dissolved in THF:H_2O 1:1 (2 mL). To the solution was added 6-azidohezanoic acid (35.0 mg, 0.22 mmol, 3.70 eq), copper(II)sulfate (5.0 mg, 31 µmol, 0.52 eq) and sodium L-ascorbate (6.0 mg, 30 µmol, 0.50 eq). The turbid green solution was stirred for 3 h at 25°C, after which completion was observed by LC-MS. The reaction was diluted with EtOAc, then washed with 0.1 M aq. HCl (1x) and brine (1x). The aqueous layer was extracted with EtOAc at each step (1x). The combined organic layers were dried over $MgSO_4$ and concentrated under reduced pressure to yield a yellow oil (102 mg). Purification by flash chromatography (C18, 0-100% MeOH/H_2O, 0.1% TFA), afforded triazole **s16** as a transparent wax (TFA salt, 50 mg, 49 µmol, 82% yield). *Note: Crude product unstable in MeCN.*

^{1}H NMR (600 MHz, $CDCl_3$) δ 11.77 (br s, 1OH), 8.33 (d, *J* = 1.8 Hz, 1H), 8.30 (d, *J* = 8.6 Hz, 1H), 8.03 (d, *J* = 8.3 Hz, 2H), 7.73 (d, *J* = 8.7 Hz, 1H), 7.68 (dd, *J* = 8.7, 1.8 Hz, 1H), 7.55 (s, 1H), 7.52 (d, *J* = 8.5 Hz, 1H), 7.26 (d, *J* = 8.3 Hz, 2H), 7.21 (d, *J* = 7.5 Hz, 1NH), 6.91 (d, *J* = 8.0 Hz, 1 NH), 6.08 (dd, *J* = 8.5, 5.0 Hz, 1H), 5.81 (ddt, *J* = 17.2, 10.2, 7.1 Hz, 1H), 5.22 (t, *J* = 7.1 Hz, 1H), 5.16 (d, *J* = 10.3 Hz, 1H), 5.15 – 5.09 (m, 1H), 4.77 (q, *J* = 7.1 Hz, 1H), 4.37 (t, *J* = 6.8 Hz, 2H), 4.20 (br s, 1H), 3.81 – 3.70 (m, 1 NH), 3.70 – 3.55 (m, 1H), 3.09 (dd, *J* = 13.6, 6.5 Hz, 1H), 3.04 (dd, *J* = 13.6, 6.7 Hz, 1H), 2.97 (d, *J* = 7.0 Hz, 2H), 2.85 (t, *J* = 7.3 Hz, 2H), 2.32 (t, *J* = 7.0 Hz, 2H), 2.16 – 2.02 (m, 3H), 1.92 (p, *J* = 7.3 Hz, 2H), 1.90 – 1.85 (m, 2H), 1.85 – 1.75 (m, 2H), 1.75 – 1.65 (m, 1H), 1.64 (p, *J* = 8.1, 7.0 Hz, 2H), 1.37 – 1.27 (m, 2H), 1.24 (d, *J* = 6.9 Hz, 3H).

^{13}C NMR (151 MHz, $CDCl_3$) δ 176.25, 173.18, 170.96, 170.22, 169.50, 160.29 (q, TFA) 159.56, 146.85, 146.18, 145.95, 144.05, 139.38, 131.14, 130.62, 130.32, 129.55, 129.06, 126.32, 125.55, 123.46, 122.25, 119.71, 118.82, 115.42 (q, TFA), 76.41, 58.86, 53.69, 50.45, 46.07, 41.75, 41.06, 38.47, 34.01, 33.43, 29.38, 28.17, 25.40, 25.03, 24.25, 23.74, 22.56, 18.53.

HRMS (ESI+): calcd. for $[C_{42}H_{50}N_9O_9+H]^+$ (MH^+) 904.2988; found 904.2996.

LC trace (254 nM)

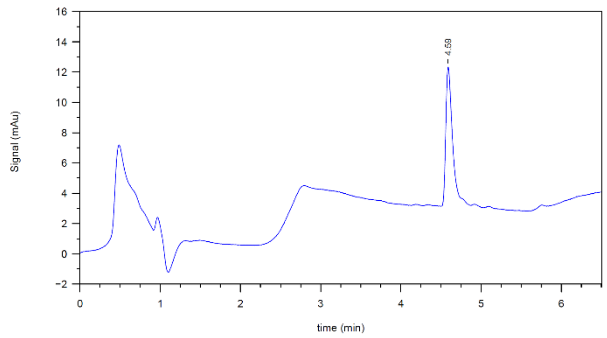

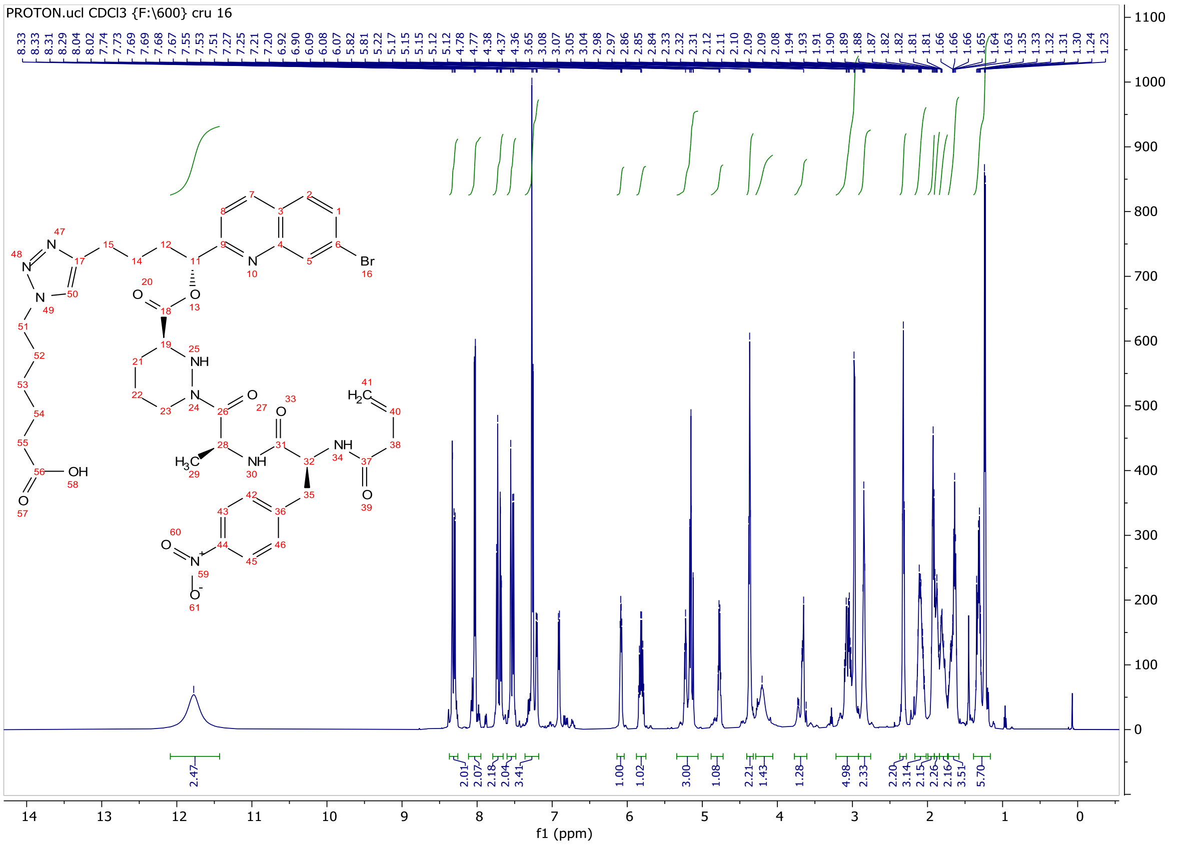
PROTON.ucl CDCl3 {F:\600} cru 16
f1 (ppm)
Br
N
NH
OH

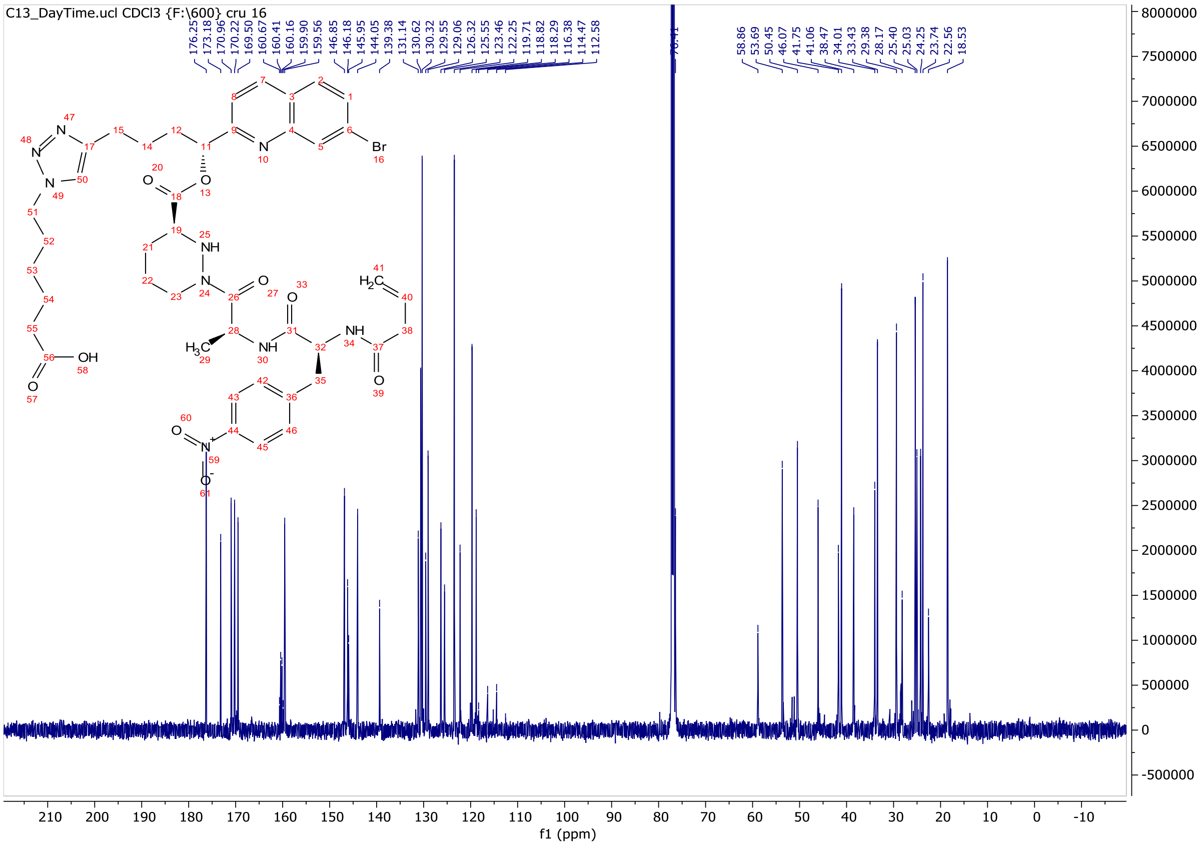
C13_DayTime.ucl CDCl3 {F:\600} cru 16
f1 (ppm)
Br
N
NH
OH

6-(4-((S)-3-((S)-3-(((R)-1-(7-bromoquinolin-2-yl)ethoxy)carbonyl)tetrahydropyridazin-1(2H)-yl)-2-((S)-2-(but-3-enamido)-3-(4-nitrophenyl)propanamido)-3-oxopropyl)-1H-1,2,3-triazol-1-yl)hexanoic acid (s17)

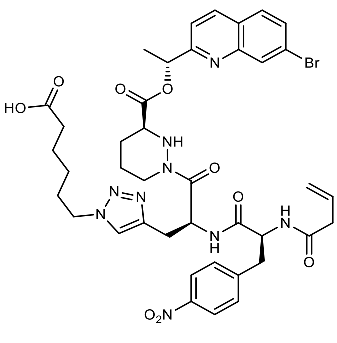

s17

Terminal alkyne **7c** (21.0 mg, 29 µmol, 1.00 eq) was dissolved in 1:1 THF:H_2O (1 mL). To the solution were added 6-azidohexanoic acid (9.2 mg, 58 µmol, 2.00 eq), Copper(II)sulfate (0.9 mg, 5.6 µmol, 19 mol-%) and sodium L-ascorbate (1.2 mg, 6.1 µmol, 21 mol-%). The reaction was stirred for 15 h at 25°C, after which completion was observed by LC-MS. The reaction was diluted with EtOAc and washed with H_2O (1x). The organic layer was dried over $MgSO_4$ and concentrated under reduced pressure. Purification by flash chromatography (C18, 25-100% MeOH/H_2O, 0.1% TFA) afforded triazole **s17** as a white solid (TFA salt, 25 mg, 29 µmol, 91%). *Note: Crude product unstable in MeCN.*

^{1}H NMR (600 MHz, Acetone-d6) δ 8.39 (d, *J* = 8.5 Hz, 1H), 8.19 (d, *J* = 2.2 Hz, 1H), 8.07 (d, *J* = 8.6 Hz, 2H), 7.89 (d, *J* = 8.7 Hz, 1H), 7.79 (s, 1H), 7.73 – 7.67 (m, 2H), 7.47 (dt, *J* = 16.5, 8.0 Hz, 2H&2NH), 6.07 (q, *J* = 6.6 Hz, 1H), 5.85 (ddtd, *J* = 17.1, 10.1, 6.7, 2.9 Hz, 1H), 5.54 (s, 1H), 5.06 (dq, *J* = 17.2, 1.8 Hz, 1H), 5.02 (dt, *J* = 10.1, 1.6 Hz, 1H) , 4.96 – 4.82 (m, 1 NH), 4.76 (td, *J* = 8.4, 5.4 Hz, 1H), 4.32 (td, *J* = 6.9, 3.3 Hz, 2H), 4.09 (very br s, COOH+H2O), 4.07 – 4.01 (m, 2H), 3.92 (br s, 1H), 3.17 (d, *J* = 5.1 Hz, 2H), 3.15 – 3.12 (m, 1H), 3.01 – 2.98 (m, 1H), 2.96 (d, *J* = 6.9 Hz, 2H), 2.28 (t, *J* = 7.1 Hz, 2H), 1.93 – 1.69 (m, 6H), 1.67 (d, *J* = 6.7 Hz, 3H), 1.64 – 1.55 (m, 2H), 1.35 – 1.23 (m, 2H).

^{13}C NMR (151 MHz, Acetone) δ 174.89, 171.49, 171.46, 171.21, 170.55, 162.74, 148.78, 147.63, 146.76, 143.21, 138.29, 133.08, 131.99, 131.46, 130.63, 130.51, 127.16, 124.05, 123.95, 123.81, 119.62, 118.14, 74.67, 59.25 (br), 54.89, 50.71, 50.28, 42.08, 41.46, 38.19, 33.90, 28.85, 28.54 (br), 27.48, 26.31, 24.83, 23.17 (br), 20.73.

LCMS calcd. for $[C_{40}H_{46}BrN_9O_9+H]^+$ (MH^+) 876.26; found 876.2.

LC trace (254 nM)

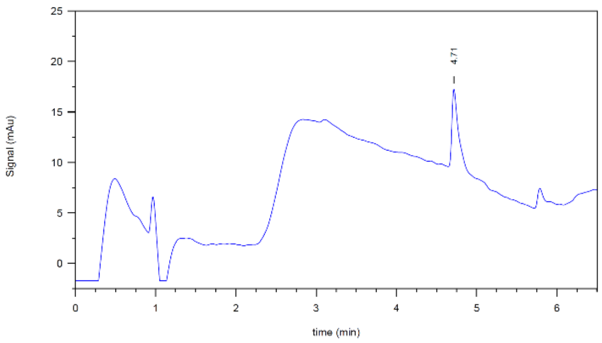

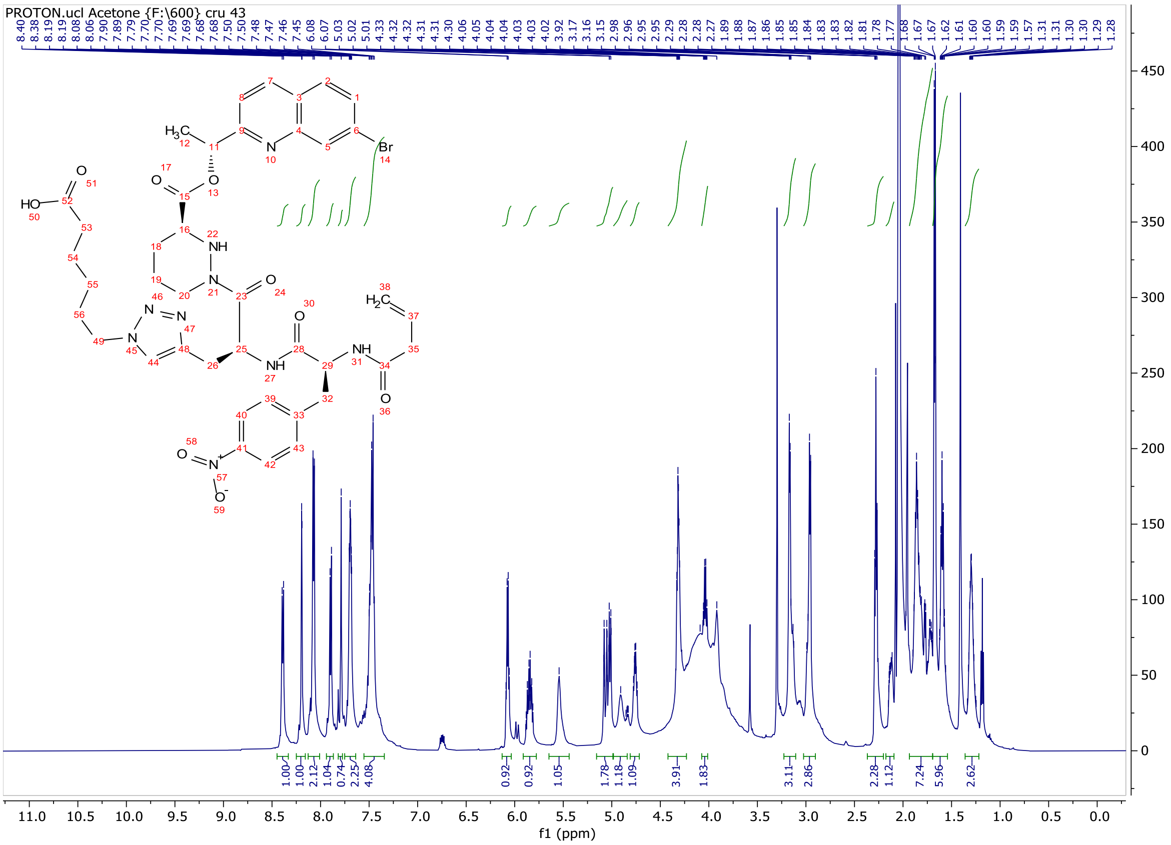
PROTON.ucl Acetone {F:\600} cru 43
f1 (ppm)

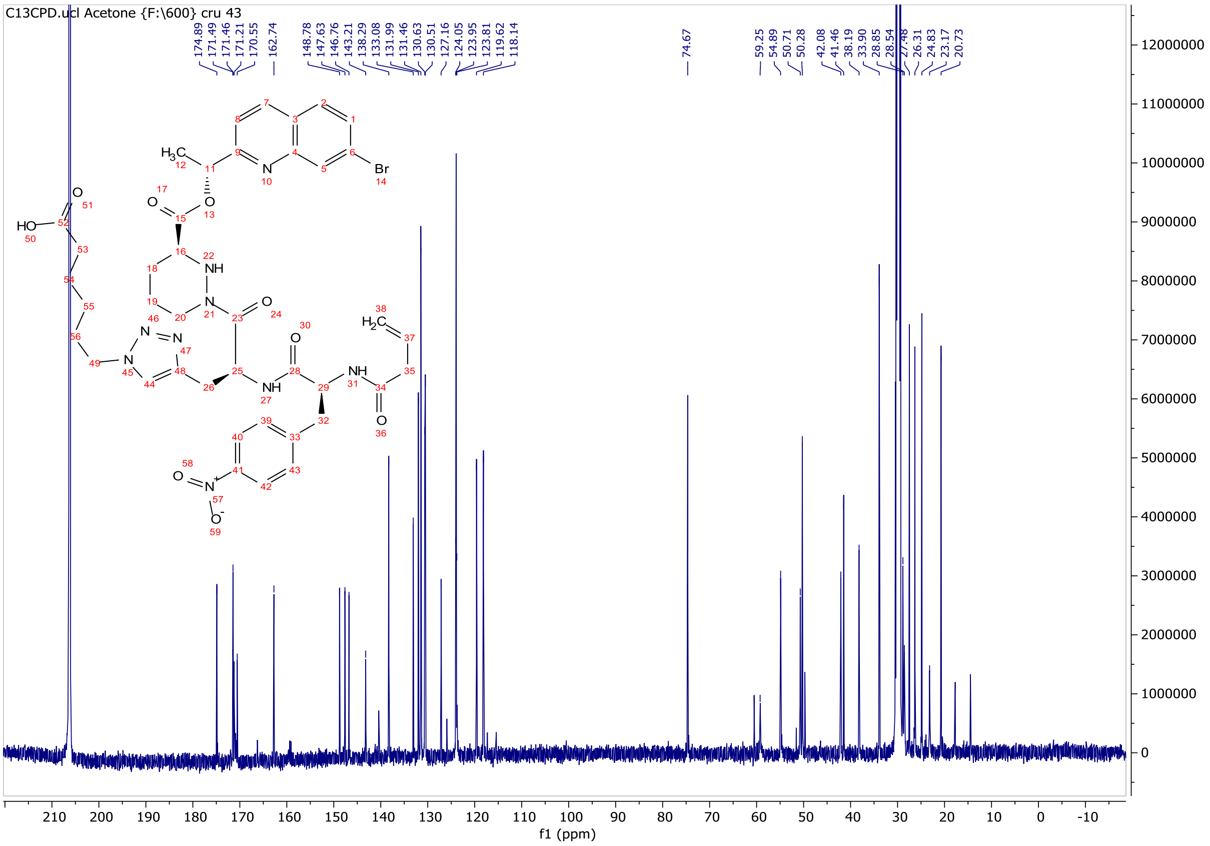
C13CPD.ucl Acetone {F:\600} cru 43
174.89
171.49
171.46
171.21
170.55
162.74
148.78
147.63
146.76
143.21
138.29
133.08
131.99
131.46
130.63
130.51
127.16
124.05
123.95
123.81
119.62
118.14
74.67
59.25
54.89
50.71
50.28
42.08
41.46
38.19
33.90
28.85
28.54
27.48
26.31
24.83
23.17
20.73
f1 (ppm)

(5S,2R,7S,10S,E)-2,7-dimethyl-10-(4-nitrobenzyl)-hexahydro-3-oxa-8,11-diaza-1(2,7)-quinolina-5(3,1)-pyridazinacyclopentadecaphan-14-ene-4,6,9,12-tetraone (TWH106)

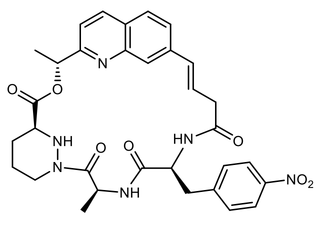

TWH106

In a microwave vial, intermediate 7**a** (220 mg, 0.32 mmol, 1.00 eq), piperazine (68 mg, 0.79 mmol, 2.50 eq) and Palladium(II) acetate (14 mg, 62 µmol, 20 mol-%) were combined in dry DMF (20 mL). The reaction vessel was flushed with N_2 and the reaction heated to 85°C under microwave irradiation for 3 h, after which consumption of the starting material was visible into several products by LC-MS. The reaction was concentrated under reduced pressure, dissolved in MeOH, and filtered over a thiol scavenger column equilibriated with MeOH (Isolute Si-Thiol, Biotage). The residue was purified by flash chromatography (C18, 30-60% $MeCN/H_2O$, 0.1% TFA then silica, 3% $MeOH/CH_2Cl_2$). To separate out isomers, the obtained white solid was further purified by preparative TLC (3% $MeOH/CH_2Cl_2$) to yield **TWH106** as a white solid (15 mg, 24 µmol, 8% yield, *trans* double bond product).

R_f = 0.25 (3% $MeOH/CH_2Cl_2$).

^{1}H NMR (600 MHz, DMSO-d6) δ 8.55 (d, *J* = 9.5 Hz, 1NH), 8.53 (d, *J* = 7.5 Hz, 1NH), 8.29 (d, *J* = 8.5 Hz, 1H), 8.17 (d, *J* = 8.7 Hz, 2H), 7.88 (d, *J* = 8.6 Hz, 1H), 7.81 (dd, *J* = 8.6, 1.7 Hz, 1H), 7.59 (d, *J* = 8.6 Hz, 2H), 7.57 (d, *J* = 1.6 Hz, 1H), 7.49 (d, *J* = 8.5 Hz, 1H), 6.53 (dt, *J* = 16.4, 4.9 Hz, 1H), 6.30 (dt, *J* = 16.6, 2.3 Hz, 1H), 5.97 (q, *J* = 6.9 Hz, 1H), 5.70 (p, *J* = 7.2 Hz, 1H), 4.98 (d, *J* = 12.3 Hz, 1NH), 4.85 (td, *J* = 9.9, 9.5, 5.1 Hz, 1H), 4.25 (d, *J* = 12.7 Hz, 1H), 3.71 (td, *J* = 12.0, 11.0, 2.9 Hz, 1H), 3.12 (ddd, *J* = 15.4, 5.0, 2.0 Hz, 1H), 2.96 (dd, *J* = 14.0, 5.1 Hz, 1H), 2.92 (dd, *J* = 14.1, 9.5 Hz, 1H), 2.78 (dd, *J* = 12.7, 3.2 Hz, 1H), 2.74 (ddd, *J* = 15.8, 4.9, 2.1 Hz, 1H), 1.99 – 1.91 (m, 1H), 1.87 – 1.75 (m, 1H), 1.65 (d, *J* = 6.9 Hz, 3H), 1.63 – 1.55 (m, 2H), 1.50 (d, *J* = 7.1 Hz, 3H).

^{13}C NMR (151 MHz, DMSO-d6) δ 172.76, 171.14, 170.54, 170.49, 159.02, 147.01, 146.57, 146.24, 137.97, 136.22, 130.49, 130.11, 128.02, 127.86, 126.31, 126.17, 123.27, 122.41, 118.29, 72.05, 59.97, 53.18, 44.58, (40.15, 40.05, under DMSO peak), 36.06, 27.35, 22.67, 19.98, 19.81.

HRMS (ASAP HESI+): calcd. for $[C_{32}H_{34}N_6O_7+H]^+$ (MH^+) 615.2562; found 615.2561.

LC trace (254 nM)

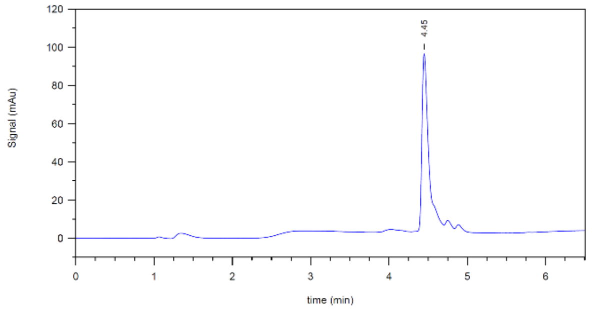

TWH106 assignments

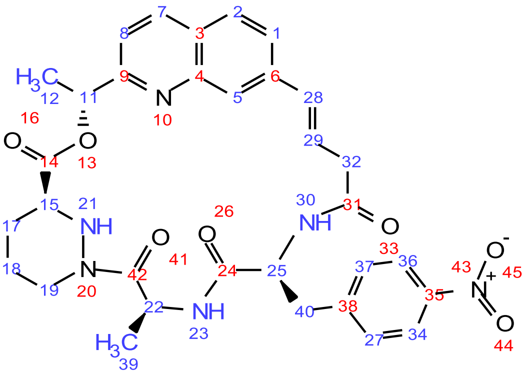

Atom	δ (ppm)	J (Hz)	Atom	δ (ppm)	J (Hz)
1 C	122.41		23 N		
H	7.81	8.60(2), 1.70(5)	H	8.53	7.50(22)
2 C	127.86		24 C	170.49	
H	7.88	8.60(1)	25 C	53.18	
3 C	126.17		H	4.85	9.50(30), 9.50(40'), 5.10(40")
4 C	147.01		27 C	130.49	
5 C	128.02		H	7.59	8.70(34), 8.70(36)
H	7.57	1.70(1)	28 C	130.1	
6 C	137.97		H	6.3	16.40(29), 2.20(32'), 2.20(32")
7 C	136.22		29 C	126.31	
H	8.29	8.50(8)	H	6.53	4.90(32'), 4.90(32"), 16.40(28)
8 C	118.29		30 N		
H	7.49	8.50(7)	H	8.55	9.50(25)
9 C	159.02				
11 C	72.05		31 C	170.54	
H	5.97	6.90(12)	32 C	40.15	
12 C	19.81 / 19.98		H'	3.12	2.20(28), 4.90(29), 15.40(32")
H3	1.65	6.90(11)	H"	2.74	4.90(29), 2.20(28), 15.40(32')
14 C	171.14		34 C	123.27	
15 C	59.97		H	8.17	8.70(27), 8.70(37)
H	3.71	2.90(17'), 11.00(17"), 12.10(21)	35 C	146.24	
17 C	27.35		36 C	123.27	
H'	1.6	2.90(15)	H	8.17	8.70(27), 8.70(37)
H"	1.96	11.00(15)	37 C	130.49	
18 C	22.67		H	7.59	8.70(34), 8.70(36)
H'	1.6		38 C	146.57	
H"	1.8	3.20(19')	39 C	19.81 / 19.98	
19 C	40.05		H3	1.50	7.20(22)
H'	4.25	3.20(18"), 12.50(19")	40 C	36.06	
H"	2.78	12.50(19')	H'	2.96	9.50(25), 14.10(40")
21 N			H"	2.92	5.10(25), 14.10(40')
H	4.98	12.10(15)	42 C	172.76	
22 C	44.58				
H	5.7	7.50(23), 7.20(39)			

Table S1 – Assignments of TWH106 in DMSO-d_6. Numbering scheme of the molecule is shown above the table. Assignments were made based on COSY, HSQC, HMBC and NOESY spectra. Coupling constant values *J* are given with their coupling partner shown in parentheses.

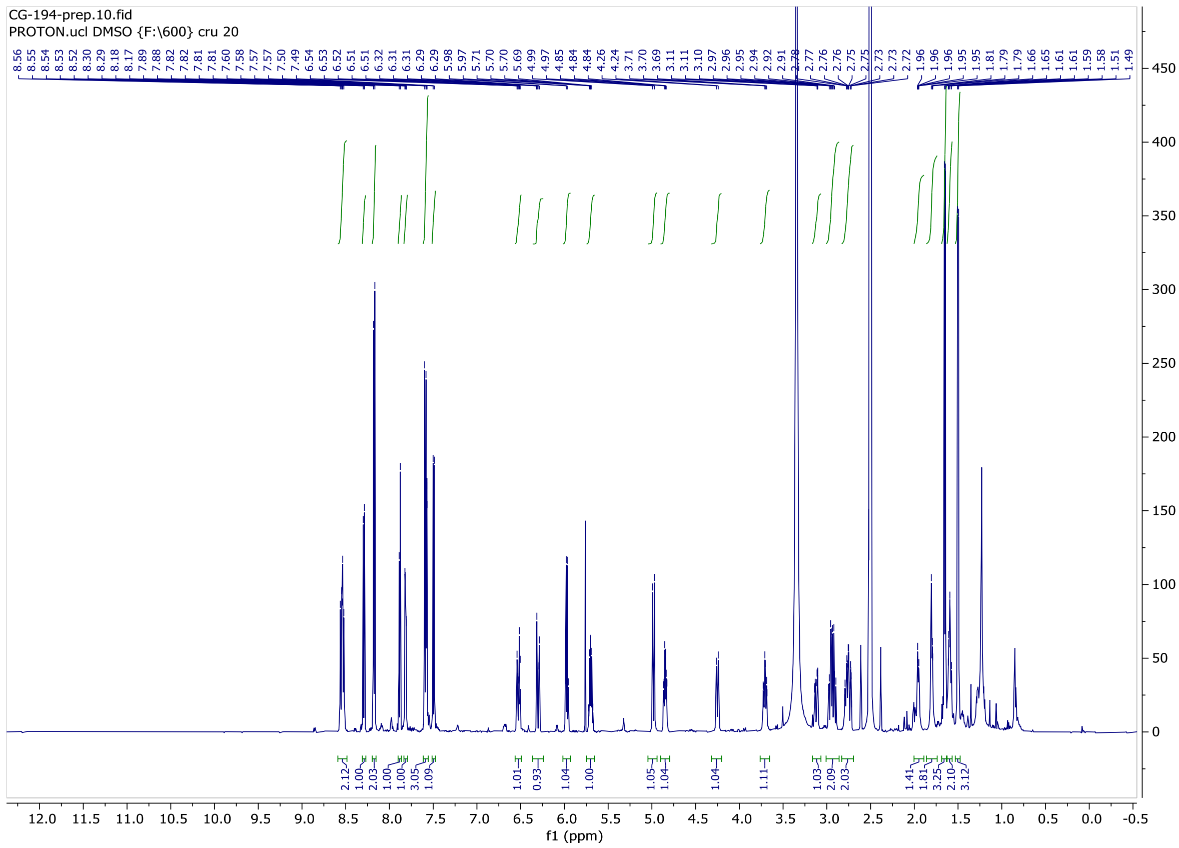

CG-194-prep.10.fid
PROTON.ucl DMSO {F:\600} cru 20
f1 (ppm)

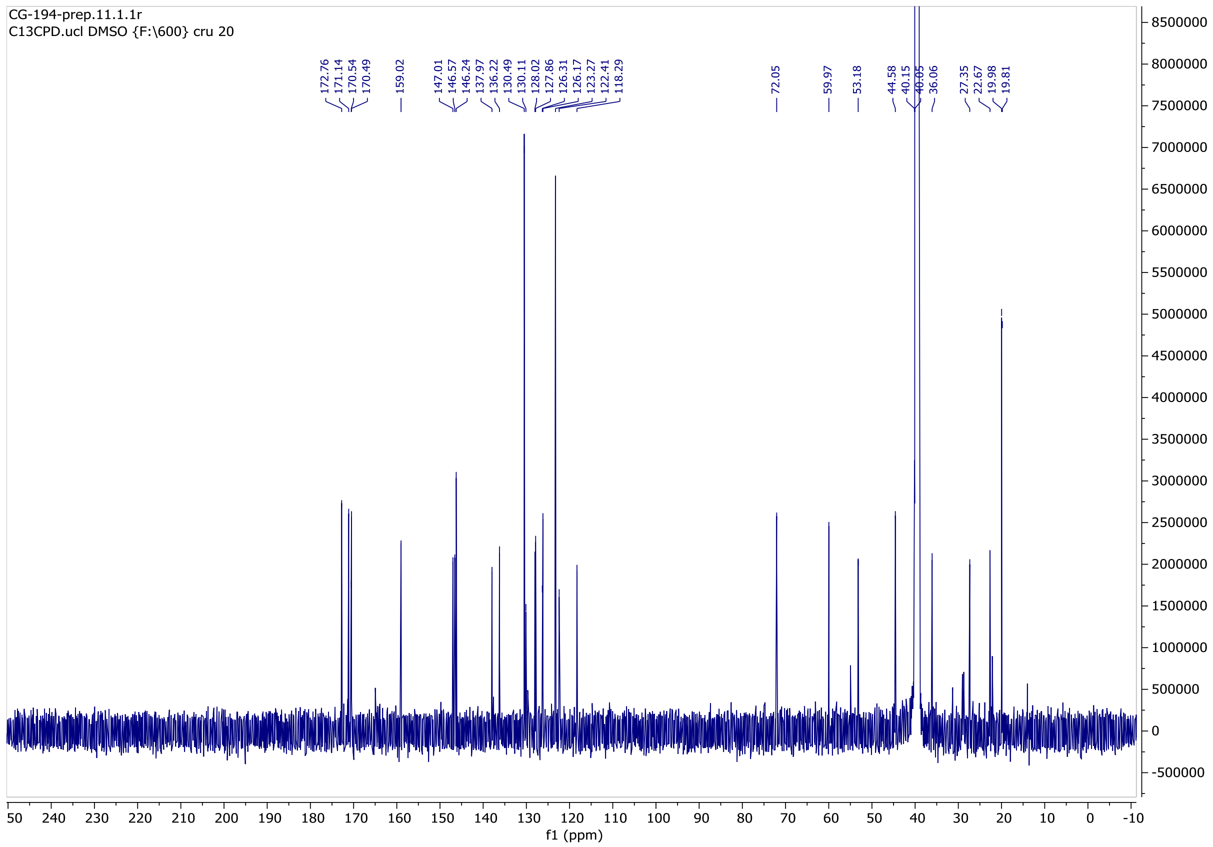

CG-194-prep.11.1.1r
C13CPD.ucl DMSO {F:\600} cru 20
172.76
171.14
170.54
170.49
159.02
147.01
146.57
146.24
137.97
136.22
130.49
130.11
128.02
127.86
126.31
126.17
123.27
122.41
118.29
72.05
59.97
53.18
44.58
40.15
40.05
36.06
27.35
22.67
19.98
19.81
f1 (ppm)

6-(4-(3-((5S,2R,7S,10S,E)-7-methyl-10-(4-nitrobenzyl)-4,6,9,12-tetraoxo-51,52,53,54,55,56-hexahydro-3-oxa-8,11-diaza-1(2,7)-quinolina-5(3,1)-pyridazinacyclopentadecaphan-14-en-2-yl)propyl)-1H-1,2,3-triazol-1-yl)hexanoic acid (s18)

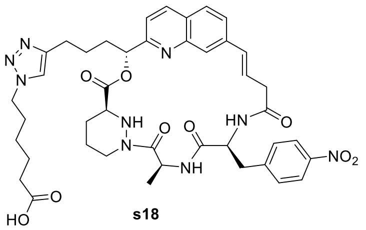

s18

In a microwave vial, intermediate **s16** (115 mg, 0.13 mmol, 1.00 eq) was dissolved in dry DMF (9 mL). To the solution was added Palladium(II)acetate (4.5 mg, 0.02 mmol, 16 mol-%) and piperazine (23 mg, 0.27 mmol, 2.10 eq). The reaction was flushed with N_2, then stirred for 1.5 h at 85°C under microwave irradiation, showing completion by LC-MS into several products. The solvents were removed under reduced pressure and the residue was dissolved in MeOH. The solution was filtered over a thiol scavenger column equilibriate with MeOH (Isolute Si-Thiol, Biotage) and concentrated under reduced pressure to yield a brown oil (220 mg). The oil was purified by flash chromatography (C18, 0-100% MeCN/H2O, 0.1% FA then silica 0-5% MeOH/CH_2Cl_2) to afford the cyclised product **s18** as a white solid (7.0 mg, 8.5 µmol, 7% yield, *trans* double bond product).

R_f = 0.18 in 5% MeOH/CH_2Cl_2.

^{1}H NMR (700 MHz, CD_3OD) δ 8.15 (d, *J* = 8.8 Hz, 1H), 8.13 (d, *J* = 8.7 Hz, 2H), 7.74 (d, *J* = 8.5 Hz, 1H), 7.73 (s, 1H), 7.69 (dd, *J* = 8.5, 1.7 Hz, 1H), 7.62 (d, *J* = 1.6 Hz, 1H), 7.48 (d, *J* = 8.7 Hz, 2H), 7.36 (d, *J* = 8.4 Hz, 1H), 6.49 (ddd, *J* = 16.4, 5.9, 4.0 Hz, 1H), 6.41 (dt, *J* = 16.3, 1.9 Hz, 1H), 5.82 (dd, *J* = 8.8, 4.1 Hz, 1H), 5.62 (q, *J* = 7.2 Hz, 1H), 4.90 (dd, *J* = 9.4, 6.1 Hz, 1H), 4.46 – 4.38 (m, 1H), 4.33 (t, *J* = 7.0 Hz, 2H), 3.80 (dd, *J* = 11.4, 2.9 Hz, 1H), 3.22 (ddd, *J* = 15.6, 5.9, 1.6 Hz, 1H), 3.20 (dd, *J* = 14.5, 6.2 Hz, 1H), 2.99 (dd, *J* = 14.2, 9.4 Hz, 1H), 2.93 (ddd, *J* = 15.7, 4.0, 2.1 Hz, 1H), 2.81 (dd, *J* = 15.2, 7.4 Hz, 1H), 2.77 (dd, *J* = 15.0, 7.6 Hz, 1H), 2.79 – 2.71 (m, 1H), 2.24 (t, *J* = 7.4 Hz, 2H), 2.19 – 2.11 (m, 1H), 2.13 – 2.05 (m, 1H), 2.05 – 1.98 (m, 1H), 1.89 (ddt, *J* = 18.0, 14.7, 7.4 Hz, 5H), 1.75 – 1.65 (m, 2H), 1.64 – 1.57 (m, 2H), 1.57 (d, *J* = 7.2 Hz, 3H), 1.36 – 1.25 (m, 2H).

^{13}C NMR (176 MHz, CD_3OD) δ 177.95, 175.63, 173.91, 172.74, 159.82, 148.85, 148.50, 148.19, 146.74, 139.49, 137.40, 132.03, 131.28, 129.07, 128.87, 128.01, 126.53, 124.36, 123.80, 123.19, 119.72, 77.84, 61.42, 54.70, 50.95, 47.11, 42.42, 40.19, 36.88, 34.84, 34.72, 30.85, 28.57, 26.91, 26.51, 25.85, 25.36, 23.73, 18.54.

HRMS (ESI+): calcd. for $[C_{42}H_{49}N_9O_9+H]^+$ (MH^+) 824.3726; found 824.3724.

LC trace (254 nM)

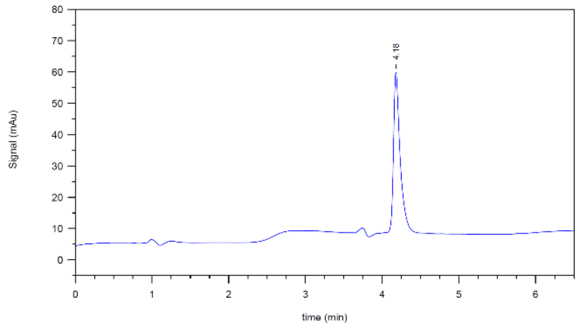

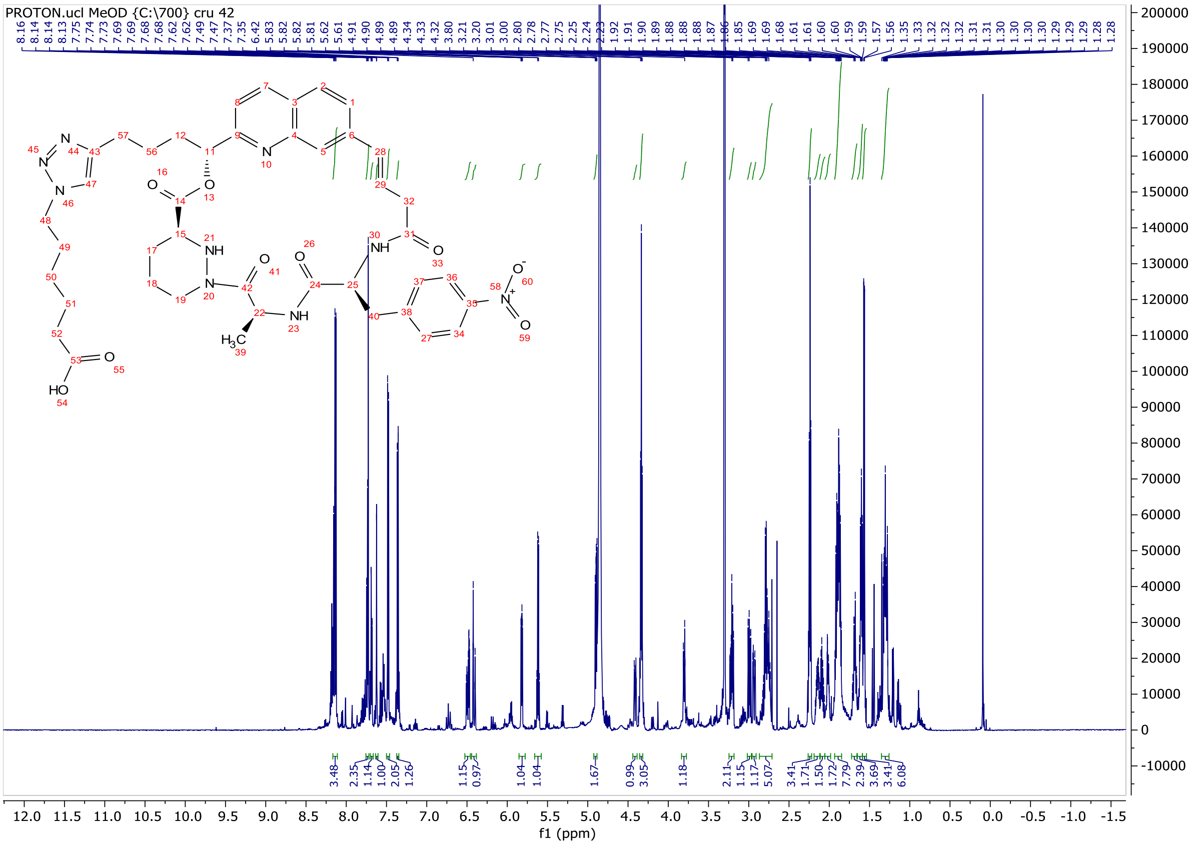

Assignments of carbons made based on HSQC and HMBC experiments due to low S/N (see raw data)

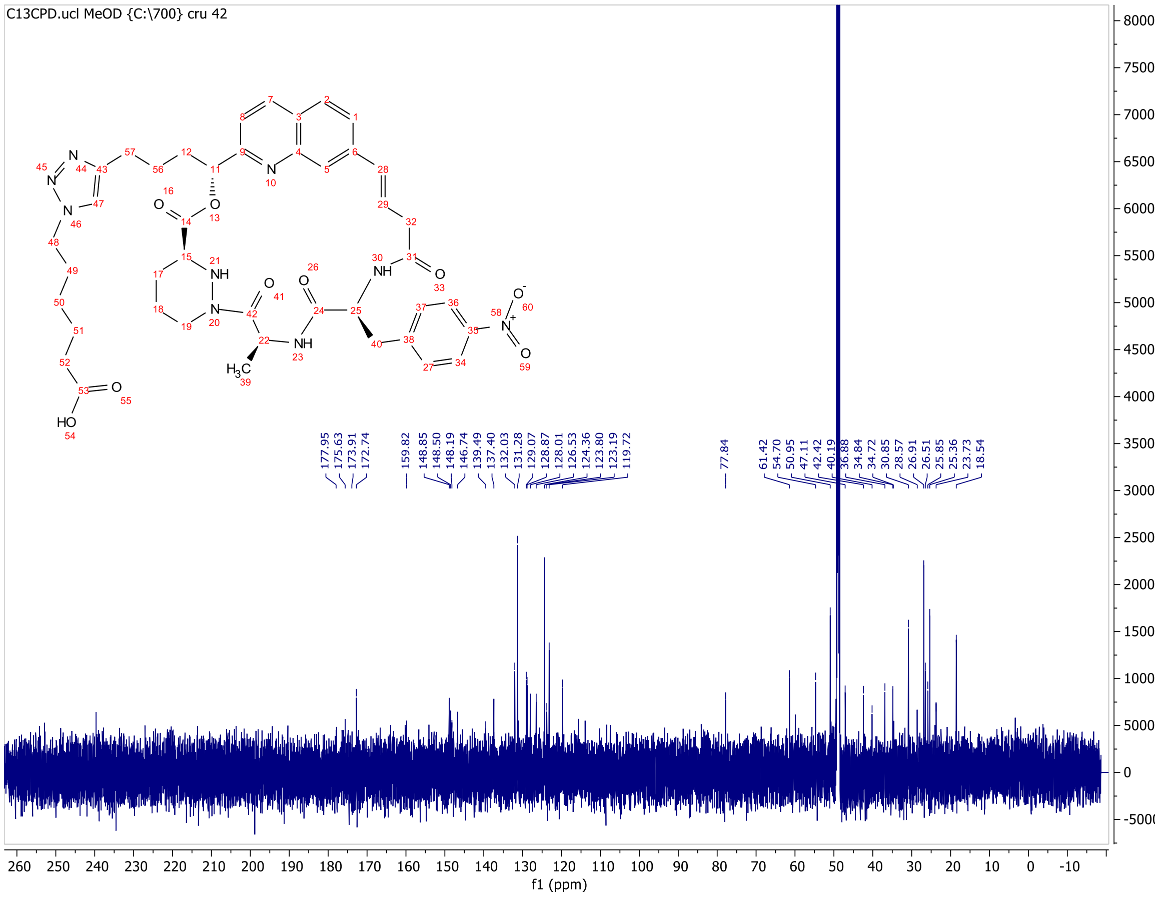

6-(4-(((5S,2R,7S,10S,E)-2-methyl-10-(4-nitrobenzyl)-4,6,9,12-tetraoxo-51,52,53,54,55,56-hexahydro-3-oxa-8,11-diaza-1(2,7)-quinolina-5(3,1)-pyridazinacyclopentadecaphan-14-en-7-yl)methyl)-1H-1,2,3-triazol-1-yl)hexanoic acid (s19)

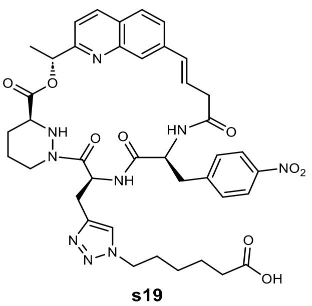

s19

In a microwave vial, intermediate **s17** (106 mg, 0.12 mmol, 1.00 eq), palladium(II)acetate (2.7 mg, 12 µmol, 10 mol-%), piperazine (20.8 mg, 0.24 mmol, 2.00 eq) were dissolved in dry DMF (3 mL). The vial was flushed with N_2 and heated to 85°C under microwave irradiation for 0.5 h, showing completion by LC-MS into several products. The solvents were removed under reduced pressure and the residue dissolved in MeOH. The solution was filtered through a thiol scavenger column (Isolute Si-Thiol, Biotage). The filtrate was concentrated under reduced pressure and the residue purified by flash chromatography (C18, 20-100% MeOH/H_2O, 0.1% FA, twice) to provide cyclised compound **s19** as a white solid (26.4 mg, 33 µmol, 27%, *trans* double bond product).

^{1}H NMR (600 MHz, CD_3OD) δ 8.98 (d, *J* = 8.5 Hz, 1H), 8.17 (d, *J* = 8.7 Hz, 1H), 8.15 (d, *J* = 8.7 Hz, 2H), 8.08 (d, *J* = 1.7 Hz, 1H), 8.01 (d, *J* = 8.5 Hz, 1H), 7.91 (dd, *J* = 8.6, 1.5 Hz, 1H), 7.51 (s, 1H), 7.50 (d, *J* = 8.7 Hz, 2H), 6.43 (dd, *J* = 16.2, 1.9 Hz, 1H), 6.30 (q, *J* = 6.8 Hz, 1H), 6.23 (ddd, *J* = 16.1, 8.0, 4.0 Hz, 1H), 5.96 (dd, *J* = 10.7, 3.4 Hz, 1H), 4.81 (dd, *J* = 9.9, 5.3 Hz, 1H), 4.41 (ddd, *J* = 13.8, 2.9, 1.9 Hz, 1H), 4.37 (dd, *J* = 13.7, 6.9 Hz, 1H), 4.33 (dd, *J* = 13.6, 7.0 Hz, 1H), 3.73 (dd, *J* = 11.4, 2.8 Hz, 1H), 3.45 – 3.36 (m, 1H), 3.24 (ddd, *J* = 14.3, 8.0, 1.3 Hz, 1H), 3.19 (dd, *J* = 14.1, 5.3 Hz, 1H), 3.15 – 3.06 (m, 1H), 2.94 (dd, *J* = 14.1, 9.9 Hz, 1H), 2.92 (ddd, *J* = 14.4, 4.0, 2.2 Hz, 1H), 2.71 (td, *J* = 13.0, 3.2 Hz, 1H), 2.19 (t, *J* = 7.4 Hz, 2H), 2.00 (dq, *J* = 12.8, 3.2 Hz, 1H), 1.93 – 1.87 (m, 1H), 1.87 – 1.78 (m, 2H), 1.83 (d, *J* = 6.9 Hz, 3H), 1.76 – 1.68 (m, 1H), 1.70 – 1.61 (m, 1H), 1.53 (ddt, *J* = 14.5, 9.0, 7.1 Hz, 2H), 1.26 (dq, *J* = 15.4, 8.2, 7.6 Hz, 2H).

^{13}C NMR (151 MHz, CD_3OD) δ 177.17, 173.70, 173.52, 173.25, 170.86, 159.61, 148.32, 146.71, 146.52, 144.89, 141.97, 132.46, 131.73, 131.41, 130.21, 129.06, 128.20, 125.98, 124.52, 124.47, 121.28, 120.97, 72.36, 61.16, 54.92, 52.00, 51.52, 42.64, 41.41, 37.98, 34.47, 30.89, 28.47, 27.28, 26.93, 25.26, 23.71, 20.61.

HRMS (ESI+): calcd. for $[C_{40}H_{45}N_9O_9+H]^+$ (MH^+) 796.3413; found 796.3412.

LC trace (254 nM)

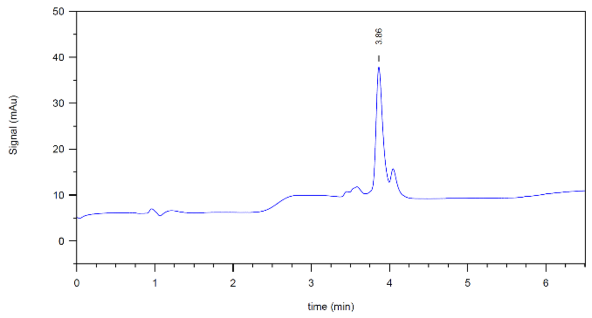

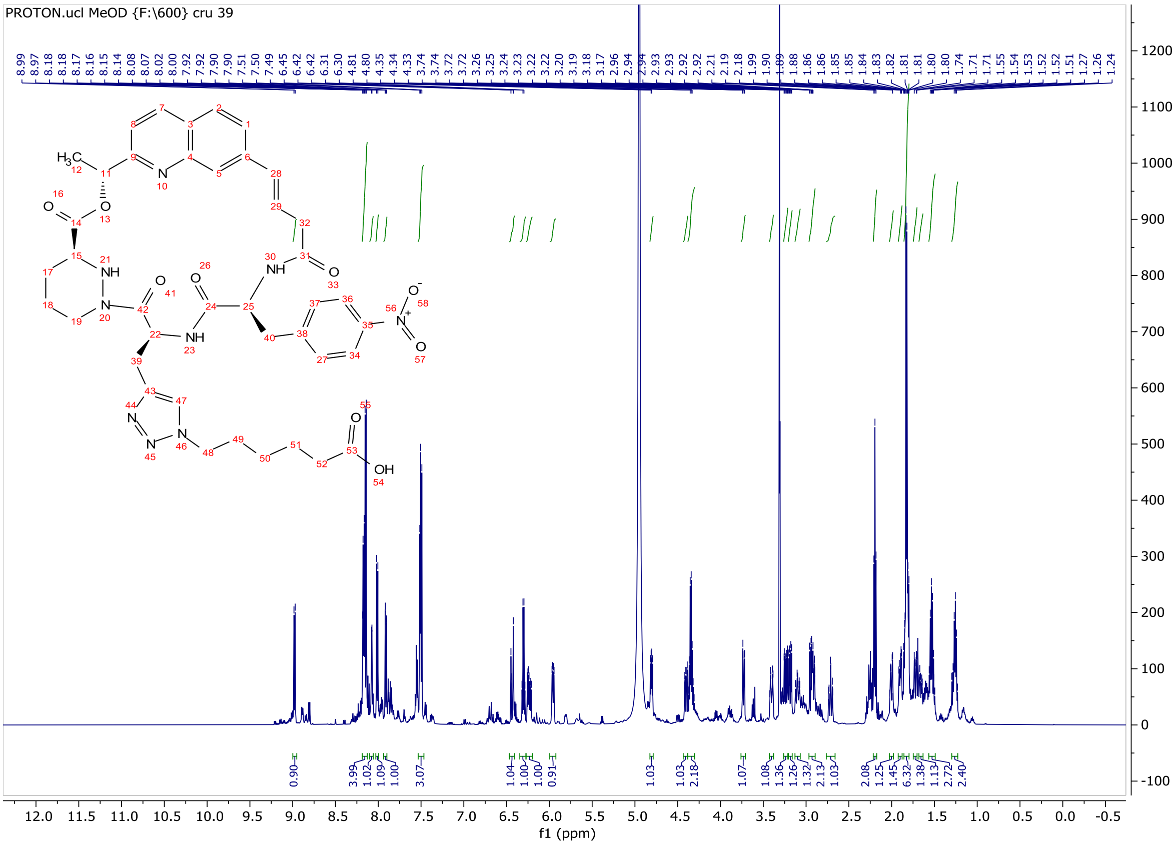
PROTON.ucl MeOD {F:\600} cru 39
8.99 8.97 8.18 8.17 8.16 8.15 8.14 8.08 8.07 8.02 8.00 7.92 7.92 7.90 7.90 7.51 7.50 7.49 6.45 6.42 6.42 6.31 6.30 4.81 4.80 4.35 4.34 4.33 3.74 3.74 3.72 3.72 3.26 3.25 3.24 3.23 3.22 3.22 3.20 3.19 3.18 3.17 2.96 2.94 2.94 2.93 2.93 2.92 2.92 2.21 2.19 2.18 1.99 1.90 1.89 1.88 1.86 1.86 1.85 1.85 1.84 1.83 1.82 1.81 1.81 1.80 1.80 1.74 1.71 1.71 1.55 1.54 1.53 1.52 1.52 1.51 1.27 1.26 1.24
0.90 3.99 1.02 1.09 1.00 3.07 1.04 1.00 1.00 0.91 1.03 1.03 2.18 1.07 1.08 1.36 1.26 1.32 2.13 1.03 2.08 1.25 1.45 6.32 1.38 1.13 2.72 2.40
f1 (ppm)

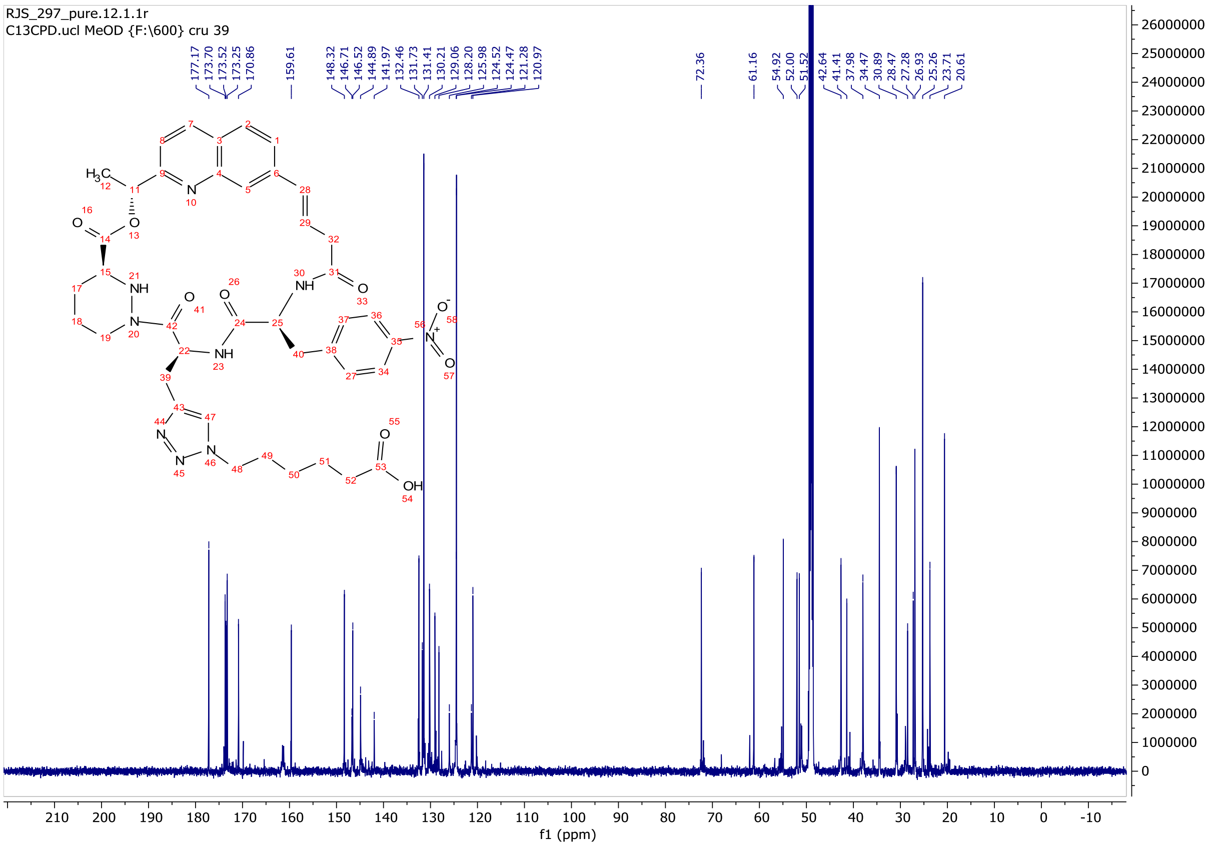
RJS_297_pure.12.1.1r
C13CPD.ucl MeOD {F:\600} cru 39
177.17 173.70 173.52 173.25 170.86 159.61 148.32 146.71 146.52 144.89 141.97 132.46 131.73 131.41 130.21 129.06 128.20 125.98 124.52 124.47 121.28 120.97 72.36 61.16 54.92 52.00 51.52 42.64 41.41 37.98 34.47 30.89 28.47 27.28 26.93 25.26 23.71 20.61
f1 (ppm)

(2S,4R)-1-((2S)-3,3-dimethyl-2-(6-(4-(3-((53S,2R,7S,10S,E)-7-methyl-10-(4-nitrobenzyl)-4,6,9,12-tetraoxo-51,52,53,54,55,56-hexahydro-3-oxa-8,11-diaza-1(2,7)-quinolina-5(3,1)-pyridazinacyclopentadecaphan-14-en-2-yl)propyl)-1H-1,2,3-triazol-1-yl)hexanamido)butanoyl)-4-hydroxy-N-((S)-1-(4-(4-methylthiazol-5-yl)phenyl)ethyl)pyrrolidine-2-carboxamide (CG167)

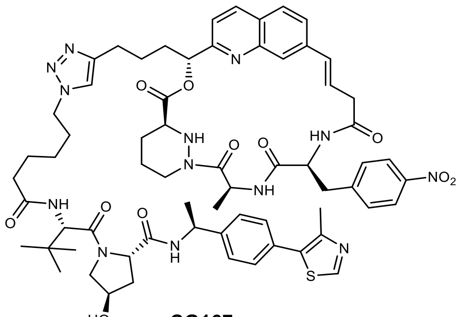

CG167

To a solution of carboxylic acid **s18** (5.5 mg, 6.7 µmol, 1.00 eq) in dry DMF (0.2 mL) was added HATU (2.8 mg, 7.3 µmol, 1.10 eq), *i*Pr$_2$NEt (3 µL, 15 µmol, 2.20 eq), and VHL ligand 2 ((S,R,S)-AHPC-Me) (4.1 mg, 7.9 µmol, 1.19 eq). The reaction was stirred for 3.5 h at 25°C after which completion was visible by LC-MS. The reaction was concentrated down under reduced pressure and the residue was directly purified by flash chromatography (C18, 20-100% MeCN/H2O, 0.1% TFA) to afford PROTAC **CG167** as a white solid (TFA salt, 7.5 mg, 5.3 µmol, 80% yield).

^{1}H NMR (600 MHz, DMSO-d_6) δ 8.99 (s, 1H), 8.53 (d, *J* = 9.4 Hz, 1NH), 8.51 (d, *J* = 8.5 Hz, 1NH), 8.40 (d, *J* = 7.8 Hz, 1NH), 8.28 (d, *J* = 8.5 Hz, 1H), 8.17 (d, *J* = 8.7 Hz, 2H), 7.88 (d, *J* = 8.6 Hz, 1H), 7.84 (s, 1H), 7.83 (dd, *J* = 8.8, 2.5 Hz, 1NH), 7.81 (dd, *J* = 8.7, 1.7 Hz, 1H), 7.59 (d, *J* = 8.7 Hz, 2H), 7.56 (s, 1H), 7.47 (d, *J* = 8.5 Hz, 1H), 7.43 (d, *J* = 8.3 Hz, 2H), 7.37 (d, *J* = 8.3 Hz, 2H), 6.52 (dt, *J* = 16.4, 4.8 Hz, 1H), 6.29 (dt, *J* = 16.4, 2.1 Hz, 1H), 5.88 (dd, *J* = 9.1, 3.7 Hz, 1H), 5.65 (p, *J* = 8.5, 7.1 Hz, 1H), 4.97 (d, *J* = 12.3 Hz, 1NH), 4.91 (dq, *J* = 7.8, 7.0 Hz, 1H), 4.84 (td, *J* = 9.9, 4.9 Hz, 1H), 4.50 (d, *J* = 9.4 Hz, 1H), 4.41 (t, *J* = 8.1 Hz, 1H), 4.30 – 4.21 (m, 4H&1OH), 3.71 (ddd, *J* = 11.8, 3.0 Hz, 1H), 3.65 – 3.55 (m, 2H), 3.12 (ddd, *J* = 15.7, 5.3, 2.1 Hz, 1H), 3.01 – 2.83 (m, 2H), 2.80 – 2.75 (m, 1H), 2.75 – 2.71 (m, 1H), 2.72 – 2.63 (m, 2H), 2.45 (s, 3H), 2.23 (dt, *J* = 14.5, 7.5 Hz, 1H), 2.17 – 2.11 (m, 1H), 2.11 – 2.06 (m, 1H), 2.04 – 1.98 (m, 1H), 2.01 – 1.94 (m, 1H), 1.95 – 1.91 (m, 1H), 1.88 – 1.71 (m, 6H), 1.60 (d, *J* = 5.8 Hz, 2H), 1.51 (d, *J* = 7.1 Hz, 3H), 1.56 – 1.42 (m, 2H), 1.36 (d, *J* = 7.0 Hz, 3H), 1.18 (m, 2H), 0.91 (s, 9H).

^{13}C NMR (151 MHz, DMSO-d_6) δ 172.75, 171.91, 171.34, 170.65, 170.57, 170.47, 169.59, 158.23, 158.19 (q, TFA), 151.59, 147.75, 147.12, 146.59, 146.35, 146.25, 144.73, 137.97, 136.11, 131.18, 130.50, 130.14, 129.69, 128.87, 127.99, 127.88, 126.41, 126.27, 126.21, 123.27, 122.48, 121.79, 118.53, 116.03 (q, TFA), 75.63, 68.79, 60.02, 58.57, 56.36, 56.32, 53.21, 49.09, 47.72, 44.66, 40.16, 39.13, 37.78, 36.05, 35.22, 34.65, 33.39, 29.50, 27.41, 26.46, 25.55, 25.36, 24.83, 24.76, 22.68, 22.51, 19.84, 16.01.

HRMS (ESI+): calcd. for $[C_{65}H_{79}N_{13}O_{11}+H]^+$ (MH+) 1250.5815; found 1250.5825.

LC-MS trace (254 nM)

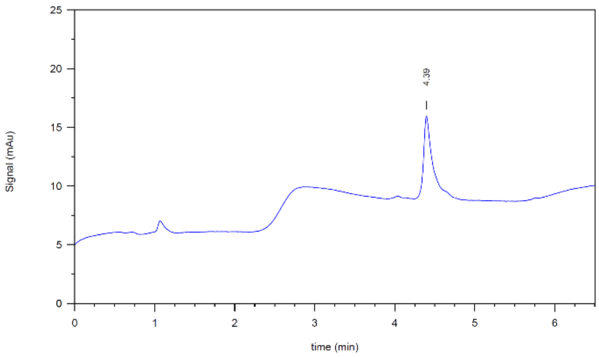

CG167 ASSIGNMENTS

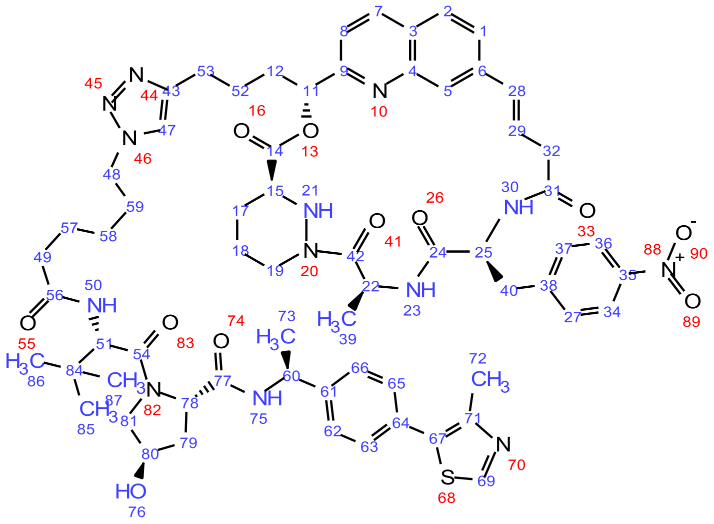

Atom	δ (ppm)	J (Hz)	Atom	δ (ppm)	J (Hz)
1 C	122.49		43 C	146.34	
H	7.81	8.70(2), 1.70(5)	47 C	121.79	
2 C	127.88		H	7.84	
H	7.88	8.70(1)	48 C	49.09	
3 C	126.21		H2	4.26	
4 C	147.12		49 C	34.65	
5 C	127.99		H'	2.23	14.50(49"), 7.50(57)
H	7.56	1.70(1)	H"	2.09	14.50(49')
6 C	137.97		50 N		
7 C	136.11		H	7.83	9.40(51)
H	8.28	8.50(8)	51 C	56.32	
8 C	118.52		H	4.5	9.40(50)
H	7.47	8.50(7)	52 C	25.36	
9 C	158.19		H2	1.77	
11 C	75.69		53 C	24.76	
H	5.88	9.10(12'), 3.70(12")	H2	2.67	
12 C	22.68		54 C	169.61	
H'	1.77	9.10(11)	56 C	171.9	
H"	1.60	3.70(11)	57 C	24.83	
14 C	171.34		H2	1.48	7.50(49')
15 C	60.04		58 C	25.57	
H	3.71	11.70(17'), 2.97(17"), 11.80(21)	H2	1.18	
17 C	33.39		59 C	29.53	
H'	2.12	11.70(15)	H2	1.77	
H"	1.98	2.97(15)	60 C	47.73	
18 C	27.41		H	4.91	7.00(73), 7.80(75)
H'	1.6		61 C	144.73	
H"	1.94		62 C	126.41	
19 C	40.17		H	7.37	8.30(63)
H'	2.77		63 C	128.87	
H"	4.26		H	7.43	8.30(62)
21 N			64 C	131.18	
H	4.97	11.80(15)	65 C	128.87	8.30(66)

22 C	44.66		H	7.43	
H	5.65	8.50(23), 7.20(39)	66 C	126.41	8.30(65)
23 N			H	7.37	
H	8.51	8.50(22)	67 C	129.69	
24 C	170.47		69 C	151.59	
25 C	53.18		H	8.99	
H	4.84	9.93(30), 4.90(40''), 9.80(40')	71 C	147.75	
27 C	130.5		72 C	16.07	
H	7.59	8.70(34)	H3	2.45	
28 C	130.12		73 C	22.51	
H	6.29	2.10(32'), 2.10(32''), 16.40(29)	H3	1.36	7.00(60)
29 C	126.28		75 N		
H	6.52	16.40(28), 4.80(32'), 4.80(32'')	H	8.40	7.80(60)
30 N			76 O		
H	8.53	9.93(25)	H	4.26	
31 C	170.57		77 C	170.66	
32 C	39.11		78 C	58.63	
H'	2.73	2.10(28), 4.80(29), 15.70(32'')	H	4.41	8.10(79'), 8.10(79'')
H''	3.12	2.10(28), 4.80(29), 15.70(32')	79 C	37.78	
34 C	123.27		H'	2.01	8.10(78)
H	8.17	8.70(27)	H''	1.77	8.10(78)
35 C	146.25		80 C	68.79	
36 C	123.27		H	4.26	
H	8.17	8.70(37)	81 C	56.35	
37 C	130.5		H'	3.60	
H	7.59	8.70(36)	H''		
38 C	146.59		84 C	35.22	
39 C	19.84		85 C	26.46	
H3	1.51	7.20(22)	H3	0.91	
40 C	36.05		86 C	26.46	
H'	2.92	9.80(25), 14.20(40'')	H3	0.91	
H''	2.92	4.90(25), 14.20(40')	87 C	26.46	
42 C	172.74		H3	0.91	

Table S2 – Assignments of CG167 in DMSO-d_6. Numbering scheme of the molecule is shown above the table. Assignments were made based on COSY, HSQC, HMBC and NOESY spectra. Coupling constant values *J* are given with their coupling partner shown in parentheses.

CG167 NOE analysis

NOE crosspeaks were analysed to suggest a conformation of the molecule and confirm relative arrangement of stereocenter 11 and 15 (Supplementary Fig. 4 **a**). As the two stereocenters 11 and 15 are expected to be *anti* to each other, there is no NOE between these hydrogens and one must rely on indirect NOEs to further confirm the structure.

Peaks are often overlapping due to the complex structure of CG167 but HSQC enables quite accurate assignment of peak regions and NOE cross peaks are sharp. The most useful NOEs were obtained from the quinoline moiety, enabling the unequivocal assignment of its conformation: H5 is the only aromatic proton which interacts with the other side of the macrocycle, namely with methyl group 39 and its α-proton 22.This indicates that the quinoline nitrogen faces towards the inside of the macrocycle, as expected from studies by Mackman *et al* and crystal structures of the molecules[5, 13]. The arrangement of the double bond 28-29 can then be deducted as H1 exclusively and strongly interacts with H29, whereas H5 with H28.

The pyridazic ring moiety can be further elucidated, with methyl group 39 giving a strong NOE to H15, in accordance with the (S) configuration of the stereocenter 15. H15 in turn gives a strong NOE to H18'' and none or weak ones to H17/H17'', indicating H15 and H18'' are likely both axial and on the same side of the ring. NOEs between H18'' and the methyl group 39 are difficult to distinguish due to diagonal peaks.

H11, the stereocenter resulting from the Noyori reduction, interacts in turn strongly with one single aromatic proton, H8. Furthermore, H11 has a clear absence of NOE to H12'', and in turn a strong NOE to H12' and/or H52, enabling the assignment of protons 12. Out of both protons, H12'' exclusively interacts with both hydrogens 17, further confirming the proposed conformation and the (R)-stereocenter 11.

Conformer generation of the molecule with the inverted (S) stereocenter at position 11 showed that while most of the NOESYs could be satisfied in several conformers, methyl group 39 and methylene 12 could not be arranged in a way complying with the experimental data (not shown).

Finally, alignment shown in supplementary Fig. 4 **b** of the NMR predicted conformation (pink) over the molecule in its binding mode docked as in Figure 1 shows a close overlay. Taken together, this experimental data and the biological affinity to CypA strongly supports the predicted stereocenters.

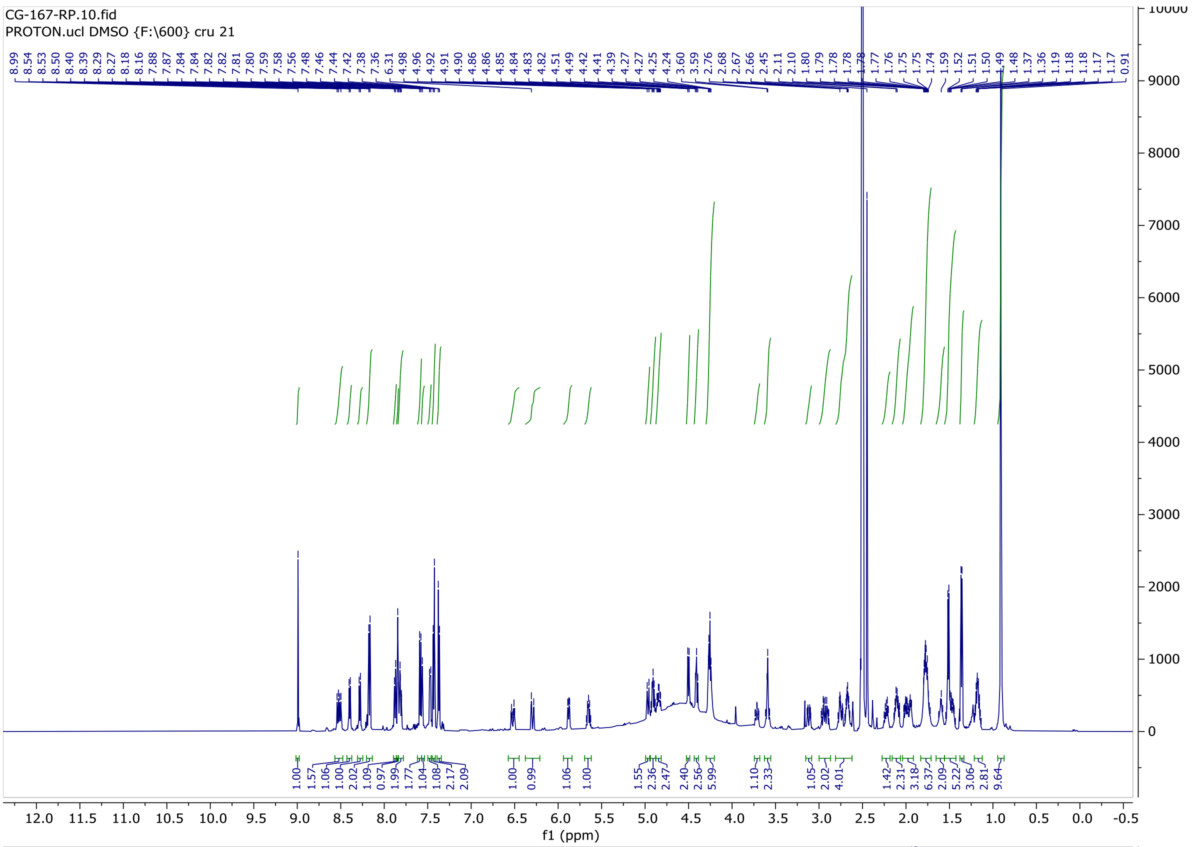

CG-167-RP.10.fid
PROTON.ucl DMSO {F:\600} cru 21
f1 (ppm)

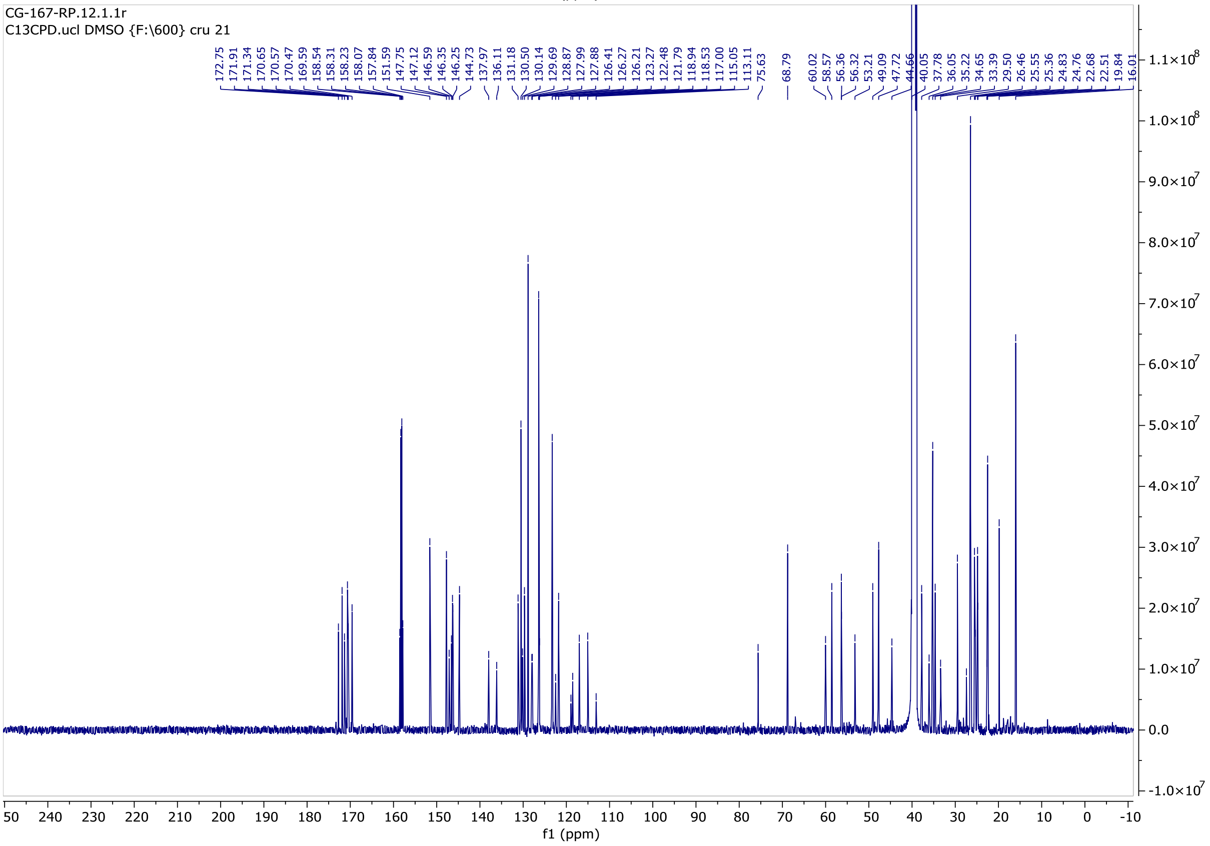

CG-167-RP.12.1.1r
C13CPD.ucl DMSO {F:\600} cru 21
f1 (ppm)

(2S,4R)-1-((2S)-3,3-dimethyl-2-(6-(4-(((53S,2R,7S,10S,E)-2-methyl-10-(4-nitrobenzyl)-4,6,9,12-tetraoxo-51,52,53,54,55,56-hexahydro-3-oxa-8,11-diaza-1(2,7)-quinolina-5(3,1)-pyridazinacyclopentadecaphan-14-en-7-yl)methyl)-1H-1,2,3-triazol-1-yl)hexanamido)butanoyl)-4-hydroxy-N-((S)-1-(4-(4-methylthiazol-5-yl)phenyl)ethyl)pyrrolidine-2-carboxamide (RJS308)

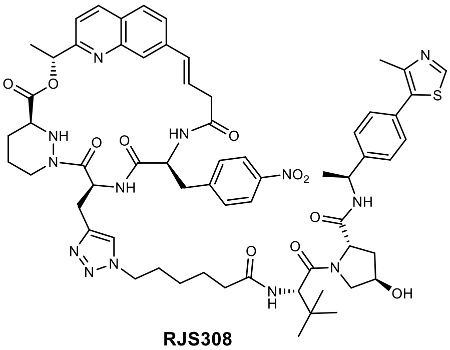

RJS308

To a solution of carboxylic acid **s19** (20.0 mg, 25 µmol, 1.00 eq) in dry DMF (1 mL) was added the VHL ligand 2 ((S,R,S)-AHPC-Me) (13.0 mg, 25 µmol, 1.00 eq), HATU (9.6 mg, 25 µmol, 1.00 eq) and *i*Pr_2NEt (9 µL, 50 µmol, 2.00 eq). The reaction was stirred for 15h at 25°C, after which the reaction stagnated. The reaction was concentrated under reduced pressure and directly purified by flash chromatography (C18, 10-100% MeOH/H_2O, 0.1% TFA, twice) to provide the PROTAC **RJS308** (10.0 mg, TFA salt, 7.5 µmol, 30% yield).

^1H NMR (600 MHz, DMSO-d$_6$) δ 9.00 (s, 1H), 8.78 (d, *J* = 8.7 Hz, 1NH), 8.55 (d, *J* = 9.5 Hz, 1NH), 8.43 – 8.37 (m, 1NH), 8.30 (d, *J* = 8.5 Hz, 1H), 8.27 – 8.24 (m, 1H), 8.22 – 8.15 (m, 2H), 7.84 (d, *J* = 8.6 Hz, 1H), 7.76 (d, *J* = 9.5 Hz, 1NH), 7.77 – 7.71 (m, 1H), 7.62 (d, *J* = 8.7 Hz, 2H), 7.50 (d, *J* = 8.5 Hz, 1H), 7.43 (d, *J* = 8.3 Hz, 2H), 7.37 (d, *J* = 8.3 Hz, 2H), 7.32 (s, 1H), 6.42 (dt, *J* = 16.5, 5.0 Hz, 1H), 6.26 (dt, *J* = 16.6, 2.0 Hz, 1H), 6.04 (td, *J* = 8.6, 3.8 Hz, 1H), 5.99 (q, *J* = 6.8 Hz, 1H), 5.04 (d, *J* = 12.3 Hz, 1NH), 4.92 (q, *J* = 6.8 Hz, 1H), 4.89 (td, *J* = 11.0, 8.6, 4.3 Hz, 1H), 4.55 – 4.48 (m, 1H), 4.48 (d, *J* = 9.4 Hz, 1H), 4.40 (t, *J* = 8.1 Hz, 1H), 4.31 – 4.25 (m, 1H), 4.27 – 4.21 (m, 2H), 4.13 – 3.95 (m, 1H), 3.81 (td, *J* = 11.9, 2.9 Hz, 1H), 3.63 – 3.55 (m, 2H), 3.21 (dd, *J* = 15.1, 9.0 Hz, 1H), 3.17 – 3.09 (m, 1H), 3.10 – 3.01 (m, 1H), 2.98 (dd, *J* = 13.8, 4.3 Hz, 1H), 2.91 (dd, *J* = 13.9, 11.0 Hz, 1H), 2.88 – 2.79 (m, 1H), 2.76 (td, *J* = 15.6, 13.9, 6.4 Hz, 1H), 2.45 (s, 3H), 2.27 – 2.09 (m, 2H), 2.04 – 1.93 (m, 3H), 1.83 – 1.70 (m, 3H), 1.67 (d, *J* = 6.8 Hz, 3H), 1.65 – 1.50 (m, 2H), 1.46 (dt, *J* = 14.1, 7.4 Hz, 1H), 1.35 (d, *J* = 6.9 Hz, 3H), 1.31 (dd, *J* = 14.1, 7.5 Hz, 1H), 1.27 – 1.09 (m, 1H), 1.04 (dp, *J* = 14.8, 7.5 Hz, 1H), 0.87 (s, 9H).

^{13}C NMR (151 MHz, DMSO-d$_6$) δ 171.84, 171.37, 171.33, 171.10, 170.64 (2x), 169.58, 158.64, 151.63, 158.33 (q, TFA), 147.66, 146.60, 146.52, 146.29, 144.75, 142.45, 137.89, 136.52, 131.21, 130.83, 130.54, 129.65, 128.86, 127.79, 127.63, 126.40, 126.11, 125.93, 124.71, 123.28, 122.58, 118.18, 115.37 (q, TFA)71.92, 68.78, 59.44, 58.55, 56.31, 53.52, 49.01, 48.42, 47.72, 40.43, 39.5 (under DMSO), 37.77, 36.29, 35.24, 35.20, 34.65, 29.57, 29.18, 27.45, 26.40, 25.56, 24.80, 22.50, 20.00, 15.97.

HRMS (ESI+): calcd. for $[C_{63}H_{75}N_{13}O_{11}S+2H]^{2+}$ (MH_2^{2+}) 611.7788; found 611.7788

LC trace (254 nM)

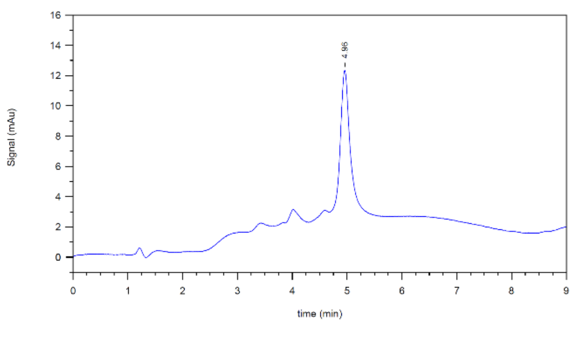

RJS308 assignments

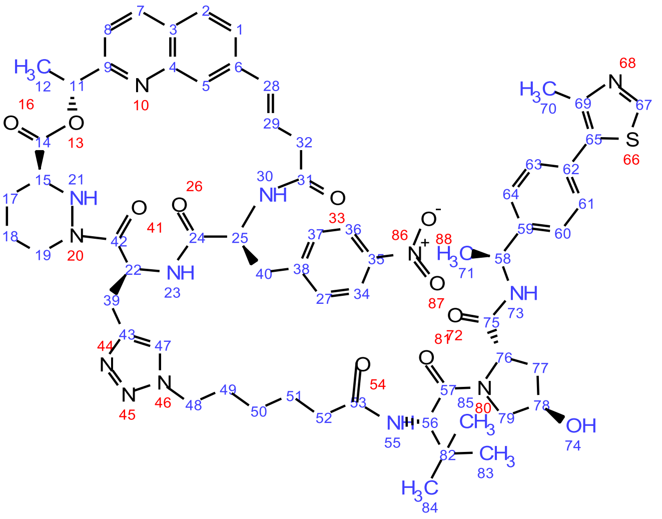

Atom	δ (ppm)	J (Hz)	Atom	δ (ppm)	J (Hz)
1 C	122.58		43 C	142.45	
H	7.74	8.60(2)	47 C	124.71	
2 C	127.63		H	8.26	
H	7.84	8.60(1)	48 C	49.01	
3 C	126.11		H'	4.24	
4 C	146.52		H"	4.02	
5 C	127.79		49 C	29.18	
H	7.32		H'	1.77*	7.50(50)
6 C	137.89		H"	1.59*	7.50(50)
7 C	136.52	8.50(8)	50 C	25.56	
H	8.3		H'	1.04	7.50(49), 14.80(50"), 7.50(51)
8 C	118.18	8.50(7)	H"	1.2	7.50(51), 14.80(50')
H	7.5		51 C	24.8	
9 C	158.64		H'	1.31	7.50(52), 14.10(51'), 7.50(50)
11 C	71.92		H"	1.46	57.40(50), 14.10(51'), 7.50(52)
H	5.99	6.80(12)	52 C	35.24	
12 C	20		H'	2.17*	7.50(51)
H3	1.67	6.80(11)	H"	2.00*	7.50(51)
14 C	171.1		53 C	171.84	
15 C	59.44		55 N		
H	3.81	11.80(18'), 2.94(18"), 12.00(21)	H	7.76	9.50(56)
17 C	34.65		56 C	56.31	
H'	2.00*	13.90(19")	H	4.48	9.50(55)
H"	2.17*	6.40(19")	57 C	169.58	
18 C	27.45		58 C	47.72	
H'	1.77*	11.80(15)	H	4.92	6.80(71)
H"	1.59*	2.94(15)	59 C	144.75	
19 C	40.43		60 C	126.4	8.30(61), 8.30(63)
H'	4.24	15.60(19")	H	7.37	
H"	2.76	13.90(17'), 6.40(17"), 15.60(19')	61 C	128.86	8.30(60), 8.30(64)
21 N			H	7.43	
H	5.04	12.00(15)	62 C	131.21	
22 C	48.42		63 C	128.86	8.30(60), 8.30(64)
H	6.04	8.70(23), 3.80(39"), 8.80(39')	H	7.43	
23 N			64 C	126.4	8.30(61), 8.30(63)
H	8.78	8.70(22)	H	7.37	
24 C	170.64		65 C	129.65	
25 C	53.52		67 C	151.63	
H	4.89	8.55(30), 11.00(40"), 4.30(40')	H	9	
27 C	130.54		69 C	147.66	

H	7.62	8.70(36), 8.70(34)	70 C	15.97	
28 C	130.83		H3	2.45	
H	6.26	16.50(29), 2.00(32'), 2.00(32")	71 C	22.5	
29 C	125.93		H3	1.35	6.80(58)
H	6.42	5.00(32'), 5.00(32"), 16.50(28)	73 N		
30 N			H	8.39	
H	8.55	8.55(25)	74 O		
31 C	171.37		H	unclear	
32 C	39.98		75 C	170.64	
H'	3.06	5.00(29), 2.00(28)	76 C	58.55	
H"	2.84	5.00(29), 2.00(28)	H	4.4	8.07(77'), 8.07(77")
34 C	123.28		77 C	37.77	
H	8.18	8.70(37), 8.70(27)	H'	2.00*	8.07(76)
35 C	146.29		H"	1.77*	8.07(76)
36 C	123.28		78 C	68.78	
H	8.18	8.70(37), 8.70(27)	H	4.27	
37 C	130.54		79 C	56.3	
H	7.62	8.70(36), 8.70(34)	H'	3.59	
38 C	146.6		H"		
39 C	29.57		82 C	35.2	
H'	3.21	8.80(22), 15.00(39")	83 C	26.4	
H"	3.13	3.80(22), 15.00(39')	H3	0.87	
40 C	36.29		84 C	26.4	
H'	2.98	4.30(25), 13.90(40")	H3	0.87	
H"	2.91	11.00(25), 13.90(40')	85 C	26.4	
42 C	171.33		H3	0.87	

Table S3 – Assignments of RJS308 in DMSO-d_6. Numbering scheme of the molecule is shown above the table. Assignments were made based on COSY, HSQC, HMBC and NOESY spectra. Coupling constant values *J* are given with their coupling partner shown in parentheses. **Shifts could not be unambiguously determined and are approximated based on PROTAC CG167.*

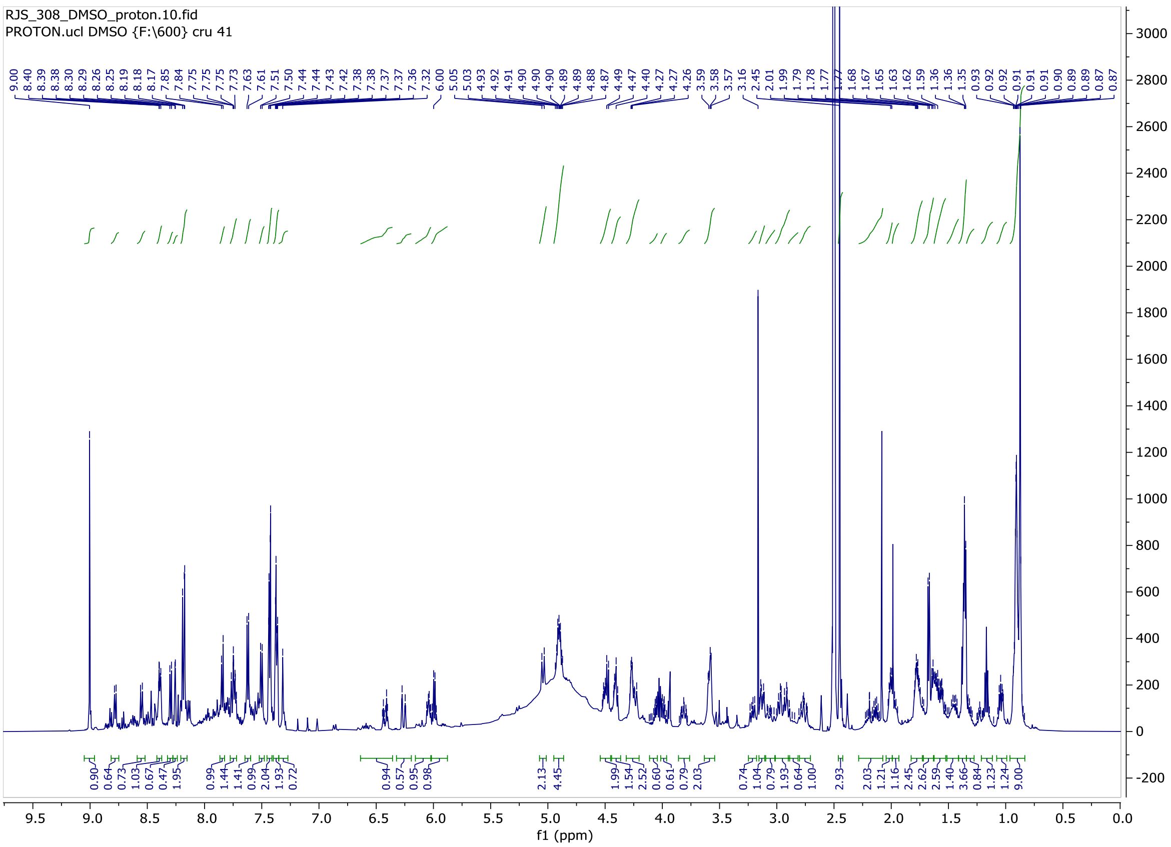

RJS_308_DMSO_proton.10.fid
PROTON.ucl DMSO {F:\600} cru 41
f1 (ppm)

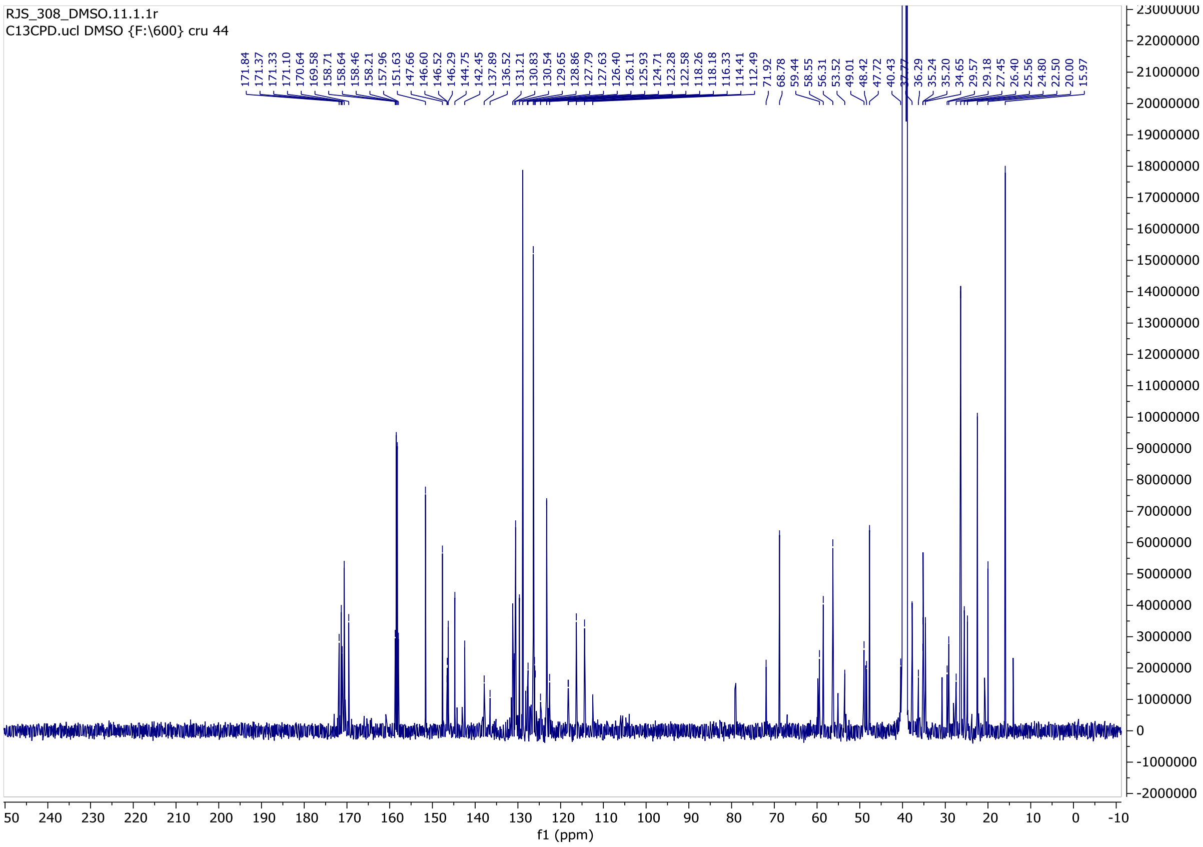

RJS_308_DMSO.11.1.1r
C13CPD.ucl DMSO {F:\600} cru 44
f1 (ppm)

References

1. Fehr, T., Kallen, J., Oberer, L., Sanglier, J.J. & Schilling, W. Sanglifehrins A, B, C and D, novel cyclophilin-binding compounds isolated from Streptomyces sp. A92-308110. II. Structure elucidation, stereochemistry and physico-chemical properties. *J. Antibiot. (Tokyo)* **52**, 474-479 (1999).
2. Sanglier, J.J. *et al.* Sanglifehrins A, B, C and D, novel cyclophilin-binding compounds isolated from Streptomyces sp. A92-308110. I. Taxonomy, fermentation, isolation and biological activity. *J. Antibiot. (Tokyo)* **52**, 466-473 (1999).
3. Hansson, M.J. *et al.* Bioengineering and semisynthesis of an optimized cyclophilin inhibitor for treatment of chronic viral infection. *Chem. Biol.* **22**, 285-292 (2015).
4. Steadman, V.A. *et al.* Discovery of potent cyclophilin inhibitors based on the structural simplification of Sanglifehrin A. *J. Med. Chem.* **60**, 1000-1017 (2017).
5. Mackman, R.L. *et al.* Discovery of a Potent and Orally Bioavailable Cyclophilin Inhibitor Derived from the Sanglifehrin Macrocycle. *J. Med. Chem.* **61**, 9473-9499 (2018).
6. Wang, P. & Heitman, J. The cyclophilins. *Genome Biol.* **6**, 226 (2005).
7. Davis, T.L. *et al.* Structural and biochemical characterization of the human cyclophilin family of peptidyl-prolyl isomerases. *PLoS Biol.* **8**, e1000439 (2010).
8. Love, B.E. & Jones, E.G. The Use of Salicylaldehyde Phenylhydrazone as an Indicator for the Titration of Organometallic Reagents. *J. Org. Chem.* **64**, 3755-3756 (1999).
9. Graham, S.L. & Scholz, T.H. A New Mode of Reactivity of N-Methoxy-N-Methylamides with Strongly Basic Reagents. *Tetrahedron Lett.* **31**, 6269-6272 (1990).
10. Mackman, R.L. *et al.* Discovery of a potent and orally bioavailable cyclophilin inhibitor derived from the Sanglifehrin macrocycle. *J. Med. Chem.* **61**, 9473-9499 (2018).
11. Hale, K.J., Delisser, V.M. & Manaviazar, S. Azinothricin Synthetic Studies .1. Efficient Asymmetric Syntheses of (3r)-Piperazic and (3s)-Piperazic Acids. *Tetrahedron Lett.* **33**, 7613-7616 (1992).
12. Hale, K.J. & Cai, J.Q. Synthetic studies on the azinothricin family of antitumour antibiotics .5. Asymmetric synthesis of two activated esters for the northern sector of A83586C. *Tetrahedron Lett.* **37**, 4233-4236 (1996).
13. Steadman, V.A. *et al.* Discovery of Potent Cyclophilin Inhibitors Based on the Structural Simplification of Sanglifehrin A. *J. Med. Chem.* **60**, 1000-1017 (2017).
