# Supplementary material for: Macrocycle-based PROTACs selectively degrade cyclophilin A and inhibit HIV-1 and HCV
Source: Nat Commun. 2025 Feb 10;16:1484. doi: 10.1038/s41467-025-56317-8 (PMC11811207; doi:10.1038/s41467-025-56317-8)
Supplement: Supplementary file 2 — Reporting Summary [file 41467_2025_56317_MOESM2_ESM.pdf]

nature portfolio

Corresponding author(s): David Selwood
Greg Towers

Last updated by author(s): 2024/12/04

Reporting Summary

Nature Portfolio wishes to improve the reproducibility of the work that we publish. This form provides structure for consistency and transparency in reporting. For further information on Nature Portfolio policies, see our Editorial Policies and the Editorial Policy Checklist.

Statistics

For all statistical analyses, confirm that the following items are present in the figure legend, table legend, main text, or Methods section.

n/a	Confirmed	
☐	☒	The exact sample size (*n*) for each experimental group/condition, given as a discrete number and unit of measurement
☐	☒	A statement on whether measurements were taken from distinct samples or whether the same sample was measured repeatedly
☐	☒	The statistical test(s) used AND whether they are one- or two-sided<br>*Only common tests should be described solely by name; describe more complex techniques in the Methods section.*
☐	☒	A description of all covariates tested
☐	☒	A description of any assumptions or corrections, such as tests of normality and adjustment for multiple comparisons
☐	☒	A full description of the statistical parameters including central tendency (e.g. means) or other basic estimates (e.g. regression coefficient) AND variation (e.g. standard deviation) or associated estimates of uncertainty (e.g. confidence intervals)
☐	☒	For null hypothesis testing, the test statistic (e.g. *F*, *t*, *r*) with confidence intervals, effect sizes, degrees of freedom and *P* value noted<br>*Give P values as exact values whenever suitable.*
☒	☐	For Bayesian analysis, information on the choice of priors and Markov chain Monte Carlo settings
☒	☐	For hierarchical and complex designs, identification of the appropriate level for tests and full reporting of outcomes
☒	☐	Estimates of effect sizes (e.g. Cohen's *d*, Pearson's *r*), indicating how they were calculated

Our web collection on statistics for biologists contains articles on many of the points above.

Software and code

Policy information about availability of computer code

Data collection	NovoSampler Pro (Agilent)<br>LI-COR Odyssey<br>Glomax (Promega)<br>Biacore T200<br>Biacore 8K instruments (Cytiva)<br>AKTA Pure system (Cytiva)<br>PHERAstar FSX (BMG LABTECH)<br>Ultimate3000 high-performance liquid chromatography system<br>Eclipse mass spectrometer (Thermo Fisher Scientific)
Data analysis	GraphPad Prism (v9.5.1)<br>FlowJo v10.10 (Tree Star)<br>NovoExpress 1.5.0 software (Agilent)<br>ImageJ2 (v2.14.0)<br>Origin (9.8.0.200)<br>Mestrenova (14.3.1)<br>MOE (2020.0901)<br>Biacore Insight software<br>DIA-NN v1.8.1

Perseus version 2.0.10.0

For manuscripts utilizing custom algorithms or software that are central to the research but not yet described in published literature, software must be made available to editors and reviewers. We strongly encourage code deposition in a community repository (e.g. GitHub). See the Nature Portfolio guidelines for submitting code & software for further information.

Data

Policy information about availability of data

All manuscripts must include a data availability statement. This statement should provide the following information, where applicable:

- Accession codes, unique identifiers, or web links for publicly available datasets
- A description of any restrictions on data availability
- For clinical datasets or third party data, please ensure that the statement adheres to our policy

All raw data for the main figures are provided in the Source Data file provided with this paper. Raw data for the supplementary figures is available in the Figshare research data repository 10.6084/m9.figshare.c.7387381. Characterisation data for synthesised compounds including LC traces, NMR and MS are provided in the Supplementary information. The associated raw data and processed NMRs are available in the Figshare research data repository 10.6084/m9.figshare.c.7387381. The mass spectrometry proteomics data have been deposited to the ProteomeXchange Consortium via the PRIDE87 partner repository with the dataset identifier PXD057024 (https://proteomecentral.proteomexchange.org/cgi/GetDataset?ID=PXD057024). Molecular modelling studies were performed using following accession codes: 1YND (https://doi.org/10.2210/pdb1YND/pdb), 1CWA (https://doi.org/10.2210/pdb1CWA/pdb). Supporting files for molecular modelling and are available in the Figshare research data repository 10.6084/m9.figshare.c.7387381.

Research involving human participants, their data, or biological material

Policy information about studies with human participants or human data. See also policy information about sex, gender (identity/presentation), and sexual orientation and race, ethnicity and racism.

Reporting on sex and gender	Primary cells isolated from human blood (UK NHS Blood and Transplant Service or from fresh blood from healthy volunteers) were used in this study. This process was completely anonymous and information on sex and gender was not collected.
Reporting on race, ethnicity, or other socially relevant groupings	Primary cells isolated from human blood (UK NHS Blood and Transplant Service or from fresh blood from healthy volunteers) were used in this study. This process was completely anonymous and information on race, ethnicity, or other socially relevent groupings was not collected.
Population characteristics	Primary cells isolated from human blood (UK NHS Blood and Transplant Service or from fresh blood from healthy volunteers) were used in this study. This process was completely anonymous and information on these characteristics was not collected.
Recruitment	For primary cells isolated from human blood from the UK NHS Blood and Transplant Service we were not involved in recruitment and have no information about these individuals. For primary cells isolated from human blood from healthy volunteers, here is the relevant information from the ethics document: Participants will be recruited through poster advertising for healthy adults aged 18-65, and must be able to give written informed consent. We will recruit adults who are able to give written informed consent autonomously, and below the age of 65 to avoid age related confounding of immune cell biology. Participants will be recruited by poster advertising within UCL.
Ethics oversight	The study was approved by the joint UCL/UCLH NHS Trust Human Research Ethics committee, and written informed consent obtained from all participants.

Note that full information on the approval of the study protocol must also be provided in the manuscript.

Field-specific reporting

Please select the one below that is the best fit for your research. If you are not sure, read the appropriate sections before making your selection.

[x] Life sciences [ ] Behavioural & social sciences [ ] Ecological, evolutionary & environmental sciences

For a reference copy of the document with all sections, see nature.com/documents/nr-reporting-summary-flat.pdf

Life sciences study design

All studies must disclose on these points even when the disclosure is negative.

Sample size	Sample sizes for biological replicates (provided in the figure legends) were determined following pilot experiments to obtain estimates of variance in each assay.
Data exclusions	One dataset testing TWH106 against HIV-1 GFP in U87 cells was removed from analysis due to poor activity as a result of improper storage.<br>One dataset testing compounds against HIV-1 spreading infection against one donor was removed from analysis due to unusually high levels of infection which skewed results.<br>One dataset of rescue experiments (MLN4924 inhibitor and TWH106 competition experiments, both with CG167 and RJS308) which was included in the preprint of the manuscript were excluded from analysis as repeats of the experiment made it clear that this dataset was an outlier.

Replication	At least 2 independent experiments were performed for each dataset. Reproducibility confirmed the final conclusions and in some cases statistical analysis supported hypotheses reported. All attempts at replication were successful except those listed above in the data exclusions section.<br>Exceptions to this were as follows, due to limited amounts of compound:<br>FP experiments with HIF-1α peptide bound to VCB displaced by CG167 or RJS308 (3 technical replicates)<br>MTT viability assay in Huh7 cells (1 biological replicate, performed in triplicate)
Randomization	Experimental groups are identical except for the specific variable being tested and therefore randomisation is not required.
Blinding	Blinding was not required because all outcomes are measured objectively by automated machines.

Reporting for specific materials, systems and methods

We require information from authors about some types of materials, experimental systems and methods used in many studies. Here, indicate whether each material, system or method listed is relevant to your study. If you are not sure if a list item applies to your research, read the appropriate section before selecting a response.

Materials & experimental systems

n/a	Involved in the study
☐	☒ Antibodies
☐	☒ Eukaryotic cell lines
☒	☐ Palaeontology and archaeology
☒	☐ Animals and other organisms
☒	☐ Clinical data
☒	☐ Dual use research of concern
☒	☐ Plants

Methods

n/a	Involved in the study
☒	☐ ChIP-seq
☐	☒ Flow cytometry
☒	☐ MRI-based neuroimaging

Antibodies

Antibodies used	Anti-CypA (BML-SA296-0100, Enzo, 1:2000)<br>Anti-CypB (ab16045, Abcam, 1:1400)<br>Anti-b-actin (clone AC-15, ab6276, Abcam, 1:10000)<br>Anti-VHL (sc135657, Santa Cruz Biotechnology, 1:100)<br>IRDye® 680LT goat anti-mouse (926-68020, LI-COR Biosciences, 1:15000)<br>IRDye® 680LT goat anti-rabbit (926-68021, LI-COR Biosciences, 1:15000)<br>IRDye® 800CW goat anti-mouse (926-32210, LI-COR Biosciences, 1:10000)<br>IRDye® 800CW goat anti-rabbit (926-32211, LI-COR Biosciences, 1:10000)<br>Anti-CypA (clone 1F4-1B5, ab58144, Abcam, 1:1000-1:2000)<br>Alexa Fluor® 488 goat anti-mouse (405319, Biolegend, 1:400)<br>Alexa Fluor® 647 goat anti-mouse (405322, BioLegend, 1:400)<br>FITC anti-HIV-1 Gag (KC57-FITC, clone FH190-1-1, 6604665, Beckman Coulter, 1:100)<br>APC anti-CD3 (SK7, 981012, Biolegend, 1:100)<br>PE anti-CD4 (SK3, 980804, Biolegend, 1:100)
Validation	Anti-CypA (BML-SA296-0100, Enzo, 1:2000): Enzo provides evidence for use of antibody by western blot and cites 4 examples of published use: https://www.enzolifesciences.com/fileadmin/reports/Datasheet-BML-SA296.pdf<br>Anti-CypB (ab16045, Abcam, 1:1400): Abcam provides evidence for use of antibody by western blot and cites 90 examples of published use: https://www.abcam.com/cyclophilin-b-antibody-ab16045.html<br>Anti-b-actin (clone AC-15, ab6276, Abcam, 1:10000): Abcam provides evidence for use of antibody by western blot and cites 1949 examples of published use: https://www.abcam.com/beta-actin-antibody-ac-15-ab6276.html<br>Anti-VHL (sc135657, Santa Cruz Biotechnology, 1:100): Santa Cruz provides evidence for use of antibody by western blot and cites 8 examples of published use: https://datasheets.scbt.com/sc-135657.pdf<br>IRDye® 680LT goat anti-mouse (926-68020, LI-COR Biosciences, 1:15000): LI-COR provides evidence for use of antibody by western blot: https://www.licor.com/bio/reagents/irdye-680lt-goat-anti-mouse-igg-secondary-antibody<br>IRDye® 680LT goat anti-rabbit (926-68021, LI-COR Biosciences, 1:15000): LI-COR provides evidence for use of antibody by western blot: https://www.licor.com/bio/reagents/irdye-680lt-goat-anti-rabbit-igg-secondary-antibody<br>IRDye® 800CW goat anti-mouse (926-32210, LI-COR Biosciences, 1:10000): LI-COR provides evidence for use of antibody by western blot: https://www.licor.com/bio/reagents/irdye-800cw-goat-anti-mouse-igg-secondary-antibody<br>IRDye® 800CW goat anti-rabbit (926-32211, LI-COR Biosciences, 1:10000): LI-COR provides evidence for use of antibody by western blot: https://www.licor.com/bio/reagents/irdye-800cw-goat-anti-rabbit-igg-secondary-antibody<br>Anti-CypA (clone 1F4-1B5, ab58144, Abcam, 1:1000-1:2000): Abcam provides evidence for use of antibody by FACS and cites 19 examples of published use: https://www.abcam.com/cyclophilin-a-antibody-1f4-1b5-ab58144.html<br>Alexa Fluor® 488 goat anti-mouse (405319, Biolegend, 1:400): Biolegend cites 26 examples of published use: https://www.biolegend.com/en-gb/products/alexa-fluor-488-goat-anti-mouse-igg-minimal-x-reactivity-9282<br>Alexa Fluor® 647 goat anti-mouse (405322, BioLegend, 1:400): BioLegend cites 16 examples of published use: https://

www.biolegend.com/en-us/products/alexa-fluor-647-goat-anti-mouse-igg-minimal-x-reactivity-9283?GroupID=BLG2049
FITC anti-HIV-1 Gag (KC57-FITC, clone FH190-1-1, 6604665, Beckman Coulter, 1:100): Beckman Coulter cites 3 examples of published use: https://www.bc-cytometry.com/PDF/DataSheet/6604665&6604667%20D.S.pdf
APC anti-CD3 (SK7, 981012, Biolegend): BioLegend provides evidence for use of antibody by FACS: https://www.biolegend.com/en-gb/products/apc-anti-human-cd3-19893
PE anti-CD4 (SK3, 980804, Biolegend): BioLegend provides evidence for use of antibody by FACS: https://www.biolegend.com/en-us/products/pe-anti-human-cd4-antibody-11857

Eukaryotic cell lines

Policy information about cell lines and Sex and Gender in Research

Cell line source(s)	Calu-3 (ATCC HTB-55) (male)<br>HEK 293T (ATCC CRL-3216) (sex not specified)<br>Huh7 (from Joe Grove (UCL), originally obtained from Dr. Yoshiharu Matsuura (from Japanese Collection of Research Bioresources Cell Bank, JCRB0403)) (male)<br>U87 CCR5 expressing cells (ATCC HTB-14) (male)<br>Jurkat T cells (Clone E6-1; ATCC TIB-152) (male)<br>THP-1 Dual reporter cells (Invivogen) (male)<br>Primary MDMs and primary T cells were derived from PBMCs isolated from buffy coats or leukocyte cones from healthy donors (UK NHS Blood and Transplant Service) or from fresh blood from healthy volunteers. This process was completely anonymous so the sex is not known and cannot be reported.
Authentication	All cell lines were originally purchased from ATCC or Invivogen, or obtained from the Japanese Cell Bank. These have rigorous standards for cell line authentication using short tandem repeat profiling. This confirms the identify of cells and detects misidentified, cross-contaminated, or genetically drifted cells.
Mycoplasma contamination	All cell lines were tested negative for mycoplasma.
Commonly misidentified lines (See ICLAC register)	No commonly misidentified cell lines were used in this study.

Plants

Seed stocks	This study did not involve plants
Novel plant genotypes	This study did not involve plants
Authentication	This study did not involve plants

Flow Cytometry

Plots

Confirm that:

- [x] The axis labels state the marker and fluorochrome used (e.g. CD4-FITC).
- [x] The axis scales are clearly visible. Include numbers along axes only for bottom left plot of group (a 'group' is an analysis of identical markers).
- [x] All plots are contour plots with outliers or pseudocolor plots.
- [x] A numerical value for number of cells or percentage (with statistics) is provided.

Methodology

Sample preparation	For HIV-1 GFP lentiviral infection of U87 and Jurkat cells, cells were fixed in 4 % formaldehyde in PBS. The % GFP positive (infected) cells was determined using a NovoSampler Pro (Agilent) flow cytometer. For primary T cell experiments, cells were washed in PBS and stained with fixable Zombie R685 Live/Dead dye (423119, Biolegend) for 5 mins at 37˚C. Excess stain was quenched with FBS-complemented RPMI. Cells were fixed with 4 % formaldehyde before intracellular staining. Permeabilisation for intracellular staining of primary T cells and Jurkat cells was performed with IC perm buffer (Biolegend) according to the manufacturer's instructions. Where appropriate, intracellular staining for HIV-1 Gag was performed for 30 mins at room temperature. Intracellular CypA was detected by incubation of permeabilised cells with CypA antibody (clone 1F4-1B5, ab58144, Abcam, 1:1000-1:2000) for 30 mins, followed by 15 min incubation with secondary anti-mouse AlexaFluor488 or AlexaFluor647-tagged antibody. Excess antibody was removed through several wash steps performed at 1700 rpm for 5 min at 4˚C. Data were acquired on a NovoSampler Pro (Agilent). For testing the purity of isolated primary CD4

+ T cells, cell surface staining with CD3-APC (981012, Biolegend) and CD4-PE (980804, Biolegend) was performed in PBS followed by analysis by flow cytometry on a NovoSampler Pro (Agilent).

Instrument	NovoSampler Pro (Agilent)
Software	FlowJo v10.10 (Tree Star)<br>NovoExpress 1.5.0 software (Agilent)
Cell population abundance	No sorting was carried out. Purity of isolated CD4+ T cells was assessed by cell surface staining with CD3-APC (981012, BioLegend) and CD4-PE (980804, BioLegend) (performed in PBS), followed by analysis by flow cytometry (≥ 95 % CD3+ CD4+ for all donors), see suppplementary figure 15.
Gating strategy	Cells were first gated to exclude cell debris (SSC-A vs FSC-A) and gated on single cells (FSC-A vs FSC-H). For Jurkats and U87s, cells were then gated on FITC-H (GFP+, threshold set using uninfected cells), and for Jurkat cells, APC-A (CypA, threshold set using cells without secondary antibody staining). For T cell infection experiments, after single cell gating, cells were regated (SSC-A vs SSC-H), then gated as follows: Live cells (SSC-A vs ZombieR685 Live/Dead) then Gag+ (infected cells) (SSC-A vs Gag-FITC). Or, for the same samples stained separately, CypA (FITC) MFI was measured. Examples of the gating strategies are given in Supplementary Information.

☒ Tick this box to confirm that a figure exemplifying the gating strategy is provided in the Supplementary Information.
